# Non-canonical functions of SNAIL drive context-specific cancer progression

**DOI:** 10.1038/s41467-023-36505-0

**Published:** 2023-03-07

**Authors:** Mariel C. Paul, Christian Schneeweis, Chiara Falcomatà, Chuan Shan, Daniel Rossmeisl, Stella Koutsouli, Christine Klement, Magdalena Zukowska, Sebastian A. Widholz, Moritz Jesinghaus, Konstanze K. Heuermann, Thomas Engleitner, Barbara Seidler, Katia Sleiman, Katja Steiger, Markus Tschurtschenthaler, Benjamin Walter, Sören A. Weidemann, Regina Pietsch, Angelika Schnieke, Roland M. Schmid, Maria S. Robles, Geoffroy Andrieux, Melanie Boerries, Roland Rad, Günter Schneider, Dieter Saur

**Affiliations:** 1 Division of Translational Cancer Research, German Cancer Research Center (DKFZ) and German Cancer Consortium (DKTK), Im Neuenheimer Feld 280, 69120 Heidelberg, Germany; 2 Chair of Translational Cancer Research and Institute of Experimental Cancer Therapy, Klinikum rechts der Isar, School of Medicine, Technische Universität München, Ismaninger Str. 22, 81675 Munich, Germany; 3 Department of Internal Medicine II, Klinikum rechts der Isar, Technische Universität München, Ismaninger Str. 22, 81675 Munich, Germany; 4 Center for Translational Cancer Research (TranslaTUM), School of Medicine, Technical University of Munich, Ismaninger Str. 22, 81675 Munich, Germany; 5 Institute of Medical Psychology, Faculty of Medicine, LMU Munich, Goethe Str. 31, 80336 Munich, Germany; 6 Institute of Molecular Oncology and Functional Genomics, School of Medicine, Technische Universität München, 81675 Munich, Germany; 7 German Cancer Consortium (DKTK) and German Cancer Research Center (DKFZ), Partner Site Munich, Munich, Germany; 8 Institute of Pathology, Technische Universität München, Ismaninger Str. 22, 81675 Munich, Germany; 9 Institute of Pathology, University Hospital Marburg, Baldingerstraße, 35043 Marburg, Germany; 10 Livestock Biotechnology, School of Life Sciences, Technische Universität München, Liesel-Beckmann Str. 1, 85354 Freising, Germany; 11 Institute of Medical Bioinformatics and Systems Medicine, Medical Center - University of Freiburg, Faculty of Medicine, University of Freiburg, 79110 Freiburg, Germany; 12 German Cancer Consortium (DKTK) and German Cancer Research Center (DKFZ), Partner Site Freiburg, 79106 Freiburg, Germany; 13 University Medical Center Göttingen, Department of General, Visceral and Pediatric Surgery, 37075 Göttingen, Germany

**Keywords:** Cancer genetics, Oncogenes, Cancer models, Senescence, Checkpoints

## Abstract

SNAIL is a key transcriptional regulator in embryonic development and cancer. Its effects in physiology and disease are believed to be linked to its role as a master regulator of epithelial-to-mesenchymal transition (EMT). Here, we report EMT-independent oncogenic SNAIL functions in cancer. Using genetic models, we systematically interrogated SNAIL effects in various oncogenic backgrounds and tissue types. SNAIL-related phenotypes displayed remarkable tissue- and genetic context-dependencies, ranging from protective effects as observed in KRAS- or WNT-driven intestinal cancers, to dramatic acceleration of tumorigenesis, as shown in KRAS-induced pancreatic cancer. Unexpectedly, SNAIL-driven oncogenesis was not associated with E-cadherin downregulation or induction of an overt EMT program. Instead, we show that SNAIL induces bypass of senescence and cell cycle progression through p16^INK4A^-independent inactivation of the Retinoblastoma (RB)-restriction checkpoint. Collectively, our work identifies non-canonical EMT-independent functions of SNAIL and unravel its complex context-dependent role in cancer.

## Abstract (web summary)

SNAIL promotes tumour metastasis through inducing epithelial to mesenchymal transition (EMT). Here the authors report that SNAIL bypasses senescence and regulates cell cycle progression to promote pancreatic carcinogenesis and this is independent of EMT induction.

## Introduction

SNAIL is overexpressed in > 70% of human pancreatic ductal adenocarcinomas (PDAC)^1^ and a wide number of other tumour entities, such as intestinal, breast, lung and liver cancer^2–6^. SNAIL expression is believed to be a key driver of tumour aggressiveness and metastasis formation via the induction of an epithelial-to-mesenchymal transition (EMT) program and the subsequent acquisition of stem cell-like features^5,7–12^. Accordingly, it is often correlated with poor prognosis and shortened survival of cancer patients. However, the specific in vivo functions of SNAIL and the role of EMT during tumour progression in different tumour types remain largely unexplored^10,13–16^. In an in vivo selection model of highly metastatic PDAC cells, we have demonstrated previously that SNAIL drives EMT and subsequently metastasis formation^17^. This runs counter to recent findings in an autochthonous mouse model of PDAC showing that *Snail* deletion does not influence the metastatic phenotype, but sensitizes tumours to chemotherapy^13^. This prompted us to re-investigate and mechanistically probe the function of SNAIL in vivo by systematic and comprehensive genetic gain- and loss-of-function approaches using a variety of disease-relevant genetically engineered autochthonous mouse models as well as human cancers.

Here, we show context-dependent oncogenic functions of the transcriptional regulator SNAIL in cancer, which are independent of its role as a regulator of the EMT process. SNAIL-induced phenotypes depend on both, the genetic context of the tumour and its tissue of origin, ranging from protective effects that delay tumour onset in intestinal cancer models to a dramatic acceleration of pancreatic cancer development and aggressiveness. Mechanistically, we demonstrate that SNAIL acts as transcriptional activator that bypasses oncogenic KRAS-induced senescence and drives the cell cycle by p16^INK4A^-independent inactivation of the Retinoblastoma (RB)-restriction checkpoint of senescence, thereby inducing context-dependent cancer progression. This knowledge provides opportunities to target SNAIL-driven cancers, which are a major clinical problem due to their high aggressiveness and lethality.

## Results

### SNAIL-driven cancer progression is highly context-specific

To investigate the function of SNAIL in different cancer types in vivo, we created a latent *Snail* allele silenced by a lox-stop-lox (LSL) cassette as a knock-in (KI) at the mouse *Rosa26* locus (*LSL-R26*^*Snail/+*^ mouse line, termed *Snail*^*KI/+*^; Supplementary Fig. S1a–c). SNAIL expression was then activated in several genetically engineered murine autochthonous cancer models: i) a PDAC model that depends on Cre-induced expression of oncogenic KRAS^G12D^ in the *Ptf1a* lineage of the pancreas (*Ptf1a*^*Cre/+*^*;LSL-Kras*^*G12D/+*^, termed *PKras*^*G12D/+*^); ii) a classical WNT-driven intestinal cancer model, induced by loss of the tumour suppressor *adenomatosis polyposis coli* (*Apc*) due to Cre-mediated deletion of a floxed *Apc* allele in intestinal epithelial cells (*Villin-Cre;Apc*^*lox/+*^, termed *VApc*^*ΔInt*^); and iii) two different models of serrated intestinal cancer driven by either oncogenic KRAS^G12D^ or BRAF^V637E^, based on *Villin-Cre*-induced activation of latent oncogenic *Kras*^*G12D*^ (*Villin-Cre;LSL-Kras*^*G12D/+*^ termed *VKras*^*G12D/+*^) or *Braf*^*V637E*^ (*Villin-Cre;LSL-Braf*^*V637E/+*^, termed *VBraf*^*V637E/+*^). In this way we mimicked the acquisition of SNAIL expression in different tumour types and subtypes driven by distinct oncogenes and signalling pathways.

**Fig. 1:**
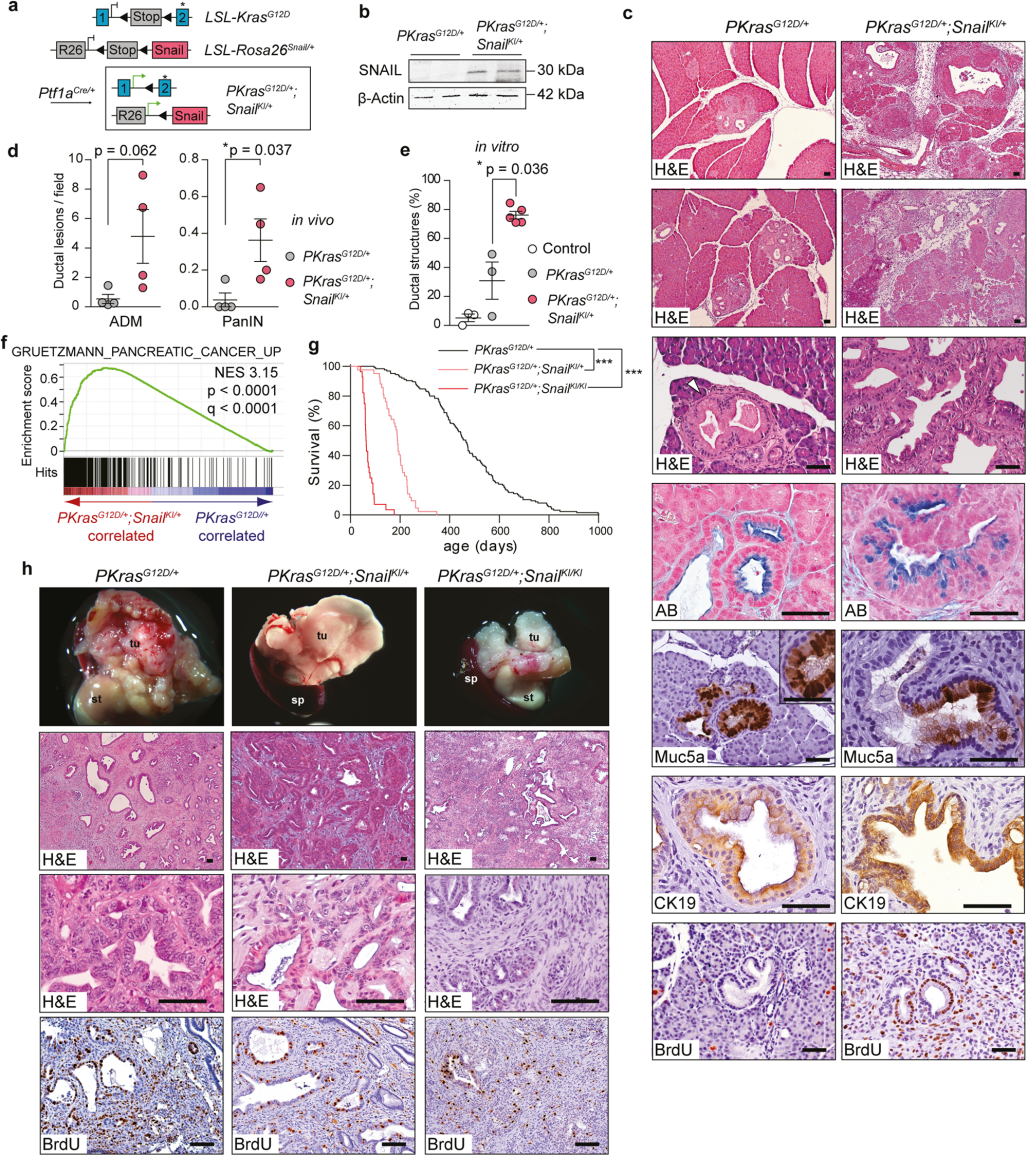
Aberrant SNAIL expression dramatically accelerates KRAS^G12D^-driven PDAC formation. **a** Genetic strategy to activate SNAIL and KRAS^G12D^ expression in the pancreas. **b** Immunoblot analysis of SNAIL protein expression in pancreas of 1-month-old *Ptf1a*^*Cre/+*^;*LSL-Kras*^*G12D/+*^ (*PKras*^*G12D/+*^) and *Ptf1a*^*Cre/+*^;*LSL-Kras*^*G12D/+*^*;LSL-R26*^*Snail/+*^ (*PKras*^*G12D/+*^*;Snail*^*KI/+*^*)* compound mutant mice. **c** Representative hematoxylin and eosin (H&E), Alcian blue (AB), Muc5a, CK19 and BrdU stains of acinar to ductal metaplasia (ADM) and pancreatic intraepithelial neoplasia (PanIN) in 1-month-old *PKras*^*G12D/+*^ and *PKras*^*G12D/+*^*;Snail*^*KI/+*^ mice. Note the almost complete remodelling of pancreatic tissue in *PKras*^*G12D/+*^*;Snail*^*KI/+*^ animals. Scale bars, 50 μm. **d** Quantification of ADM and PanIN progression in % of total lesions at age of one-month (error bars, mean ± SEM; *n* = 4 per genotype; 3 representative slides per mouse; *p = 0.037, Mann-Whitney two-tailed test). **e** Quantification of ductal and acinar structures after in vitro culture of acinar explants for 5 days (*n* = 3 control/*PKras*^*G12D/+*^ mice, *n* = 5 *PKras*^*G12D/+*^*;Snail*^*KI/+*^ mice; mean ± SEM; *p = 0.036, Mann-Whitney two-tailed test). **f** Gene-set enrichment analysis (GSEA) using mRNA expression profiles of *PKras*^*G12D/+*^*;Snail*^*KI/+*^ (red) and *PKras*^*G12D/+*^ (blue) pancreata of 1-month-old mice (*n* = 2 per genotype) computed and corrected for multiple testing using the Benjamini–Hochberg procedure (for statistical details, please see methods section). Genes were ranked using Signal-to-Noise ratio statistics according to their correlation. Vertical black lines mark the position of each gene in the data set. Normalized Enrichment Score: 3.15; Nominal *p*-value < 0.0001; False Discovery Rate (FDR) *q*-value < 0.0001. **g** Kaplan-Meier survival curves of *PKras*^*G12D/+*^ (*n* = 125; 465 days median survival), *PKras*^*G12D/+*^*;Snail*^*KI/+*^ (*n* = 42; 190 days median survival) and *PKras*^*G12D/+*^*;Snail*^*KI/KI*^ (*n* = 28; 64 days median survival) mice (***p < 0.0001, log-rank test, Bonferroni correction). **h** Macroscopic view and representative H&E and BrdU staining of pancreata with PDAC of three endpoint mice per genotype (tu tumour; st stomach; sp spleen). Scale bars, 50 µm. Note: The *PKras*^*G12D/+*^ cohort in (**g**) is the same shown in Figs. 4b, 6e, f and the *PKras*^*G12D/+*^*;Snail*^*KI/+*^ cohort is the same shown in Figs. 6e, l. Source data of Fig. 1 are provided in the Source Data file.

Concomitant transgenic expression of SNAIL and activation of oncogenic KRAS^G12D^ in the pancreas of *Ptf1a*^*Cre/+*^*;LSL-Kras*^*G12D/+*^;*LSL-R26*^*Snail/+*^ mice (termed *PKras*^*G12D/+*^*;Snail*^*KI/+*^) accelerated the formation of acinar to ductal metaplasia (ADM) and PDAC precursor lesions (pancreatic intraepithelial neoplasia (PanIN)), and substantially increased cancer development (Fig. 1a–h and Supplementary Fig. S1d–f). Consistent with this, PDAC gene set enrichment was already apparent in one-month old mice with aberrant SNAIL expression (Fig. 1f). All animals in the tumour watch cohort developed PDAC with a median survival of 190 days, compared to 465 days in *PKras*^*G12D/+*^ animals. Biallelic *Snail* expression in *Ptf1a*^*Cre/+*^*;LSL-Kras*^*G12D/+*^,*LSL-R26*^*Snail/Snail*^ mice (termed *PKras*^*G12D/+*^*;Snail*^*KI/KI*^) drastically reduced survival further to a median of only 64 days (Fig. 1g, h).

**Fig. 2:**
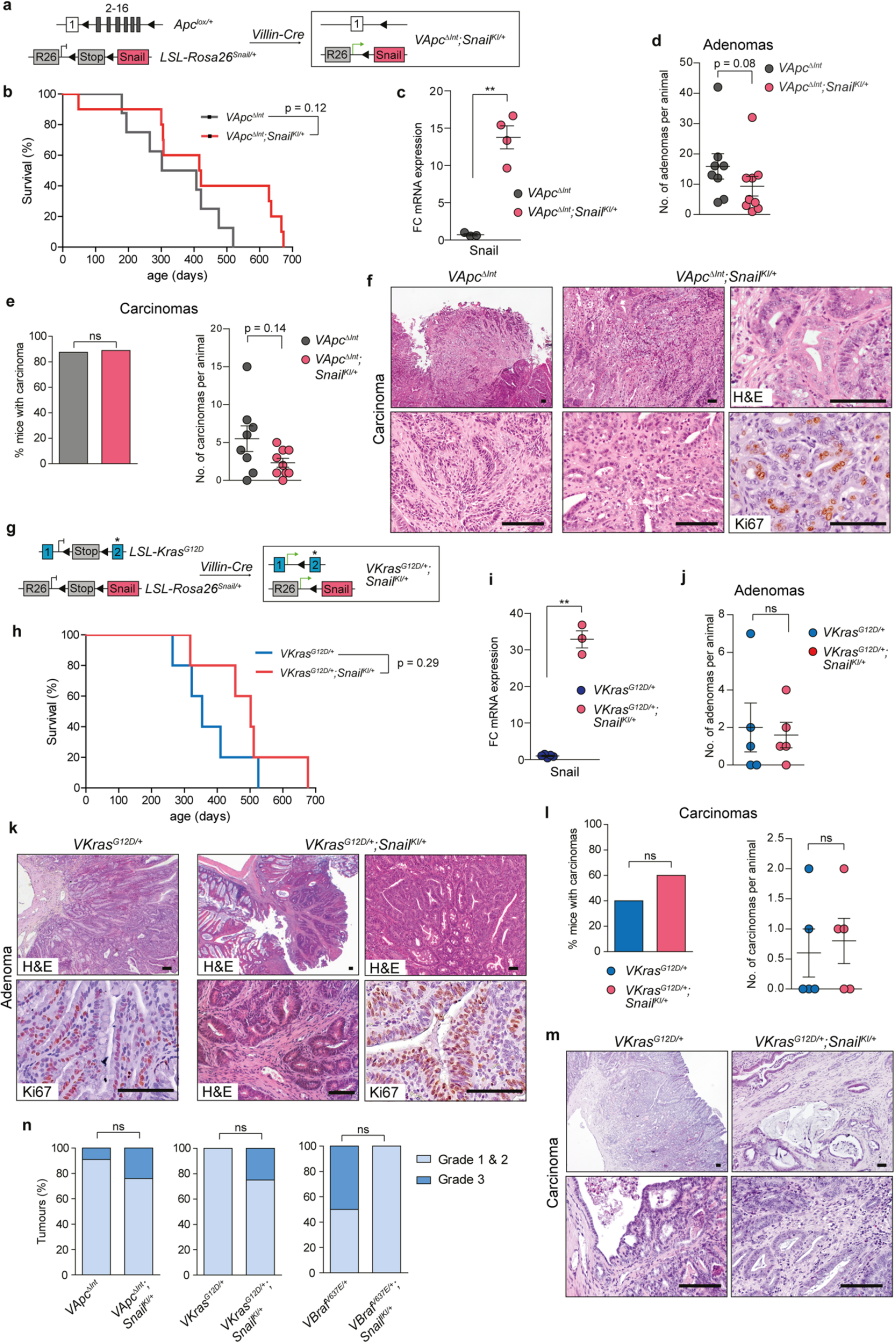
SNAIL does not promote classical APC loss-of-function and serrated KRAS^G12D^-driven intestinal cancer progression. **a** Strategy to activate Snail expression in intestinal epithelium in the *Villin-Cre;Apc*^*lox/+*^ (termed *VApc*^*ΔInt*^) model of intestinal cancer. **b** Kaplan-Meier survival curves of *Villin-Cre;Apc*^*lox/+*^;*LSL-R26*^*Snail/+*^ (termed *VApc*^*ΔInt*^*;Snail*^*KI/+*^; *n* = 10, median survival 419 days) and *VApc*^*ΔInt*^ mice (*n* = 8, median survival 355 days); *p* = 0.12, log-rank test. **c** qRT-PCR of Snail mRNA expression normalized to Cyclophilin A in intestinal tumours of *VApc*^*ΔInt*^ (*n* = 3) and *VApc*^*ΔInt*^*;Snail*^*KI/+*^ (*n* = 4) endpoint mice (Mean ± SEM; **p = 0.0035, unpaired two-tailed *t*-test with We’ch’s correction). **d** Number of adenomas in *VApc*^*ΔInt*^ (*n* = 8) and *VApc*^*ΔInt*^*;Snail*^*KI/+*^ (*n* = 9) endpoint mice. Mean ± SEM, *p* = 0.08, Mann-Whitney two-tailed test. **e** Percentage of carcinoma-bearing mice (left) and carcinoma number (right) in *VApc*^*ΔInt*^ (*n* = 8) and *VApc*^*ΔInt*^*;Snail*^*KI/+*^ (*n* = 9) endpoint mice. Left, two-tailed Fisher’s exact test, *p* > 0.9999; right, Mann-Whitney two-tailed test, mean ± SEM, *p* = 0.14. **f** Representative H&E and Ki67 staining of invasive intestinal carcinoma of three *VApc*^*ΔInt*^ and *VApc*^*ΔInt*^*;Snail*^*KI/+*^ mice. **g** Strategy to express Snail in intestinal epithelium in the *Villin-Cre;LSL-Kras*^*G12D/+*^ model (termed *VKras*^*G12D/+*^) of intestinal cancer. **h** Kaplan-Meier survival curves of *VKras*^*G12D/+*^ (*n* = 5, median survival 354 days) and *Villin-Cre;Kras*^*G12D/+*^*;LSL-R26*^*Snail/+*^ (termed *VKras*^*G12D/+*^*;Snail*^*KI/+*^) mice (*n* = 5, median survival 502 days); *p* = 0.29, log-rank test. **i** qRT-PCR of Snail mRNA expression in the intestine of *VKras*^*G12D/+*^ (*n* = 5) and *VKras*^*G12D/+*^*;Snail*^*KI/+*^ endpoint mice (*n* = 3). Mean ± SEM, **p = 0.005, unpaired two-tailed *t*-test with Welch’s correction. **j** Number of adenomas in *VKras*^*G12D/+*^ (*n* = 5) and *VKras*^*G12D/+*^*;Snail*^*KI/+*^ (*n* = 5) endpoint mice. Mean ± SEM, Mann-Whitney two-tailed test; *p* = 0.795 (ns, not significant). **k** Representative H&E and Ki67 staining of intestinal adenoma in three *VKras*^*G12D/+*^ and *Vkras*^*G12D/+*^*;Snail*^*KI/+*^ mice. **l** Percentage of carcinoma-bearing mice (left) and carcinoma number (right) in *VKras*^*G12D/+*^ (*n* = 5) and *VKras*^*G12D/+*^*;Snail*^*KI/+*^ (*n* = 5) endpoint mice. Left, two-tailed Fisher’s exact test (*p* > 0.9999); right, two-tailed Student’s *t*-test (*p* = 0.72), mean ± SEM. **m** Representative H&E staining of intestinal carcinoma in two *VKras*^*G12D/+*^ and three *VKras*^*G12D/+*^*;Snail*^*KI/+*^ mice. **n** Pathological grading of intestinal carcinomas from *VApc*^*ΔInt*^ (*n* = 44), *VApc*^*ΔInt*^*;Snail*^*KI/+*^ (*n* = 21), *VKras*^*G12D/+*^ (*n* = 2), *VKras*^*G12D/+*^*;Snail*^*KI/+*^ (*n* = 4), *VBraf*^*V637E/+*^ (*n* = 8) and *VBraf*^*V637E/+*^*;Snail*^*KI/+*^ (*n* = 6) mice; two-tailed Fisher’s exact test. FC, fold change; ns, not significant; scale bars, 50μm for all images. Source data are provided in the Source Data file.

To assess the impact of SNAIL on tumour development, progression and survival of intestinal cancer, where SNAIL is aberrantly expressed in 78% of cases^18^, we used three different genetically engineered models that mirror major histopathological and molecular disease subtypes^19–21^ (Fig. 2 and Supplementary Fig. S2). Surprisingly, in the classical *Apc* loss-of-function model (*VApc*^*ΔInt*^) that is driven by activation of canonical WNT signalling^20^, there was a trend towards prolonged survival (median survival of 419 vs. 355 days; *p* = 0.12) and reduced number of adenomas and carcinomas per animal upon aberrant SNAIL expression (Fig. 2a–e; *p* = 0.08 and 0.14, respectively). SNAIL had no major effect on tumour morphology and histopathology (Fig. 2f). This stands in sharp contrast to the dramatic pro-tumorigenic effects observed in the KRAS-driven PDAC model and suggests that SNAIL has context-specific functions in different tumour entities and/or oncogenic backgrounds. To test the hypothesis that SNAIL cooperates specifically with KRAS, but not WNT pathway activation, to induce cancer progression across tumour entities, we activated SNAIL in vivo in a KRAS^G12D^-driven model of serrated intestinal cancer (Fig. 2g). This specific CRC subtype is characterized by a serrated histopathological morphology and progresses through a hyperplasia - serrated adenoma - serrated carcinoma sequence distinct from the classical WNT-driven CRC progression model described by Vogelstein and colleagues^22^, which is characterized by an adenoma-carcinoma sequence without hyperplasia and serrated morphology^23,24^. Unexpectedly, aberrant SNAIL expression failed to accelerate oncogenesis. Instead, we observed a weak trend towards prolonged survival in the KRAS-driven intestinal cancer model (median survival of 502 days (*VKras*^*G12D/+*^*;Snail*^*KI/+*^) vs 354 days (*VKras*^*G12D/+*^); *p* = 0.29; Fig. 2h, i). Accordingly, we detected no obvious change in the number of adenomas and carcinomas per animal, as well as in the grading of the tumours (Fig. 2j–n). These data indicate that the tissue of origin and not the oncogenic driver dictates the functional role of SNAIL in tumour progression in vivo.

In the Braf^V637E^-driven model of serrated intestinal cancer, we observed more invasive carcinomas in animals with aberrant SNAIL expression (*Villin-Cre;LSL-Braf*^*V637E/+*^;*Snail*^*KI/+*^; termed *VBraf*^*V637E/+*^*;Snail*^*KI/+*^) than in *VBraf*^*V637E/+*^ animals, and also more adenomas per animal (Supplementary Fig. S2a–f). Median survival was reduced to 392 days in the *VBraf*^*V637E/+*^*;Snail*^*KI/+*^ cohort vs. 481 days in *VBraf*^*V637E/+*^*;* mice (Supplementary Fig. S2b; *p* = 0.0321). However, there was no overt consistent change in the grade of carcinomas observed towards more undifferentiated tumours in any of the three intestinal cancer models (Fig. 2f, m, n and Supplementary Fig. S2f).

These observations support the notion that SNAIL acts as a classical cancer-promoting oncogene particularly in KRAS-driven pancreatic and to a far lesser extent in BRAF-driven intestinal cancer, demonstrating its context-specific functions in cancer.

### *Snail* activation fails to repress E-cadherin and does not induce an EMT program in PDAC

SNAIL is a master regulator of EMT that is associated with cancer aggressiveness, metastasis and decreased patient survival in various cancer types, such as PDAC^5,7,14,25,26^. We therefore analysed the effect of *Snail* activation on morphological and transcriptional EMT readouts in our autochthonous cancer models in vivo.

**Fig. 3:**
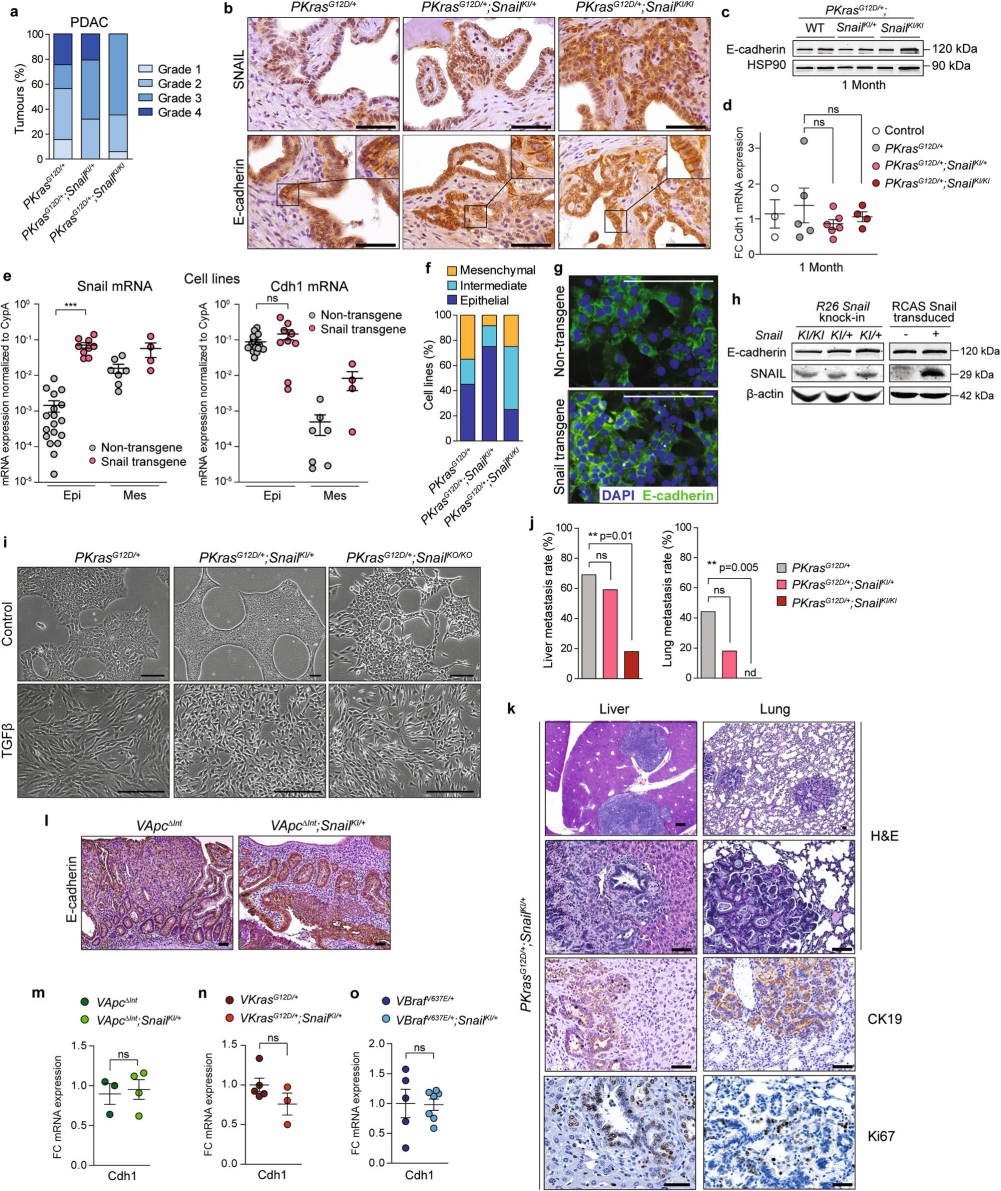
SNAIL does not induce epithelial to mesenchymal transition (EMT) in PDAC. **a** Grading of *PKras*^*G12D/+*^ (*n* = 32), *PKras*^*G12D/+*^*;Snail*^*KI/+*^ (*n* = 19) and *PKras*^*G12D/+*^*;Snail*^*KI/KI*^ PDAC mice (*n* = 17). Grade 4=undifferentiated/sarcomatoid^86^. **b** Representative staining of SNAIL and E-cadherin in PDAC sections of endpoint mice (*n* = 3 per genotype). **c** E-cadherin western blot of pancreas of 1-month-old mice (*n* = 2 per genotype). **d** qRT-PCR of Cdh1 mRNA expression normalized to Cyclophilin A (CypA) in pancreas of 1-month-old mice (Control, *n* = 3; *PKras*^*G12D/+*^ *n* = 5; *PKras*^*G12D/+*^*;Snail*^*KI/+*^ *n* = 6; *PKras*^*G12D/+*^*;Snail*^*KI/KI*^ *n* = 4). Mean ± SEM, unpaired two-tailed *t*-test with Welch’s and Bonferroni correction. **e** qRT-PCR of Snail (left) and Cdh1 (right) mRNA expression of PDAC cells with or without transgenic Snail expression (epi, epithelial (*n* = 27); mes, mesenchymal (*n* = 11)). Each dot represents one PDAC cell line. Mean ± SEM. ***p = 0.0005, unpaired two-tailed *t*-test with Welch’s correction. **f** Percentage of *PKras*^*G12D/+*^ (*n* = 20), *PKras*^*G12D/+*^*;Snail*^*KI/+*^ (*n* = 12) and *PKras*^*G12D/+*^*;Snail*^*KI/KI*^ (*n* = 8) PDAC cell lines with indicated morphology. **g** E-cadherin immunocytochemistry (green) of PDAC cells with or without transgenic Snail expression (*n* = 3 independent experiments). DAPI counterstain (blue). **h** SNAIL and E-cadherin western blot of *PKras*^*G12D/+*^*;Snail*^*KI/+*^ (*n* = 2), *PKras*^*G12D/+*^*;Snail*^*KI/KI*^ (*n* = 1) (left) and Snail-transduced (RCAS-TVA system) PDAC cells (right) (*n* = 1). **i** PDAC cells from *PKras*^*G12D/+*^, *PKras*^*G12D/+*^*;Snail*^*KI/+*^ and floxed *PKras*^*G12D/+*^*;Snail*^*KO/KO*^ knock-out (KO) mice (*n* = 2 per genotype) treated with TGFβ for 72 h. **j** Total liver (left) and lung (right) metastasis rate of endpoint *PKras*^*G12D/+*^ (*n* = 16), *PKras*^*G12D/+*^*;Snail*^*KI/+*^ (*n* = 17) and *PKras*^*G12D/+*^*;Snail*^*KI/KI*^ (*n* = 17) PDAC mice. **p = 0.01 (liver) and *p* = 0.005 (lung), two-tailed Fisher’s exact test with Bonferroni correction. Nd. Not detected. **k** Representative H&E, CK19 and Ki67 staining of liver and lung metastases of three *PKras*^*G12D/+*^*;Snail*^*KI/+*^ mice. **l** Representative E-cadherin staining of intestinal tumours of *VApc*^*ΔInt*^ and *VApc*^*ΔInt*^*;Snail*^*KI/+*^ endpoint mice (*n* = 3 per genotype). **m** qRT-PCR of Cdh1 mRNA expression in intestinal tumours of *VApc*^*ΔInt*^ (*n* = 3) and *VApc*^*ΔInt*^*;Snail*^*KI/+*^ (*n* = 4) endpoint mice. Mean ± SEM, unpaired two-tailed *t*-test with Welch’s correction. **n**–**o** qRT-PCR of Cdh1 mRNA expression in colon samples of (**n**) *VKras*^*G12D/+*^ (*n* = 5) and *VKras*^*G12D/+*^*;Snail*^*KI/+*^ (*n* = 3), and (**o**) *VBraf*^*V637E/+*^ (*n* = 5) and *VBraf*^*V637E/+*^*;Snail*^*KI/+*^ (*n* = 7) mice. Mean ±SEM, unpaired two-tailed *t*-test with Welch’s correction. FC Fold change, ns not significant; scale bars 50 μm. Source data are provided in the Source Data file.

H&E, E-cadherin (Cdh1) and CK19 stainings revealed differentiated and undifferentiated cancers regardless of genotype in our three different PDAC models (Fig. 3a, b and Supplementary Fig. 3a–c). However, histopathological grading indicated a trend towards less undifferentiated/sarcomatoid cancers (Grade 4) in the *PKras*^*G12D/+*^*;Snail*^*KI*^ model in a *Snail* gene dose-dependent fashion (Fig. 3a and Supplementary Fig. S3a–c). Morphologically, *PKras*^*G12D/+*^*;Snail*^*KI/+*^ and *PKras*^*G12D/+*^*;Snail*^*KI/KI*^ mice displayed a phenotype characterized by budding of epithelial tumour cells with the formation of small solid tumour cell nests (Supplementary Fig. S3b, c). These areas retained epithelial differentiation, visible as CK19 and E-cadherin positivity, adjacent to tubular ductal structures (Supplementary Fig. S3b). Aberrant SNAIL expression therefore did not drive PDAC development to a more undifferentiated or sarcomatoid phenotype.

**Fig. 4:**
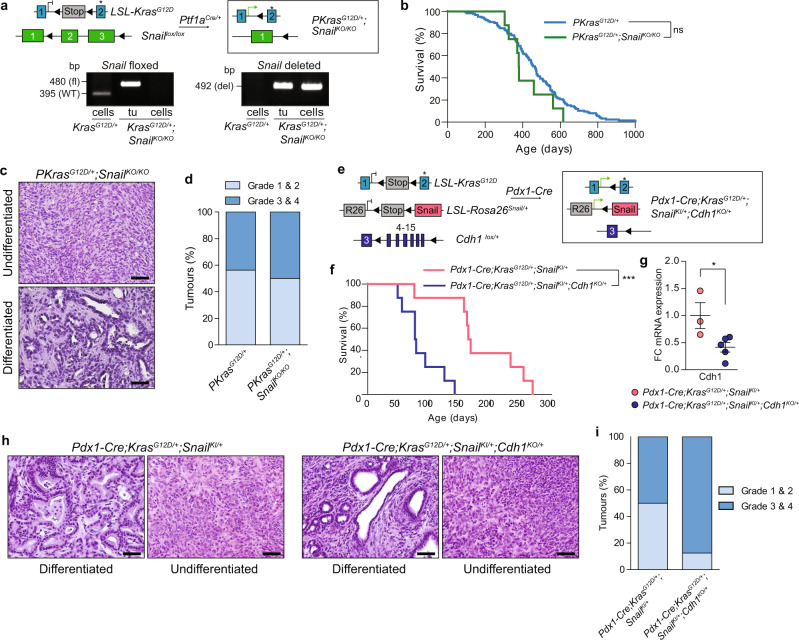
Downregulation of Cdh1 expression drives undifferentiated PDAC formation. **a** Upper panel: Genetic strategy to conditionally delete a floxed *Snail* allele (*Snail*^*lox*^) in the pancreas of *Kras*^*G12D*^ expressing mice. Lower panel: Genotyping PCR to test *Snail*-deletion (*Snail-KO*) using DNA from PDAC cells (cells) and tumour tissue with non-recombined stroma (tu) of *PKras*^*G12D/+*^ and *PKras*^*G12D/+*^*;Snail*^*KO/KO*^ mice (*n* = 2 per genotype). Lower left panel: Floxed *Snail* allele (fl): 480 bp, *Snail* WT allele (WT): 395 bp, deleted *Snail* allele: no band. Lower right panel: deleted *Snail* allele (del): 492 bp; floxed *Snail* and WT allele: no band. **b** Kaplan-Meier survival curves of indicated genotypes of *PKras*^*G12D/+*^*;Snail*^*KO/KO*^ mice (*n* = 8; median survival 380 days), compared to *PKras*^*G12D/+*^ (*n* = 125; median survival 465 days). ns, not significant, log-rank test. **c** Representative H&E-stained PDAC tissue sections of *PKras*^*G12D/+*^*;Snail*^*KO/KO*^ mice with undifferentiated (upper panel) and differentiated (lower panel) morphology (*n* = 6). **d** Pathological grading of PdACs of *PKras*^*G12D/+*^ (*n* = 32) and *PKras*^*G12D/+*^*;Snail*^*KO/KO*^ mice (*n* = 6). **e** Genetic strategy to conditionally delete *Cdh1* and express Snail in the pancreas of *PKras*^*G12D/+*^ mice. **f** Kaplan-Meier survival curves of indicated genotypes of *Pdx1-Cre;Kras*^*G12D/+*^*;Snail*^*KI/+*^*;Cdh1*^*KO/+*^ (*n* = 8; median survival 78 days), compared to *Pdx1-Cre;Kras*^*G12D/+*^*;Snail*^*KI/+*^ mice (*n* = 8; median survival 166 days). ***p = 0.0008, log rank test. **g** qPCR analysis of Cdh1 mRNA expression in PDACs of *Pdx1-Cre;Kras*^*G12D/+*^*;Snail*^*KI/+*^*;Cdh1*^*KO/+*^ (*n* = 5) and *Pdx1-Cre;Kras*^*G12D/+*^*;Snail*^*KI/+*^ (*n* = 3) endpoint mice. Cdh1 mRNA levels were normalized to Cyclophilin A. Mean ± SEM, *p = 0.036, Mann-Whitney two-tailed test. FC, fold change. **h** Representative H&E-stained PDAC tissue sections of indicated *Pdx1-Cre;Kras*^*G12D/+*^*;Snail*^*KI/+*^ and *Pdx1-Cre;Kras*^*G12D/+*^*;Snail*^*KI/+*^*;Cdh1*^*KO/+*^ mice with differentiated and undifferentiated morphology (*n* = 8 per genotype). **i** Pathological grading of PDACs in *Pdx1-Cre;Kras*^*G12D/+*^*;Snail*^*KI/+*^ (*n* = 8) and *Pdx1-Cre;Kras*^*G12D/+*^*;Snail*^*KI/+*^*;Cdh1*^*KO/+*^ mice (*n* = 8). Note: The *PKras*^*G12D/+*^ cohort in (**b**) is the same shown in Figs. 1g, 6e, f. Source data of Fig. 4 are provided in the Source Data file.

SNAIL is known to repress the transmembrane glycoprotein CDH1 (E-cadherin) in several contexts, thereby inducing EMT, migration, invasion and metastasis^7,27^. Surprisingly, expression of SNAIL had no effect on CDH1 expression in vivo, nor in cultured primary PDAC cell isolates in vitro (Fig. 3b–e). E-cadherin protein and mRNA expression levels were comparable in cells with and without transgenic *Snail* expression (Fig. 3b–e). There was a marked decrease in the proportion of mesenchymal, and an increase in the epithelial phenotype in tumour cell lines from the *PKras*^*G12D/+*^*;Snail*^*KI/+*^ model compared to *PKras*^*G12D/+*^ controls (Fig. 3f and Supplementary Fig. S3d). Thus, there was no shift to a mesenchymal phenotype in the *Snail*-transgenic cell lines, not even with biallelic *Snail* expression, although we observed a > 5-fold higher expression of *Snail* in mesenchymal vs. epithelial PDAC cells without transgene (Fig. 3e, f and Supplementary Fig. S3d). Furthermore, global mRNA expression profiles revealed no EMT-related signatures and epithelial cancer cell isolates from *PKras*^*G12D/+*^*;Snail*^*KI*^ transgenic mice clustered exclusively with epithelial PDAC cells from *PKras*^*G12D/+*^-driven models, irrespectively of the *Trp53* mutational status (Supplementary Fig. S3e). To activate SNAIL expression in established epithelial PDAC cells, we transduced them with a retroviral *Snail* expression cassette. E-cadherin localization, expression levels, and cell morphology remained unchanged (Fig. 3g, h, Supplementary Fig. S3f), indicating that SNAIL expression alone is not sufficient to induce an overt EMT program in PDAC cells. To investigate whether SNAIL expression influences the ability of PDAC cells to undergo EMT, epithelial PDAC cells isolated from *PKras*^*G12D/+*^, *PKras*^*G12D/+*^*;Snail*^*KI/+*^ and from conditional pancreas-specific *Snail* knock-out (KO) mice (*PKras*^*G12D/+*^*;Snail*^*KO/KO*^; see also Fig. 4a–d) were treated with the strong EMT-inducer TGFβ. All TGFβ-treated cells, regardless of genotype, underwent rapid EMT and displayed mesenchymal morphology (Fig. 3i), providing genetic evidence that SNAIL is dispensable for EMT-induction.

Beside the unchanged tumour differentiation status, what is also surprising is that aberrant SNAIL expression did not increase metastasis into liver and lung. Indeed, heterozygous transgenic *PKras;Snail*^*KI/+*^ mice had a trend towards reduced metastases, and biallellic *PKras;Snail*^*KI/KI*^ mice significantly fewer (Fig. 3j, k). The capacity of autochthonous tumours to metastasize to liver and lung is thus independent of SNAIL expression, as demonstrated earlier^13^.

Results from the three different models of intestinal cancer were notably similar. Adenomas and carcinomas were mainly well differentiated and showed no histopathological signs or features of EMT induction regardless of SNAIL expression. Consistent with this, E-cadherin expression was retained in SNAIL-expressing adenomas and carcinomas (Fig. 3l–o), indicating that aberrant SNAIL expression is insufficient to drive a full EMT program in the intestinal epithelium.

To validate our findings in genetic loss-of-function models in vivo, we deleted *Snail* in KRAS-driven PDAC using the Cre/loxP system (Fig. 4a). As previously demonstrated^13^, this did not significantly alter PDAC development. *Snail* knock-out animals displayed similar tumour burden and overall survival to control mice (Fig. 4b–d). Importantly, and in line with our previous findings, loss of *Snail* did not drive PDAC to a well-differentiated epithelial phenotype nor block development of undifferentiated cancers that had already undergone an EMT program (Fig. 4c, d). Deletion of *Snail* does not therefore block EMT in vitro or in vivo.

Using a genetic approach to assess E-cadherin/*Cdh1* function in the aberrant *Snail* expression model, we knocked out one floxed allele of the *Cdh1* tumour suppressor in *Pdx1-Cre;Kras*^*G12D/+*^*;Snail*^*KI/+*^ mice (Fig. 4e). E-cadherin mRNA levels were reduced in *Pdx1-Cre;Kras*^*G12D/+*^*;Snail*^*KI/+*^*;Cdh1*^*lox/+*^ animals, which showed dramatically shortened median survival (78 days) compared to *Pdx1-Cre; Kras*^*G12D/+*^*;Snail*^*KI/+*^ (166 days) mice (Fig. 4f, g) and a clear shift of the tumours towards an undifferentiated mesenchymal phenotype (Fig. 4h, i). These data reveal that E-cadherin expression and function is independent of SNAIL in PDAC, and suppresses tumour progression and mesenchymal transition in vivo.

### SNAIL bypasses senescence during pancreatic carcinogenesis

**Fig. 5:**
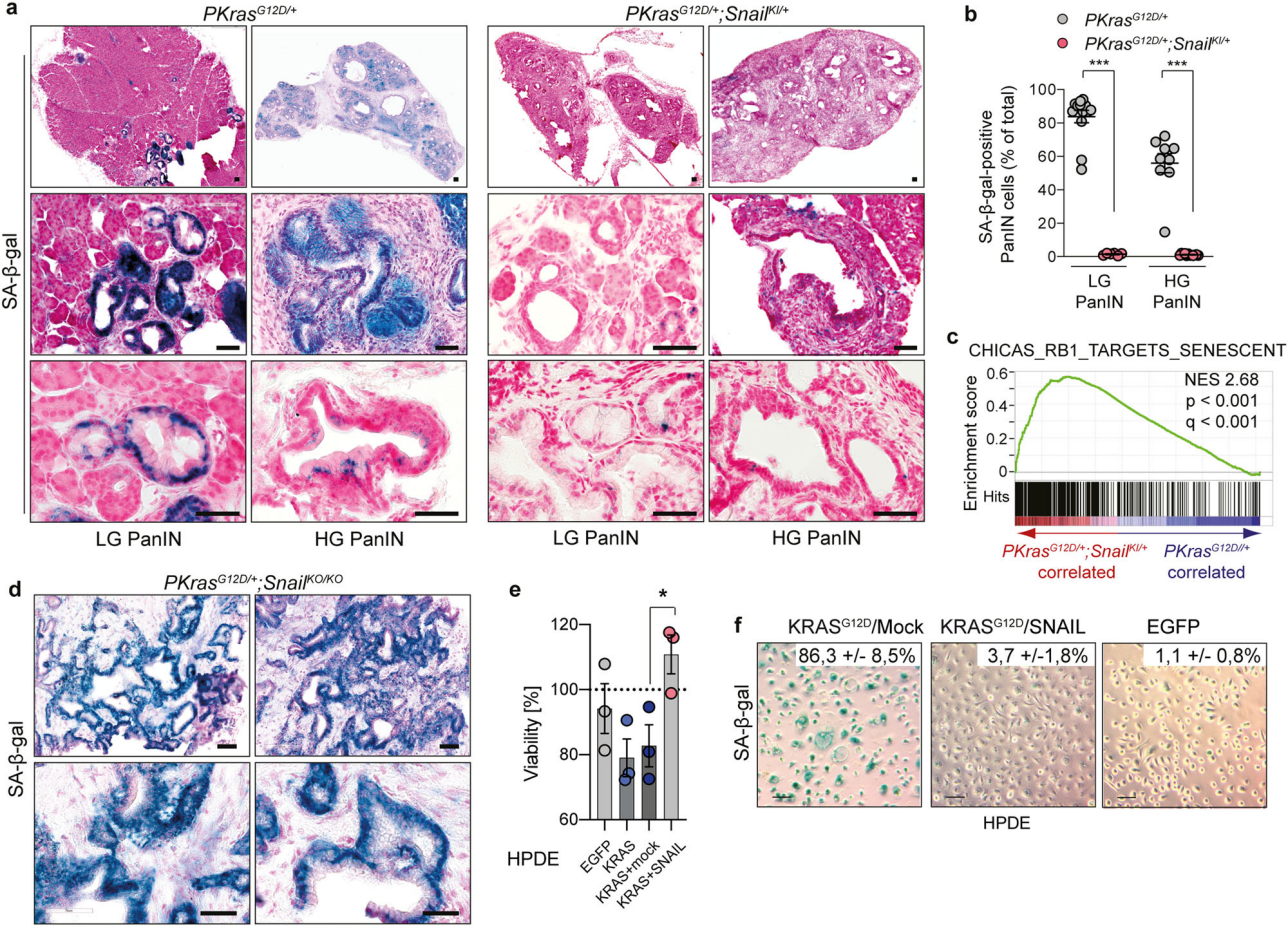
SNAIL bypasses senescence to drive pancreatic carcinogenesis. **a** Representative images of senescence-associated β-galactosidase (SA-β-gal) staining of pancreata with PanIN lesions of *PKras*^*G12D/+*^ (*n* = 13) and *PKras*^*G12D/+*^*;Snail*^*KI/+*^ (*n* = 12) mice. Scale bars, 50 μm. LG, low grade; HG, high grade. **b** Quantification of SA-β-gal-stained PanIN lesions from *PKras*^*G12D/+*^ (*n* = 13) and *PKras*^*G12D/+*^*;Snail*^*KI/+*^ (*n* = 12) mice. Mean ± SEM, *p < 0.0001, Mann-Whitney two-tailed test. LG, low grade; HG, high grade. **c** Gene set enrichment analysis (GSEA) of mRNA expression profiling of 1-month-old mice (*n* = 2 per genotype) computed and corrected for multiple testing using the Benjamini–Hochberg procedure (for statistical details, please see methods section) shows significant enrichment of Rb1 targets senescent genes (CHICAS_RB1_TARGETS_SENESCENT) in *PKras*^*G12D/+*^*;Snail*^*KI/+*^ (red) vs. *PKras*^*G12D/+*^ (blue) pancreata. Normalized Enrichment Score: 2.68; Nominal *p*-value < 0.001; False Discovery Rate (FDR) *q*-value < 0.001. **d** Representative images of SA-β-gal staining of pancreata with PanIN lesions of *PKras*^*G12D/+*^*;Snail*^*KO/KO*^ mice (*n* = 2). Scale bars, 50 μm. LG Low grade, HG High grade. **e** Viability of Human Pancreatic Duct Epithelial (HPDE) cells after activation of *KRAS*^*G12D*^ alone or in combination with *SNAIL*. HPDE cells transduced with lentiviral constructs for doxycycline-inducible expression of EGFP, *KRAS*^*G12D*^ ( + mock vector) or *KRAS*^*G12D*^ + *SNAIL* were treated with 100 ng ml^-1^ doxycycline. Viability was assessed by CellTiter-Glo assay after 72 h and is displayed as % of the respective untreated controls. Mean ± SEM. *n* = 3 independent experiments; *p = 0.033, unpaired two-tailed *t*-test with Welch’s correction. **f** Representative images of SA-β-Gal staining of HPDE cells treated for 3 days with doxycycline (100 ng ml^-1^) to induce activation of EGFP, *KRAS*^*G12D*^ + mock or *KRAS*^*G12D*^ + *SNAIL*. *n* = 3 independent experiments. The percentage of SA-β-gal^+^ cells is indicated in the upper right corner. Scale bar, 10 µm. Source data of Fig. 5 are provided in the Source Data file.

To discern how aberrant SNAIL expression promotes rapid tumour progression in the *PKras*^*G12D/+*^*;Snail*^*KI/+*^ PDAC model, independent of overt EMT induction, we investigated early tumour barriers and events in tumour formation. Oncogene-induced senescence (OIS), a feature of KRAS-driven premalignant PanIN lesions of the pancreas^28,29^, is a cellular stress response, which blocks proliferation so protecting cells from neoplastic transformation^30^. As reported previously, nearly all PanINs of the *PKras*^*G12D/+*^ model displayed positive senescence-associated β-galactosidase (SA-β-gal) staining, the most reliable marker of OIS in the pancreas^31^. In contrast, we observed a dramatically reduced rate of OIS in *PKras*^*G12D/+*^*;Snail*^*KI/+*^ mice (Fig. 5a, b), which displayed an almost complete loss of OIS in premalignant PanIN lesions (Fig. 5b). In addition, we observed loss of senescence-associated gene sets controlled by the retinoblastoma (RB) tumour suppressor gene in expression profiles of PanIN bearing *PKras*^*G12D/+*^*;Snail*^*KI/+*^ pancreata (Fig. 5c). In contrast, deletion of *Snail* in the *PKras*^*G12D/+*^ model (*PKras*^*G12D/+*^*;Snail*^*KO/KO*^) induced a strong senescence phenotype as evidenced by SA-β-gal staining of PanIN bearing pancreatic tissue sections (Fig. 5d), indicating that SNAIL is indeed capable of impacting OIS.

To probe SNAIL function in a human model and validate the relevance of our findings for human PDAC development, we employed immortalized human pancreatic duct epithelial (HPDE) lineage cells and engineered them with doxycycline-inducible human KRAS^G12D^ and SNAIL expression vectors (Fig. 5e, f and Supplementary Fig. S4a). Activation of oncogenic KRAS^G12D^ in HPDE cells induced strong morphological changes as well as OIS, as demonstrated by SA-β-gal staining, accompanied by a decrease in cell viability compared to EGFP transduced controls. In contrast, co-expression of KRAS^G12D^ and SNAIL reverted this phenotype almost completely, resulting in a loss of OIS and increased HPDE proliferation (Fig. 5e, f, Supplementary Fig. S4a). PDAC cells isolated from full blown tumours displayed no SA-β-gal staining and senescence phenotype, indicating that OIS is bypassed during early steps of pancreatic carcinogenesis as described previously^28^ (Supplementary Fig. S4b). Thus, we demonstrate in genetic mouse and engineered human pancreatic epithelial lineage cells that SNAIL expression bypasses OIS, thereby driving pancreatic cancer initiation.

### SNAIL overcomes senescence without inactivating the TRP53/p21^CIP1^ axis

**Fig. 6:**
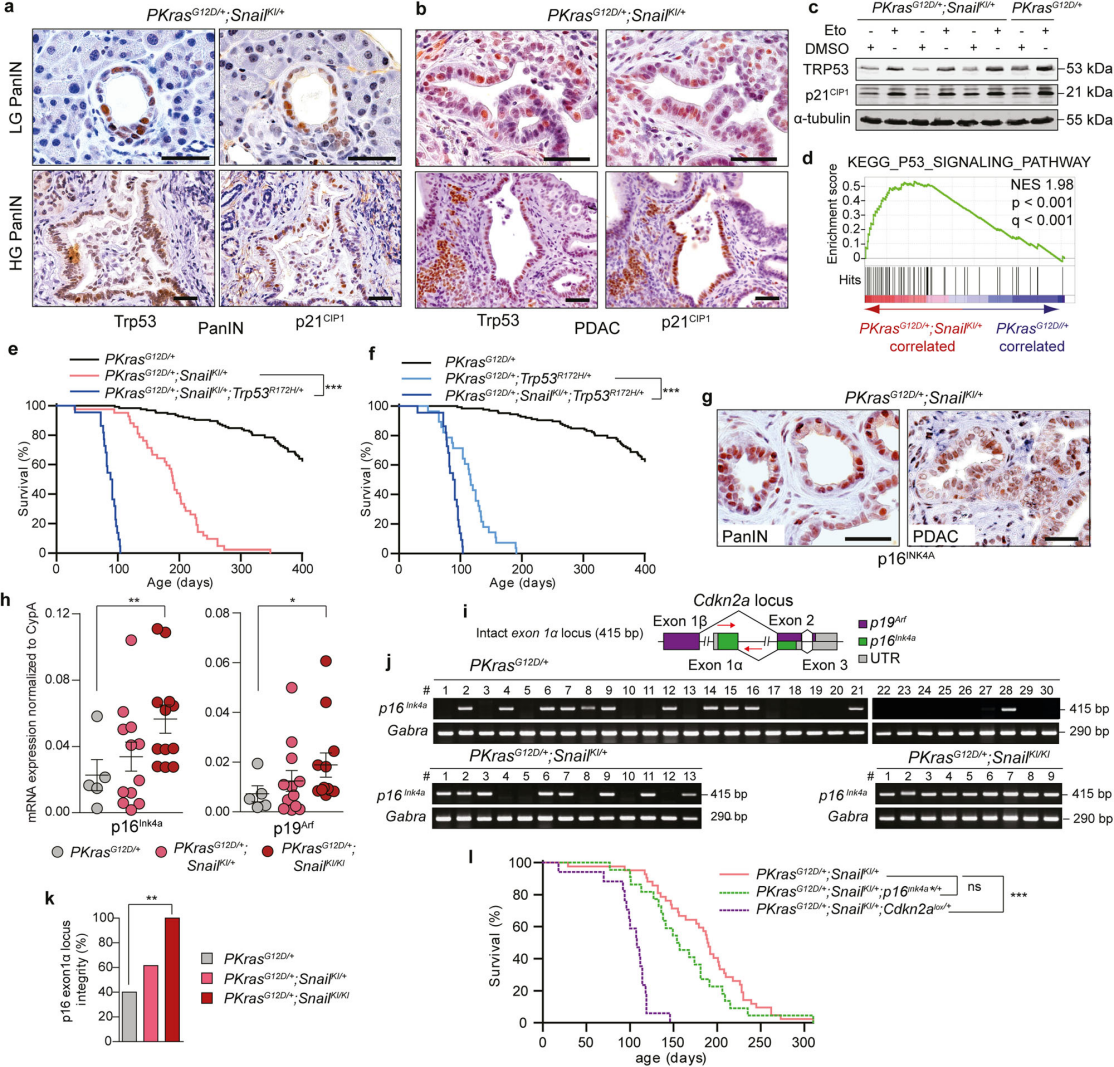
SNAIL overcomes senescence and the p16^*INK4A*^ cell cycle restriction checkpoint without altering the Trp53/p21^CIP1^ axis. **a**, **b** Representative Trp53 and p21^CIP1^ stainings of PanINs (**a**) and PDAC (**b**) of *PKras*^*G12D/+*^*;Snail*^*KI/+*^ animals (*n* = 3 each). **c** Western blot of TRP53 and p21^CIP1^ expression in *PKras*^*G12D/+*^*;Snail*^*Ki/+*^ (*n* = 3) and *PKras*^*G12D/+*^ (*n* = 1) PDAC cell lines after 6 h 20 μM etoposide (Eto) or vehicle (DMSO) treatment. **d** GSEA of mRNA expression of 1-month-old mice (*n* = 2 per genotype) computed and corrected for multiple testing using the Benjamini–Hochberg procedure (for statistical details, see methods section) shows significant enrichment of KEGG p53 signalling pathway genes in *PKras*^*G12D/+*^*;Snail*^*KI/+*^ (red) vs. *PKras*^*G12D/+*^ (blue). Normalized Enrichment Score: 1.98; Nominal *p*-value < 0.001; False Discovery Rate (FDR) *q*-value < 0.001. **e**, **f** Kaplan-Meier survival curves of *PKras*^*G12D/+*^ (*n* = 125; 465 days), *PKras*^*G12D/+*^*;Snail*^*KI/+*^ (*n* = 42; 190 days), *PKras*^*G12D/+*^*;Trp53*^*R172H/+*^ (*n* = 28; 117 days) and *PKras*^*G12D/+*^*;Snail*^*KI/+*^*;Trp53*^*R172H/+*^ (*n* = 22; 90 days) animals. ***p < 0.0001, log-rank test with Bonferroni correction. **g** Representative p16^INK4A^ staining of PanINs (left) and PDAC (right) of *PKras*^*G12D/+*^*;Snail*^*KI/+*^ mice (*n* = 3 each). **h** qRT-PCR analysis of p16^Ink4a^ (left) and p19^Arf^ (right) mRNA expression in PDAC of endpoint mice (*PKras*^*G12D/+*^ *n* = 5; *PKras*^*G12D/+*^*;Snail*^*KI/+*^ *n* = 12; *PKras*^*G12D/+*^*;Snail*^*KI/KI*^ *n* = 12). Mean ± SEM, **p = 0.0094, *p = 0.0365, Mann-Whitney two-tailed test. **i** Scheme of *Cdkn2a* gene locus and *p16*^*Ink4a*^ genotyping strategy. The non-related proteins p16^INK4A^ and p19^ARF^ are encoded both by the *Cdkn2a* locus. Red arrows, primer positions. UTR, untranslated region. Scheme according to^35^. **j** PCR of *p16*^*Ink4a*^ genomic sequence integrity in PDAC cell lines of *PKras*^*G12D/+*^ (*n* = 30), *PKras*^*G12D/+*^*;Snail*^*KI/+*^ (*n* = 13); *PKras*^*G12D/+*^*;Snail*^*KI/KI*^ (*n* = 9) endpoint mice. Gabra, internal positive control. **k** Quantification of PCR analysis of *p16*^*Ink4a*^ genomic sequence integrity of data in panel (**j**). **p = 0.0016, two-tailed Fisher’s exact test. **l** Kaplan-Meier survival curves of *PKras*^*G12D/+*^*;Snail*^*KI/+*^ (*n* = 42; 190 days), *PKras*^*G12D/+*^*;Snail*^*KI/+*^*;p16*^Ink4a*/+^ (*n* = 22; 156 days) and *PKras*^*G12D/+*^*;Snail*^*KI/+*^*;Cdkn2a*^*lox/+*^ with loss of p16^INK4A^ and p19^ARF^ (*n* = 17; 108 days). ***p < 0.0001, log-rank test with Bonferroni correction. Note: *PKras*^*G12D/+*^ cohort in panel (**e**, **f**) is the same shown in Figs. 1g, 4b, and *PKras*^*G12D/+*^*;Snail*^*KI/+*^ cohort of panel **e** and **l** is the same shown in Fig. 1g. Source data of Fig. 6 are provided in the Source Data file. Scale bars, 50 μm. ns, not significant.

Oncogene-induced senescence is associated with induction of the tumour suppressor TRP53 and/or p16^INK4A^ depending on cellular context and their loss facilitates tumor formation (Supplementary Fig. S4c)^30,32^. As shown by immunohistochemistry, TRP53 and its functional downstream target p21^CIP1^ are expressed in premalignant PanIN lesions and full-blown PDAC cells of *PKras*^*G12D/+*^*;Snail*^*KI/+*^ mice in vivo (Fig. 6a, b). Because mutant gain- or loss-of-function TRP53 is unable to transcriptionally activate p21^CIP1^ ^28^, these data support the notion that i) expression and function of the TRP53/p21^CIP1^ axis is intact in our model, and ii) the observed bypass of senescence in *PKras*^*G12D/+*^*;Snail*^*KI/+*^ pancreata is independent of the TRP53 pathway. To further substantiate these findings, we show upregulation of TRP53 and p21^CIP1^ protein abundance in full-blown *PKras*^*G12D/+*^*;Snail*^*KI/+*^ PDAC cells upon treatment with the topoisomerase inhibitor etoposide in vitro (Fig. 6c) and enrichment of TRP53 downstream targets in pancreatic gene expression profiles of PanIN-bearing *PKras*^*G12D/+*^*;Snail*^*KI/+*^ mice (Fig. 6d).

To test the functional relevance of the TRP53 pathway in an in vivo model, we used a genetically engineered *p53* mutant allele, which lacks canonical TRP53 function and p21^CIP1^ induction^33^. These animals harbour the murine orthologue of the Li-Fraumeni hotspot mutation *R175H* at the endogenous murine *Trp53* locus (*Trp53*^*R172H*^). In this p53 mutant background, *PKras*^*G12D/+*^*;Snail*^*KI/+*^*;Trp53*^*R172H/+*^ mice displayed a dramatically accelerated PDAC formation and showed in vivo evidence for synergy between aberrant SNAIL expression and functional *p53* inactivation. All animals in the tumour watch cohort developed invasive PDAC. Median survival of *PKras*^*G12D/+*^*;Snail*^*KI/+*^*;Trp53*^*R172H/+*^ mice (90 days) was significantly less than *PKras*^*G12D/+*^*;Snail*^*KI/+*^ (190 days) and *PKras*^*G12D/+*^*;Trp53*^*R172H/+*^ (117 days) mice (Fig. 6e, f). These data demonstrate that TRP53 and SNAIL function, at least in part, via non-overlapping and non-redundant pathways and tumour barriers.

The cell cycle regulator p16^INK4A^, which blocks G1 to S phase progression of the cell cycle, is implicated in OIS and tumour suppression and is frequently lost during KRAS-driven pancreatic carcinogenesis (Fig. 6g–k and Supplementary Fig. S4c)^32^. It is encoded by the *Cdkn2a* locus together with p19^ARF^, which is involved in TRP53 activation by inhibiting Mdm2^34^. Immunohistochemistry revealed strong expression of p16^INK4A^ in PanIN lesions and full-blown PDAC of *PKras*^*G12D/+*^*;Snail*^*KI/+*^ mice (Fig. 6g), indicating intact p16^INK4A^ function. Furthermore, p16^Ink4a^/p19^Arf^ mRNA expression in PDAC tissue was greater in *PKras*^*G12D/+*^*;Snail*^*KI/+*^ than *PKras*^*G12D/+*^ animals, and increased further in *PKras*^*G12D/+*^*;Snail*^*KI/KI*^ animals (Fig. 6h). We therefore tested the integrity of the genomic sequence of the *p16*^*Ink4a*^ locus in cell lines isolated from the three different models (Fig. 6i, j). We observed loss of *p16*^*Ink4a*^ in 60% of *PKras*^*G12D/+*^ PDAC cell lines tested. In contrast, only 38,5% of *PKras*^*G12D/+*^*;Snail*^*KI/+*^ and none of the *PKras*^*G12D/+*^*;Snail*^*KI/KI*^ cell lines were deficient for *p16*^*Ink4a*^ (Fig. 6j, k). These findings suggest that Snail might inactivate the p16-RB controlled cell cycle/OIS restriction checkpoint downstream of *p16*^*Ink4a*^, to block OIS and drive PDAC progression.

To test this hypothesis, we again used genetic in vivo models of pancreatic tumour evolution. We crossed *PKras*^*G12D/+*^ and *PKras*^*G12D/+*^*;Snail*^*KI/+*^ animals with either *p16*^Ink4a*^ mutant loss of function mice^35^, or *Cdkn2a*^*lox*^ mice with conditional knock-out of both p16^Ink4a^ and p19^Arf^ gene products^36^. All animals of the tumour watch cohort developed invasive PDAC. However and as hypothesized, *PKras*^*G12D/+*^*;Snail*^*KI/+*^*;p16*^Ink4a*/+^ mice showed no statistically significant difference in median survival compared to *PKras*^*G12D/+*^*;Snail*^*KI/+*^ mice (Fig. 6l). These data demonstrate at the level of genetics that p16^Ink4a^ and SNAIL function via overlapping pathways/shared tumour barriers. In line, *PKras*^*G12D/+*^*;Snail*^*KI/+*^*;Cdkn2a*^*lox/+*^ mice with loss of both, the p16^Ink4a^ and the p19^ARF^ tumour suppressor barriers, had dramatically reduced median survival (Fig. 6l), confirming our data obtained with the *Trp53*^*R172H*^ mutant in vivo model (Fig. 6e, f).

Taken together, our mechanistic dissection of three important tumor suppressor genes of KRAS-driven carcinogenesis provides strong in vivo genetic evidence that SNAIL bypasses senescence and drives tumour development independent of TRP53 inactivation and downstream of the p16^Ink4a^ cell cycle restriction check-point, e.g., via blocking the RB-controlled senescence-pathway.

**Fig. 7:**
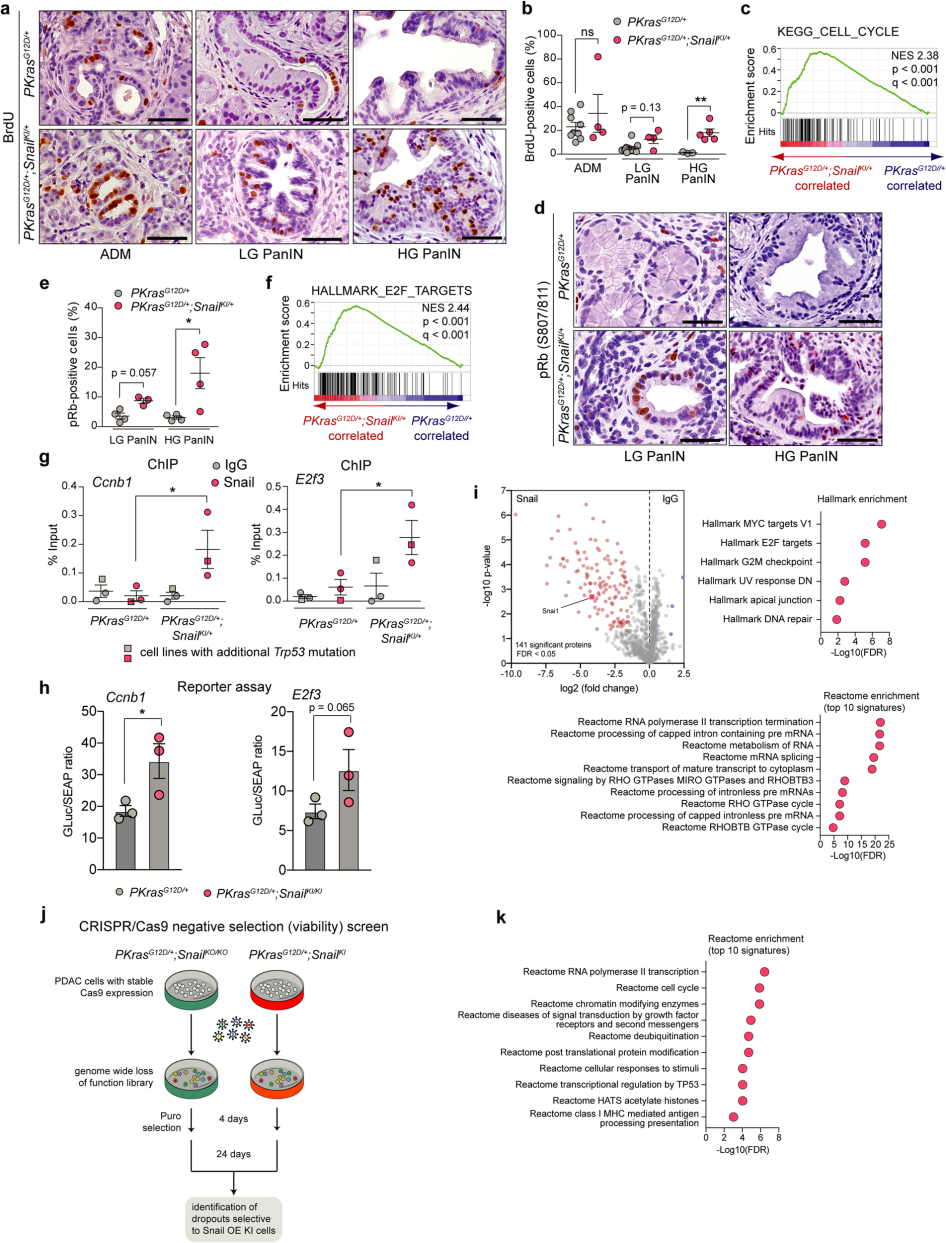
SNAIL drives tumour progression downstream of p16^Ink4A^ by direct activation of cell cycle regulators. **a** Representative BrdU stainings of ADMs and PanINs of *PKras*^*G12D/+*^ (*n* = 11) and *PKras*^*G12D/+*^*;Snail*^*KI/+*^ (*n* = 5) mice. **b** Percentage of BrdU positive cells in ADMs/PanINs of *PKras*^*G12D/+*^*;Snail*^*KI/+*^ (*n* = 5) and *PKras*^*G12D/+*^ (*n* = 11) mice. Mean ± SEM, **p = 0.006, unpaired two-tailed *t*-test with Welch’s correction. **c** GSEA 1-month-old mice (*n* = 2 per genotype) computed and corrected for multiple testing using Benjamini–Hochberg procedure (statistical details see methods) shows significant enrichment of KEGG cell-cycle genes in *PKras*^*G12D/+*^*;Snail*^*Ki/+*^ (red) vs. *PKras*^*G12D/+*^ (blue) pancreata. Normalized Enrichment Score (NES): 2.38; Nominal *p*-value < 0.001; False Discovery Rate (FDR) *q*-value < 0.001. **d**, **e** Representative stainings (**d**) and quantification of pRb-S807/811 positive PanINs (**e**) of *PKras*^*G12D/+*^*;Snail*^*KI/+*^ and *PKras*^*G12D/+*^ animals (*n* = 4 per genotype). Mean ± SEM, *p = 0.029, Mann-Whitney two-tailed test. **f** GSEA corrected for multiple testing using Benjamini–Hochberg procedure shows significant enrichment of hallmark E2F target genes in *PKras*^*G12D/+*^*;Snail*^*KI/+*^ (red) vs. *PKras*^*G12D/+*^ (blue) in 1-month-old mice (*n* = 2 per genotype). NES: 2.44; Nominal *p*-value < 0.001; FDRq-value < 0.001. **g** Chromatin-immunoprecipitation (ChIP) of SNAIL binding to E-boxes of indicated promoters in *PKras*^*G12D/+*^ (*n* = 3) and *PKras*^*G12D/+*^*;Snail*^*KI/+*^ (*n* = 3) PDAC cell lines ± *Trp53* mutation as indicated. %input calculation; IgG, negative control. Mean ± SEM. *p = 0.05, Mann-Whitney one-tailed test. **h** *Ccnb1* and *E2f3* promoter activity in *PKras*^*G12D/+*^ (*n* = 3) and *PKras*^*G12D/+*^*;Snail*^*KI/KI*^ (*n* = 3) PDAC cells (three independent experiments). Mean ± SEM, *p = 0.026, unpaired one-tailed Student’s *t*-test. **i** Volcano-plot representing enriched proteins in *PKras*^*G12D/+*^*;Snail*^*KI/+*^ PDAC cells upon Snail or IgG ChIP, respectively, followed by mass-spectrometry based quantification of co-precipitated proteins (two independent experiments in triplicate for each condition). x-axis, log2-fold change; y-axis, adjusted *p*-value of the two-sample *t*-test (two-tailed, FDR < 0.05, s0 = 1). 141 of 1039 proteins were significant vs. IgG control. Pathway-enrichment analysis of significant proteins with MSigDB Hallmarks (upper right panel) and Reactome (lower panel) databases. **j** Scheme of genome-scale CRISPR/Cas9 negative-selection screen (*PKras*^*G12D/+*^*;Snail*^*KO/KO*^*, PKras*^*G12D/+*^*;Snail*^*KI/+*^*, PKras*^*G12D/+*^*;Snail*^*KI/KI*^ cells; *n* = 4). **k** Differences in β-scores (*Snail*^*KI*^ overexpression (OE) - *Snail*^*KO*^ knock-out (KO) cells) were used for Reactome database enrichment analysis (FDR ≤ 0.05; difference in β score < −1). Scale bars, 50 μm. ns not significant. LG low grade; HG high grade. Source data of Fig. 7 provided in Source Data file.

Consistent with this hypothesis, we observed increased proliferation of premalignant PanIN lesions, as evidenced by BrdU labelling and Ki67 staining (Fig. 7a, b and Supplementary Fig. S4d). In addition, we demonstrate the enrichment of genes that promote cell cycle progression by gene expression profiling of PanIN bearing *PKras*^*G12D/+*^*;Snail*^*KI/+*^ vs. *PKras*^*G12D/+*^ pancreata of one-month-old mice (Fig. 7c), as well as increased DNA damage and apoptosis induction by aberrant SNAIL expression (Supplementary Fig. S4e–i). It has been previously shown that E2F activation in response to RB inactivation leads to p53-dependent apoptosis^37–41^. Thus, combining p53 inactivation with aberrant SNAIL expression accelerates tumorigenesis most likely due to the prevention of p53-dependent apoptosis.

Phosphorylation and thereby inactivation of the tumour suppressor RB by cyclin dependent kinase (CDK)/cyclin complexes, which is negatively regulated by p16^Ink4a^, is an essential step to bypass the firm G1-phase arrest of senescent cells and initiate cell cycle activation and proliferation^42^. RB inactivation leads to dissociation of the E2F complex thereby activating the expression of E2F target genes, which can then drive cell cycle progression as well as apoptosis, as observed in our model^40,41,43^. In line, we detected phosphorylation of RB and thus inactivation of the RB-controlled cell cycle/senescence checkpoint (Fig. 7d, e) in PanIN lesions together with concomitant enrichment of E2F target genes (Fig. 7f) in the *PKras*^*G12D/+*^*;Snail*^*KI/+*^ model. Although gene expression profiling of bulk tissues is confounded by the increased number of PanIN lesions in *PKras*^*G12D/+*^*;Snail*^*KI/+*^ mice at an age of one month, and gene sets that promote cell cycle progression overlap substantially with E2F target genes, our various different in vivo models and datasets provide strong evidence for the hypothesis that SNAIL might bypass senescence downstream of p16^Ink4a^ via interference with the RB-controlled cell cycle/senescence restriction check-point.

### SNAIL is a transcriptional regulator of the cell cycle

Cyclins, such as cyclin A1 (*CCNA1*) or cyclin B1 (*CCNB1*) interact with cyclin-dependent kinases (CDK) to phosphorylate and thereby inactivate RB, which releases E2F transcription factors to enter the nucleus and activate transcription of target genes essential for the transition from G1 to S phase and progression of the cell cycle. Because our data provide genetic evidence that SNAIL bypasses senescence and drives the cell cycle in vivo, we validated the upregulation of important cell cycle regulators and downstream effectors. Transcriptomic profiling and qRT-PCR analysis revealed a marked increase in mRNA expression of several cell cycle-related genes, including cyclins and cyclin-dependent kinases, in pancreata with aberrant SNAIL expression compared with *PKras*^*G12D/+*^ controls (Supplementary Fig. S5a, b), in line with the proliferation and E2F signature shown in Fig. 7c, f.

SNAIL, as a transcription factor, functions primarily via binding to promoter and enhancer regions of the target genes. To test whether SNAIL binds to promoter regions of genes that positively regulate the cell cycle, we analysed publicly available chromatin immunoprecipitation (ChIP)-seq cancer cell line datasets^11^ and compared them to significantly enriched genes in pancreas of one-month-old *PKras*^*G12D/+*^*;Snail*^*KI/+*^ mice. Of 69 enriched genes implicated in proliferation and cell cycle progression, 62 were bound by SNAIL in their promoter region (Supplementary Fig. S5c). Calculation of the odds ratio for this enrichment (8.53; Supplementary Fig. S5c) strongly suggested that the presence of the vast majority (89.9%) of genes in the SNAIL-bound fraction was not due to chance. Thus, SNAIL has a clear preference for binding to genes that promote cell cycle progression. To validate these findings functionally in the *PKras*^*G12D/+*^*;Snail*^*KI/+*^ PDAC model, we performed ChIP experiments using cell lines isolated from *PKras*^*G12D/+*^ and *PKras*^*G12D/+*^*;Snail*^*KI/+*^ mice with and without *Trp53* mutation, and selected SNAIL targets from the ChIP-seq study. This revealed binding of SNAIL to E-boxes in promoter regions of multiple genes, such as *Ccnb1*, *Ccnb2*, *Ccnd1*, *E2f2* and *E2f3* (Fig. 7g and Supplementary Fig. S5d), all known to drive the cell cycle and bound by SNAIL in the published ChIP-seq data (Supplementary Fig. S5c). In line with the ChIP-seq data set, we did not observe binding of SNAIL to the *Ccna1* promoter region, even though this cyclin mRNA is overexpressed in the *PKras*^*G12D/+*^*;Snail*^*KI/+*^ model (Supplementary Fig. S5a). To test whether SNAIL is indeed capable of activating the expression of the identified cell cycle regulators, we performed promoter reporter assays using again our primary PDAC cell cultures. While aberrant SNAIL expression did not increase reporter gene activity of cyclin D1 (*Ccnd1*) and *E2f2* promoter reporter constructs, cyclin B1 (*Ccnb1*; *p* = 0.026), cyclin B2 (*Ccnb2*; *p* = 0.0423) and E2f3 (*p* = 0.065) demonstrated evidence of gene activation (Fig. 7h and Supplementary Fig. S5e).

These findings support a context-specific function of SNAIL in vivo, which bypasses senescence by direct binding and activating important positive regulators of the cell cycle. To gain more insights into SNAIL-mediated gene activation, we studied potential co-regulators from chromatin cross-linked to SNAIL by proteomics and performed chromatin immunoprecipitation coupled to mass spectrometry (ChIP-MS). This allowed us to identify 141 significantly enriched putative chromatin-bound partners of SNAIL (Fig. 7i and Supplementary Data 1). Activating transcription factors, such as NFKB2 and SMAD2, nuclear receptors and coactivators, chromatin remodelers and histone modifiers, such as KDM1A were enriched together with SNAIL (Fig. 7i and Supplementary Data 1). Subsequent pathway analysis of significantly enriched genes revealed regulators of the cell cycle, such as MYC and E2F targets, and genes involved in progression through the G2M checkpoint (Fig. 7i). Further, several genes, e.g., RNA-binding proteins, involved in RNA pol II transcription and transcription termination, RNA metabolism, processing, splicing and transport were significantly enriched together with SNAIL, indicating a role of SNAIL in alternative splicing and RNA biology, which might contribute to its function in regulating cell cycle progression. Of note, NFKB/E2F interactions have previously been shown to control the timing of cell proliferation^44^ and SMAD2 silencing decreased PDAC cell division^45^. In addition, KDM1A has been recently linked to gene activation^46^ and PDAC cell cycle progression^47^. These data suggest that multiple interactions of SNAIL might contribute to its context-dependent role as a transcriptional activator and regulator of the cell cycle.

To identify functional relevant targets of SNAIL driving PDAC progression and maintenance, we performed pooled genome-wide CRISPR/Cas9 loss-of-function (viability) screens with cell lines isolated from *PKras*^*G12D/+*^*;Snail*^*KI*^ mice with aberrant SNAIL expression compared to PDAC cells from SNAIL-deficient *PKras*^*G12D/+*^*;Snail*^*KO/KO*^ animals (Fig. 7j, k, Supplementary Fig. S6 and Supplementary Data 2). We determined differential sensitivity scores^48^ by calculating the difference in β-score between SNAIL overexpressing and deficient cells and further analysed genes displaying a negative differential sensitivity score, pointing to enhanced depletion in *PKras*^*G12D/+*^*;Snail*^*KI*^ cells. This allowed us to identify 238 statistically significant genes, whose depletion led to the specific drop-out of cells with aberrant SNAIL expression.

Pathway analysis of these hits enabled us to uncover the specific genetic dependencies and vulnerabilities of cells with aberrant SNAIL expression, such as cell cycle and checkpoint regulation, E2F targets, NF-kB signaling, RNA pol II transcription and chromatin modifications (Fig. 7k and Supplementary Fig. S6b, c). Importantly, we observed that these pathways and processes correlated to a high degree with the ChIP-MS analysis of Fig. 7i, thereby cross-validating our findings by functional genetic screens. To probe the contribution of the differentially expressed cell cycle regulators for cell viability of SNAIL-driven PDAC, we correlated the β-scores of the *PKras*^*G12D/+*^*;Snail*^*KI*^ cells with their gene expression levels. As shown in Supplementary Fig. S6d, 32 out of a total of the 49 differentially expressed cell cycle-related genes of Supplementary Fig. S5 displayed a significant differential β-score indicating selective depletion in cells with aberrant SNAIL expression.

### Aberrant SNAIL expression is prognostic in human PDAC

**Fig. 8:**
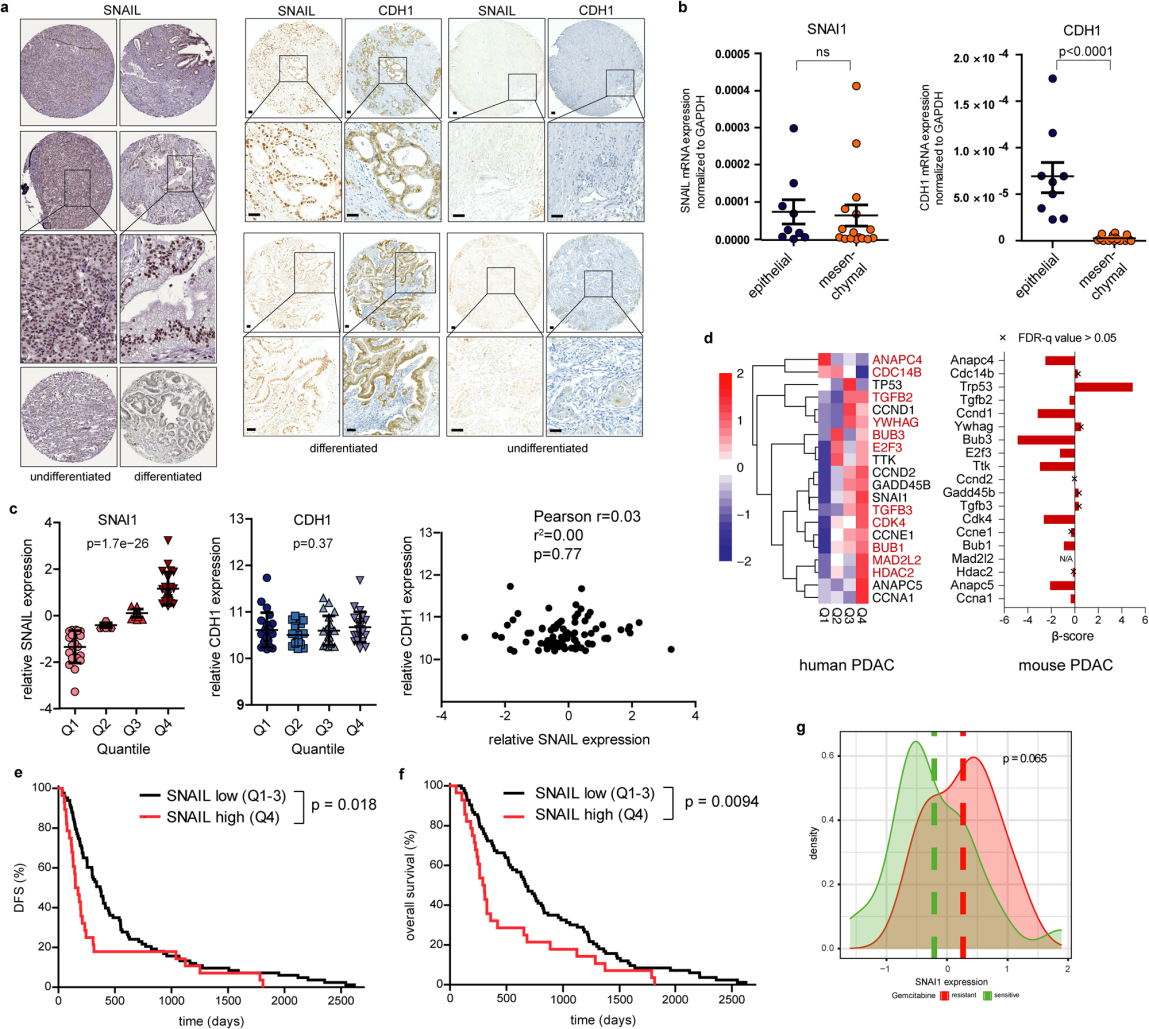
SNAIL expression in human PDAC is independent of EMT and associated with poorer survival and chemoresistance. **a** Left: Representative staining of SNAIL in human PDAC sections of differentiated (G1/2) and undifferentiated tumours (G3/4) of the Human Protein Atlas version 20.1 (http://www.proteinatlas.org)^85^. Images and clinical data are available from https://www.proteinatlas.org/ENSG00000124216-SNAI1/pathology/pancreatic+cancer#ihc. Right: Representative SNAIL and CDH1 staining in serial sections of an independent PDAC patient cohort with G1/2 and G3/4 tumours (*n* = 11). Scale bars, 50 µm. **b** qRT-PCR of SNAIL (SNAI1; left) and E-cadherin (CDH1; right) mRNA expression of human PDAC cell lines (epithelial, *n* = 9; mesenchymal, *n* = 16). Mean ± SEM. Left: ns, not significant (*p* = 0.835) unpaired two-tailed *t*-test; Right: *p* < 0.0001, Mann-Whitney two-tailed test. **c** SNAI1 (left) and CDH1 (middle) expression across SNAI1 quartile group of resected primary human PDAC samples (Q1 to Q4, *n* = 88). Mean ± SEM. *p* = 1.7e-26 (SNAI1) and 0.37 (CDH1), one-way ANOVA-test. Right: Pearson correlation of SNAIL and CDH1 expression across all PDAC samples (*n* = 88). Two-tailed Pearson correlation coefficient *r* = 0.031, r^2^ = 0.0009949, *p* = 0.7705 (not significant), 95% confidence interval −0.1791 to 0.2395. **d** Left: Heatmap of top 20 significant cell cycle related genes with the highest variance across SNAI1 quartile groups. Colour code, row-wise scaling of RNA expression. Row clustered using hierarchical clustering on Euclidean distance. Note: 11 out of the 20 human genes overlap with the cell cycle related genes of Supplementary Fig. S5b identified in the murine model (depicted in red). Right: Cross-species validation of cell cycle regulators. β-scores from genome-wide CRISPR/Cas9 negative-selection screen of *PKras*^*G12D/+*^*;Snail*^*KI*^ cell lines are indicated. Genes with FDR-*q* value > 0.05 are marked with an X on the bar. **e**, **f** Kaplan-Meier analysis of PDAC patients (*n* = 111). **e** Disease-Free Survival (DFS) *p* = 0.0178 log-rank test and (**f**) Overall Survival (*p* = 0.0094 log-rank test), in samples with aberrant high SNAI1 expression (Q4) compared to the rest (Q1-3). **g** Correlation of SNAIL expression with gemcitabine treatment resistance of human PDAC patients. Density distribution of SNAI1 mRNA expression across gemcitabine resistant (*n* = 6) or sensitive (*n* = 24) samples, *p* = 0.065, two-tailed Wilcoxon rank test. Source data of Fig. 8 are provided in the Source Data file.

To assess a potential EMT-independent link of aberrant SNAIL expression with cell cycle progression in human PDAC, we analysed SNAIL and CDH1 abundance in differentiated and undifferentiated human PDAC specimens and cell lines, and analysed data of resected primary tumours^49^. We observed high levels of SNAIL expression in both differentiated and undifferentiated human PDAC specimens and cell lines in accordance with our findings in genetic mouse models corroborating EMT-independent functions of SNAIL also in human PDAC (Fig. 8a, b). In addition, we discerned undifferentiated specimens, which lack both, SNAIL and CDH1 expression (Fig. 8a). Gene expression profiling of primary resected differentiated human PDAC specimen with high CDH1 expression revealed no correlation with SNAIL abundance (*p* = 0.77; Fig. 8c). However, we observed a strong positive correlation between SNAIL and the expression of important cell cycle regulators, such as CDK4, CCNA1, CCND1, CCND2, CCNE1 and E2F3 (Fig. 8d). Strikingly, many of these genes have been identified as direct targets of SNAIL in murine PDAC (Fig. 7 and Supplementary Fig. S5) and functionally validated by genome wide CRISPR/Cas9-based negative selection screens (Fig. 8d right panel). In addition, tumours with high SNAIL expression were strongly associated with a poorer disease-free survival (DFS) and overall survival (OS) after surgical resection (Fig. 8e, f). Further, we observed a trend towards resistance against chemotherapy with gemcitabine in a small cohort with available clinical data of the resected PDAC patients (6 gemcitabine sensitive and 24 resistant PDAC cases; *p* = 0.065) (Fig. 8g). While intriguing and consistent with published experimental studies in mice, reporting that *Snail* knockout sensitizes PDAC tumours to gemcitabine treatment^13^, these human studies will require larger sample sets and prospective analyses in future.

## Discussion

Understanding the specific in vivo functions of SNAIL, which is aberrantly expressed in a wide variety of epithelial cancers and often correlated with poor patient outcome, is crucial for improving patient stratification and clinical interventions^5,14^. SNAIL has been extensively and convincingly characterized as a master regulator of the embryonic EMT program, which triggers cancer cell plasticity, migration and metastatic spread in various tumour types^5,8,11^. In contrast, little is known about EMT-independent oncogenic functions of SNAIL in cancer initiation and progression. Specifically, non-redundant functions of this EMT transcription factor (TF) in autochthonous tumours remain elusive^26^. We employed complex genetic in vivo modelling to address this important question in a comprehensive and systematic manner across different cancer types, oncogenic drivers and pathways. This enabled us to discover a complex non-redundant context-specific EMT-independent framework of SNAIL function in epithelial PDAC that bypasses oncogenic KRAS-induced senescence and drives the cell cycle by p16^INK4A^-independent inactivation of the RB-restriction checkpoint of senescence and the cell cycle. Importantly, our data demonstrate that SNAIL acts in this context as a transcriptional activator, rather than via its canonical function as a transcriptional repressor^50^; it binds directly to the canonical E-boxes of a variety of important cell cycle regulators, such as cyclins, CDKs and E2F TFs to drive the cell cycle as evidenced by ChIP experiments and reporter gene assays. This allows sustained proliferation of epithelial PDAC cells and thus tumour progression independent of overt EMT induction and contrasts with WNT- and KRAS-driven intestinal cancer subtypes, which are refractory towards aberrantly expressed SNAIL. In line with our findings, SNAIL has recently been shown to be dispensable for the EMT process in PDAC, which is controlled by the EMT transcription factor Zeb1^51^.

Collectively, our studies constitute a comprehensive analysis of SNAIL function in cancer. SNAIL has been identified and validated as an intrinsic cancer driver, and there are strong indications that both, the cell and tissue of origin as well as the genetic context dictates the function of SNAIL as a cancer driver. This improves our understanding of the diverse in vivo functions of SNAIL and will enable SNAIL downstream targets to be defined within the cell cycle machinery in epithelial PDAC. Our discovery has potentially important clinical implications, since it provides a framework for patient stratification and opens avenues for therapeutic interventions. Therapeutics targeting the cell cycle have been developed in recent years, which provide efficient opportunities to block cell cycle progression, e.g., via blockade of CDK4/6 activity^52^. Furthermore, considering the association of SNAIL with Gemcitabine resistance, it would seem worthwhile evaluating whether targeting SNAIL downstream effectors can improve the efficacy of current therapies for PDAC. Such treatment options are urgently needed. PDAC is a highly lethal and refractive disease with overall 5-year survival rates below 9%^53^.

## Methods

### Mouse strains and tumour models

*LSL-Kras*^*G12D/+*^ ^54,55^, *Pdx1-Cre*^54^, *Ptf1a*^*Cre/+*^ ^56,57^, *LSL-Trp53*^*R172H/+*^ ^33,58^, *LSL-R26*^*TvailacZ/+*^ ^59^, *Cdkn2a*^*lox/+*^ ^36^, *p16*^Ink4a*/+^ ^35^, *Villin-Cre*^60^, *LSL-Braf*^*V637E/+*^ ^21^, *Apc*^*lox/+*^ ^20^, *Cdh1*^*lox/+*^ ^61^, *Snail*^*lox/+*^ ^62^ mice have been previously reported. All strains were on a mixed C57Bl/6 J;129S6/SvEv genetic background and interbred to obtain compound mutant mice of both sexes that develop autochthonous tumours in the pancreas and intestine. The sex, substrain, age and number of all animals analysed in this study in every experiment is provided in the “Source Data” Excel file. All animal studies were conducted in compliance with European guidelines for the care and use of laboratory animals and were approved by the Institutional Animal Care and Use Committees (IACUC) of the local authorities of Technische Universität München and the Regierung von Oberbayern (animal protocol number: ROB-55.2-2532.Vet_02-17-79 and 55.2-1-54-2532-31-11). The maximal tumour size/burden permitted by the IACUC and the local authorities (Regierung von Oberbayern) is 1.5 cm in diameter, which was not exceeded in our study. Euthanasia was performed by cervical dislocation. Animals were housed under specific pathogen- free conditions (SPF) in a dedicated facility, with a light-dark cycle of 12:12 hours, a relative air humidity between 45 and 65% and a temperature between 20 and 24 °C.

### Construction of the targeting vector and generation of the *LSL-Rosa26*^*Snail*^ mouse line

*Rosa26* targeting by a knock-in strategy was performed based on the *pROSA26–1* plasmid^59^. Murine Snail cDNA (Snai1 cDNA; Library: IRAV MGC Mouse verified full length amplified cDNA; Clone: IRAVp968A0443D6, German Science Centre for Genome Research) was cloned into the targeting vector 3´ of a loxP-flanked transcriptional and translational stop element (loxP-stop-loxP, LSL) with a neomycin resistance cassette (Supplementary Fig. S1a). The targeting vector was linearized, electroporated into W4/129S6 embryonic stem cells, selection with 250 µg/ml geneticin imposed, and correctly targeted cell clones identified by PCR^59^. Gene targeting was verified by Southern blot with an external ^32^P-labeled 5´ probe and EcoRV digested genomic DNA (Supplementary Fig. S1b). The Southern blot images were processed with an Amersham automatic Hyperprocessor (Amersham Biosciences). Two verified cell clones were injected into C57BL/6 J blastocysts (Polygene). Germ-line transmission was achieved in 2/2 clones harbouring the targeted allele. The mice were genotyped using a 3-primer PCR strategy (ref. ^59^, Table 1 and Supplementary Fig. S1c).

**Table 1:** Recombination PCR primers for *LSL-Rosa26*^*Snail*^ allele

Name of PCR	Name of primer	Sequence (5’ – 3’)
LSL-Rosa26^Snail^ recombination	R26-GT forward	AAAGTCGCTCTGAGTTGTTAT
	R26-Stop cassette reverse	TGAATAGTTAATTGGAGCGGCCGCAATA
	Snail-cds reverse	GCGCTCCTTCCTGGT

### Transduction of tumour cells using the RCAS-TVA system

To overexpress SNAIL in cell lines via the RCAS-TVA system^59,63^, the murine *Snail* cDNA was amplified and cloned into the pCR-Blunt II-TOPO vector (Invitrogen). After AatII/NdeI digestion, *Snail* cDNA was ligated to a modified pENTR/D-TOPO (Invitrogen) vector carrying dsRed under the control of the *EF1α* promoter 3’ to the *Snail* insertion side. Further cloning into RCASBP(A)-Att-CCDB-Att (modified from RCASBP(A), kindly provided by Stephen H. Hughes) was performed using the GatewayR LR Clonase (Invitrogen) mix to generate the final retroviral construct.

To generate RCAS vectors, the chicken fibroblast cell line DF-1 (American Type Culture Collection # CRL-12203 (RRID:CVCL_0570) was transfected using Superfect (Qiagen) with 2.5 µg purified RCAS plasmid. Fresh virus-containing supernatant was filtered through 0.45 μm pores and added to the medium of murine tumour cells carrying the TVA receptor^59^. Transduction with fresh supernatant was repeated daily until 80% cells showed expression of the dsRed reporter gene (Supplementary Fig. S3f).

### Histology and immunohistochemistry

Murine tissue specimens were fixed overnight in 4% buffered formalin, dehydrated, embedded in paraffin and sectioned (2.5 µm thick). ADM, PanIN lesions and intestinal adenomas and carcinomas were quantified using haematoxylin and eosin (H&E)-stained sections^64^. Quantification was carried out blinded to the genotype.

TUNEL staining was conducted using the In Situ Cell Death Detection Kit, POD (Roche). Alcian blue staining and immunohistochemistry were performed using standard procedures^64^. If not stated otherwise, antigen retrieval was performed in citrate buffer, pH 6.0 in a microwave oven. The following primary antibodies were used: Muc5a (antigen retrieval Tris/EDTA pH 9.0, 1:200, 45M1 #MS-145-P1, Neomarkers), Cytokeratin 19 (1:300, TROMA 3 Developmental Studies Hybridoma Bank), E-cadherin (1:100, #610181, BD Biosciences), Rabbit anti-Ki67 (1:50, #MA5-14520, SP6, ThermoFischer), p-γ-H2AX (1:500, #05-636, Millipore), Cleaved Caspase 3 (1:250, #9664, Cell Signaling Technology), BrdU (1:500, #MCA2060, AbD Serotec), pRB (1:100, #8516, Cell Signaling Technology), p16^INK4A^ (1:50, #sc-1661, Santa Cruz Biotechnology), TRP53 (1:400, #NCL-p53-CM5p, Novocastra/Leica Microsystems), p21^CIP1^ (1:50, #sc-397, Santa Cruz Biotechnology), SNAIL (1:50, #3879, Cell Signaling Technology).

For BrdU assay, 5 mg/kg 5-bromo-2’-deoxyuridine (BrdU), dissolved in sterile PBS, was injected intraperitoneally into animals 2 h before sacrifice.

Images were acquired with AxioVision Rel 4.8 and Aperio ImageScope v12.3.3. For counting of BrdU-, pRB-, and p-γ-H2AX-positive cells in ADMs and PanINs, one- to three-months old *PKras*^*G12D/+*^*;Snail*^*KI/+*^ mice and one-month to two-year old *PKras*^*G12D/+*^ animals were used. Quantification was carried out blinded to the genotype.

### Metastasis quantification

At sacrifice, abdominal organs and lungs were investigated macroscopically for metastases^17,65^. Macroscopic pictures were taken using a Stemi SV 11 stereomicroscope (Zeiss) and processed with AxioVision Rel 4.8 software. For microscopic quantification, at least ten series of sections (100 µm between each series) of paraffin-embedded lungs and livers were prepared, H&E stained and investigated for the presence of metastases. Quantification was carried out blinded to the genotype.

### Senescence-associated β-galactosidase (SA-β-gal) analysis

To obtain cryosections, tissue was fixed in 4% buffered formalin for 2 h, dehydrated in a sucrose series (15% sucrose for 4 h and 30% sucrose overnight), embedded in Tissue-Tek (Sakura Finetek), snap-frozen and sectioned (6 µm). Sections were dried overnight, and staining performed using the Senescence β-Galactosidase Staining Kit (Cell Signaling Technology)^66^. ADMs and PanINs from three different slides per pancreas were assessed for SA-β-gal quantification. The number of cells displaying positive SA-β-gal staining was counted and divided by the total number of cells per PanIN lesion, and expressed as % positive cells per lesion in the respective graphs. Quantification was done blinded to the genotype. SA-β-gal staining of cells in culture was performed as recommended by the manufacturer of the the Senescence β-Galactosidase Staining Kit and quantified blinded to the genotype as % positive cells.

### Immunocytochemistry

Cells were washed 3 times in cold PBS and fixed 10 min in cold methanol. Washing was repeated following permeabilisation in 0.3% Triton X-100 in PBS for 10 min. Blocking was done for 30 min at 37 °C with 5% donkey serum before incubation with the primary E-cadherin antibody (1:80, #AF748, R&D Systems) for 2 h at 37 °C. After washing 3 times with PBS, cells were incubated with secondary antibody (Alexa Fluor® 488 donkey anti-goat, 1:100, # A-11055, Invitrogen) for 30 min at 37 °C. Washing was repeated and cells were covered with a cover glass using Vectashield mounting medium with DAPI. Images of the slides were acquired with AxioVision Rel 4.8 and Aperio ImageScope v12.3.3.

### Cell lines and cell culture

Primary PDAC cell cultures were isolated from autochthonous mouse PDAC tumours and cultured in DMEM medium with 10% Fetal Bovine Serum^17,65^. The following human PDAC cell lines from from the American Type Culture Collection (ATCC), German Collection of Microorganisms and Cell Cultures (DSMZ) or Cell bank were used: AsPC-1 (CVCL_0152) ATCC# CRL-1682; Capan-2 (CVCL_0026) ATCC# HTB-80; CFPAC-1 (CVCL_1119) ATCC# CRL-1918; DAN-G (CVCL_0243) DSMZ# ACC 249; HPAC (CVCL_3517) ATCC# CRL-2119; HPAF-II (CVCL_0313) ATCC# CRL-1997; Hs 766 T (CVCL_0334) ATCC# HTB-134; HuP-T4 (CVCL_1300) DSMZ# ACC 223; IMIM-PC1 (CVCL_4061) https://www.cellosaurus.org/CVCL_4061; KP-4 (CVCL_1338) Cell bank# RCB1005; MIA PaCa-2 (CVCL_0428) ATCC# CRL-1420; Panc 02.03 (CVCL_1633) ATCC# CRL-2553; Panc 03.27 (CVCL_1635) ATCC# CRL-2549; Panc 04.03 (CVCL_1636) ATCC# CRL-2555; Panc 05.04 (CVCL_1637) ATCC# CRL-2557; Panc 08.13 (CVCL_1638) ATCC# CRL-2551; PANC-1 (CVCL_0480) ATCC# CRL-1469; Panc 10.05 (CVCL_1639) ATCC# CRL-2547; PaTu 8902 (CVCL_1845) DSMZ# ACC 179; PaTu 8988 s (CVCL_1846) DSMZ# ACC 204; PL45 (CVCL_3567) ATCC# CRL-2558; PSN1 (CVCL_1644) ATCC# CRL-3211; SU.86.86 (CVCL_3881) ATCC# CRL-1837; SW1990 (CVCL_1723) ATCC# CRL-2172; YAPC (CVCL_1794) DSMZ# ACC 382^67^. The Human Pancreatic Duct Epithelial (HPDE) cells (H6c7; RRID: CVCL_0P38) were obtained from Kerafast (#ECA001-FP), and the avian TVA receptor positive chicken embryonic fibroblast cell line DF-1 (RRID:CVCL_0570) from ATCC# CRL-12203.

All human cell lines were authenticated through STR or SNP profiling (last correct authentication in 2022). All murine cell lines were re-genotyped and tested for correct recombination of the respective alleles (last re-genotyping in 2022). The chicken fibroblast cell line DF-1 was authenticated by genotyping PCR for presence of the avian TVA receptor. All cells used were cultivated for less than 30 passages and tested negative for mycoplasma contamination by PCR. The electrophoresis DNA gel pictures were acquired with the Gel Doc™ XR + system (Biorad).

### PDAC cell doubling time calculation

For PDAC cell doubling time calculation, 1000–2000 cells per well were seeded out in triplicates in 96 Well plates. Cell viability was determined on the following day (Day 0) and again 72 hours after the initial measurement (Day 3) by CellTiter-Glo assay (Promega). Doubling times were calculated blinded to the genotype by the formula given in equation number 1:

Equation Number 1:

$${DoublingTime}=72\,{hours} * \frac{{{\log }}\left(2\right)}{{{\log }}\left(\frac{{mean\, CellTiter\, Glo\, value\, on\, Day\, 3}}{{mean\, CellTiterGlo \, value \, on \, Day \,0}}\right)}$$

### Stimulation of PDAC cells with TGFβ

Cells at 50% confluence were cultured for 24 h in FCS-free DMEM before treatment with 10 ng/ml TGFβ or vehicle (10 nM citric acid, 2 mg/ml BSA). Cell morphology was documented after 72 h.

### Acinar explants and acinar-ductal metaplasia (ADM) assay

Directly after sacrifice, pancreata of one-month old mice were injected with 2 ml Collagenase P solution (1.33 mg/ml Collagenase P (Roche) in HBSS (Gibco)), cut out, minced with a scalpel and gently shaken for 30 min at 37 °C in 5 ml Collagenase P solution. All subsequent steps were performed at 4 °C in a laminar flow cabinet and all centrifugation steps were carried out for 3 min at 180 x g. Cells were resuspended in 10 ml 5% FBS in HBSS and incubated 10 min for sedimentation of the cellular fraction. Supernatant was aspirated carefully, and the pellet was washed 3 times with 5% FBS in HBSS. Cells in 10 ml 5% FBS in HBSS were transferred into a new tube through a 100 µm cell strainer, slowly laid over 20 ml 30% FBS in HBSS and centrifuged. Cells were resuspended in 2 ml recovery medium (acinar cell medium, see below, with 30% FCS), incubated at 37 °C for 1 h, centrifuged and resuspended in a 1:1 mixture of acinar cell medium (containing 0.1% bovine serum albumin, 0.2 mg/ml soybean trypsin inhibitor (Sigma), 1% ITS premix (Corning), 50 µg/ml bovine pituitary extract (ThermoFisher), 0.1% FBS, 0.5% penicillin/streptomycin, 0.25 µg/ml Fungizone antimycotic (ThermoFisher) in Waymouth’s medium (Gibco) and rat tail collagen type I (Corning). Per pancreas, cells were seeded into 16 wells of a 48-well plate on a previously prepared collagen layer (final collagen concentration 2.5 mg/ml) and covered with another collagen layer before adding acinar cell medium. Medium was changed every 24 h.

Five days after seeding, images were acquired with AxioVision Rel 4.8 software and the percentage of ductal structures of the total amount of acinar explants was determined by counting 5 microscopic fields of view at 100x magnification for each pancreas. Quantification was blinded to the genotype.

### Whole cell lysates and western blot

Whole cell lysates and proteins from tissue were harvested and subjected to western blotting using the following primary antibodies: SNAIL (1:500, #3895, Cell Signaling Technology), E-cadherin (1:2000, #610181, BD Biosciences), HSP90 (1:250, #sc-13119, Santa Cruz Biotechnology), TRP53 (1:1000, #NCL-p53-CM5p, Novocastra/Leica Microsystems), p21^CIP1^ (1:200, #sc-397, Santa Cruz Biotechnology), β-Actin (1:4000, #A5316, Sigma-Aldrich) and α-Tubulin (1:5000, #T9026, Sigma-Aldrich). The western blot images were collected using the Odyssey infrared imaging system with the Odyssey Software V1.2 (Li-Cor Biosciences).

### Quantitative real-time PCR (qPCR)

Total RNA was isolated from tissues and cell lines with the RNeasy Kit (Qiagen) following reverse transcription (Applied Biosciences). 1 µg RNA was used for generation of 50 µl cDNA. qPCR was performed with the StepOnePlus real time PCR system (Applied Biosystems) by using the StepOne Software v2.3. Power SYBR Green PCR Master Mix was used in a 25 µl mixture containing 100 nM of each primer. Only primers with an amplification efficiency between 1.8 and 2.2 were applied. qPCR primers are given in Table 2. mRNA expression was analysed on 5 µl of 1:5 diluted cDNA in either duplicate or triplicate. All expression values were normalized to the housekeeping gene Cyclophilin A (CypA) or GAPDH. A melt curve was performed after each run to check for unwanted primer dimerization. Data analysis was carried out using Excel version 16.65 (Microsoft Corporation) according to 2^-ΔΔCt^ method.

**Table 2:** qPCR primers for testing mRNA expression level

Gene	Name of primer	Sequence (5’ – 3’)
*CDH1 human*	hCDH1-forward hCDH1-reverse	CCGAGAGCTACACGTTC TCTTCAAAATTCACTCTGCC
*Cdh1 mouse*	mCdh1-forward mCdh1-reverse	GAGCGTGCCCCAGTATCG CGTAATCGAACACCAACAGAGAGT
*p16*^*Ink4a*^	mp16-forward mp16-reverse	CCCAACGCCCCGAACT GTGAACGTTGCCCATCATCA
*p19*^*Arf*^	mp19-forward mp19-reverse	TCGCAGGTTCTTGGTCACTGT GAACTTCACCAAGAAAACCCTCTCT
*Ccna1*	mCcnA1-forward mCcnA1-reverse	GCTGTCTCTTTACCCGGAGCA ACGTTCACTGGCTTGTCTTCTA
*Ccna2*	mCcnA2-forward mCcnA2-reverse	CACTGACACCTCTTGACTATCC CGTTCACTGGCTTGTCTTCT
*Ccnb1*	mCcnB1-forward mCcnB1-reverse	TTGTGTGCCCAAGAAGATGCT GTACATCTCCTCATATTTGCTTGCA
*Ccnb2*	mCcnB2-forward mCcnB2-reverse	TGAAGTCCTGGAAGTCATGC GAGGCCAGGTCTTTGATGAT
*SNAIL human*	hSNAIL-forward hSNAIL-reverse	CTCTAATCCAGAGTTTACCTTC GACAGAGTCCCAGATGAG
*SNAIL mouse*	mSNAIL-forward mSNAIL-reverse	GCCGGAAGCCCAACTATAGC GGTCGTAGGGCTGCTGGAA
*KRAS human*	hKRAS-forward hKRAS-reverse	GACTGAATATAAACTTGTGGTAGTTGGA CATATTCGTCCACAAAATGATTCTG
*Cyclophilin A (CypA)*	CypA-forward CypA-reverse	ATGGTCAACCCCACCGTGT TTCTTGCTGTCTTTGGAACTTTGTC
*GAPDH human*	hGAPDH-forward hGAPDH-reverse	AATCCCATCACCATCTTCCA TGGACTCCACGACGTACTCA

### Analysis of p16^Ink4a^ genomic sequence integrity

Genomic DNA was isolated from PDAC cell lines using the DNeasy Blood & Tissue Kit (Qiagen). The integrity of the p16^Ink4a^ locus was tested by PDAC amplification and gel electrophoresis using 10 ng DNA and the primers given in Table 3. GABRA was used as positive control.

**Table 3:** Primers for testing p16^Ink4a^ genomic sequence integrity

Name of PCR	Name of primer	Sequence (5’ – 3’)
*p16*^*Ink4a*^ integrity	p16^Ink4a^-forward p16^Ink4a^-reverse	AGTTCGGGGCGTTGGG GCACAGGCTCTGGAATGCA
*Gabra*	Gabra-forward Gabra reverse	AACACACACTGGAGGACTGGCTAGG CAATGGTAGGCTCACTCTGGGAGATGATA

### Quantitative chromatin immunoprecipitation (ChIP)

ChIP was performed using SimpleChIP Enzymatic Chromatin IP Kit (#9003, Cell Signaling Technology) according to the manufacturer’s protocol using SNAIL antibody (1:50, #3879, Cell Signaling Technology) and rabbit IgG (1:50, #2729, C15D3, Cell Signaling Technology) as negative control and H3 (1:50, #2650, Cell Signaling Technology) as positive control. Binding of SNAIL to the DNA regions of interest was determined by qPCR using the primers listed in Table 4 and analysed by the percent input method^68^.

**Table 4:** qPCR primers for testing E-box binding by ChIP

Gene	Name of primer	Sequence (5’ – 3’)
*Ccna1*	CcnA1-Ebox-forward CcnA1-Ebox forward	TTAAAGCCCATTCAGCCATTGTT TGTCCCAACTTCCCGACAAAC
*Ccnb1*	CcnB1-Ebox-forward CcnB1-Ebox-reverse	CATTGCTGCCACCTGCCTTA ATGCGTACTCCCCACAGTCA
*Ccnb2*	CcnB2-Ebox-forward CcnB2-Ebox-reverse	CATCGTCTCCAGGTCGTTCA ATGACTCTGCTGGGGATCTGT
*Ccnd1*	CcnD1-Ebox-forward CcnD1-Ebox-reverse	AGCGTCCCTGTCTTCTTTCAA GTCTGGCATCTTCGGGTGTT
*E2f2*	E2F2-Ebox-forward E2F2-Ebox-reverse	TGCCTCAGTTTCGCCTACTG ACAGCGATTACGACAGGAGC
*E2f3*	E2F3-Ebox-forward E2F3-Ebox-reverse	GCGCAAGTTTCGGTTTTGG CTACACTGCTTGGTTACAGGA

### Chromatin immunoprecipitation coupled to mass spectrometry (ChIP-MS)

ChIP was performed using freshly prepared cell lysates of murine primary PDAC cells (P144) isolated from the *PKras*^*G12D/+*^*;Snail*^*KI/+*^ model. For each condition, three biological replicates were used. Briefly, 10^7^ cells were fixed in 1% v/v formaldehyde (FA) in Phosphate buffered saline (PBS) at room temperature (RT) for 10 min. After incubating with 1.25 M glycine and washing twice with PBS, the samples were resuspended in IP buffer (50 mM Tris-HCl pH 8.0, 100 mM NaCl, 5 mM EDTA pH 8.0, 1.7% v/v Triton X-100, 0.3% v/v SDS, protease and phosphatase inhibitors). Subsequently, chromatin was sonicated (4 × 10 cycles at 4 °C; 30 s ON, 30 seconds OFF each cycle) using a Bioruptor Plus (Diagenode, Denville, NJ, United States) to an average size of 500 bp. The samples were then centrifuged at 3500 x *g* for 20 min at 4 °C, and the supernatant was used for estimation of protein concentration with the bicinchoninic acid (BCA) assay based on the manufacturer’s instructions (Pierce™ BCA Protein Assay Kit, Thermo Fisher Scientific). 1 mg of extract from each sample was used for immunoprecipitation with anti-Snail Rabbit mAb (1:50, #3879, C15D3, Cell Signaling) or anti-IgG Rabbit Ab (#2729, Cell Signaling, 5 µg) by incubating overnight at 4 °C on a rotating wheel. The next day, the antibody-bound complexes were precipitated with protein A + G-coupled magnetic beads (Millipore, Sigma) washed three times with low salt buffer (50 mM HEPES pH 7.5, 140 mM NaCl, 1% v/v Triton X-100), once with high salt buffer (50 mM HEPES pH 7.5, 500 mM NaCl, 1% v/v Triton X-100) and once with TBS. Immunoprecipitates were eluted from the beads and digested after incubating with the freshly prepared elution buffers I (2 M Urea, 50 mM Tris-HCl pH 7.5, 2 mM Dithiothreitol, 20 µg/mL Trypsin) and II (2 M Urea, 50 mM Tris-HCl pH 7.5, 10 mM Chloroacetamide) for 30 min and 5 min at 37 °C, respectively. Both eluates were combined and further incubated overnight at 25 °C. Subsequently, the tryptic peptides were acidified with 1% v/v Trifluoroacetic acid (TFA) solution, and transferred on top of styrene-divinylbenzene reverse-phase sulfonate (SDB-RPS; three layers) in-house made StageTips for desalting. Finally, they were concentrated (45 °C, 20 min) using a centrifugal evaporator (Eppendorf) until dryness, and analszed by liquid chromatography-coupled to mass spectrometry (LC-MS/MS) after reconstitution in MS compatible buffer [2% acetonitrile (ACN) v/v, 0.1 % v/v TFA]^69^.

### LC-MS/MS analysis and data processing

All peptide samples were measured in a single-shot manner in a Q-Exactive HF-X hybrid quadrupole-orbitrap mass spectrometer (Thermo Fisher Scientific) after peptide separation by high-performance liquid chromatography (nanoLC 1200, Thermo Fisher Scientific) using a 50 cm reversed-phase column (made in house, packed with 1.9 µm C18 ReproSil particles). Peptides were eluted over a 90-minute-gradient from 0% to 95% buffer B (0.1% formic acid and 80% ACN) with a flow rate of 300 nL/minute.

Full scans were obtained from 300 to 1650 m/z with a target value of 3 × 10^6^ ions at a resolution of 60,000 at 200 m/z. The fifteen most intense ions (Top15) of each full scan were fragmented with higher-energy collisional dissociation (HCD) (target value 1 × 10^5^ ions, maximum injection time 120 ms, isolation window 1.4 m/z, underfill ratio 1%), and fragments were detected in the Orbitrap mass analyzer at a resolution of 15,000 at 200 m/z.

### Mass spectrometry data analysis

Raw MS data files were processed using MaxQuant (version 1.6.1.0) to calculate peptide intensities with the integrated Andromeda search engine with FDR < 0.01 both at the protein and peptide levels. Oxidized methionine (M) and acetylation (protein N-terminus) were set as variable modifications, and carbamidomethyl (C) as fixed modification. Only peptides with a minimal length of seven amino acids were considered and the “match between runs” option was enabled for the biological replicates within each condition with a matching time window of 0.7 min. For protein and peptide identification, the UniProt database from mouse (September 2014) including 51,210 entries were used. Each raw file and biological replicate was treated as one independent experiment.

For bioinformatics analyses, the Perseus platform^70^ (version 1.6.7.0) was used. The R environment (version 3.6.2) was used for data visualization. Pre-processing of the label-free proteomics data included: (a) exclusion of reverse, potential contaminants and peptide identified only by site, (b) log2 transformation of peptide intensities, and (c) peptides without intensity values in less than 67% of the values in at least one bait group were filtered out. Missing values were replaced from a normal distribution window (width 0.3, downshift 1.8 standard deviations).

For statistical analysis, the two-sample *t*-test was implemented (FDR < 0.05, s0 = 1) and identified 141 proteins as significant (out of 1039 quantified proteins) between the two conditions (bait vs. negative control).

### Lentivirus production and transduction

For lentivirus production, HEK293FT cells were transfected using TransIT-LT1 (Mirus Bio LLC) transfection reagent according to the manufacturer’s instructions with lentiviral packaging plasmids psPAX2 and pMD2.G and the respective lentiviral transfer plasmids. Virus-containing supernatant was collected 48 and 72 h after transfection, filtered through a 0.45 µm filter and stored at −80 °C. Cell lines were transduced in the presence of 8 μg ml^−1^ polybrene and selected with the respective selection antibiotic (Puromycin or Blasticidin).

### Inducible activation of KRAS^G12D^ and Snail in HPDE Cells

Inducible expression vectors for GFP and mutant Kras^G12D^ based on the pInducer20 vector system have been used in HPDE cells^32^. To generate an inducible expression system for Snail, cDNA of human SNAIL was cloned into the pInducer20-Blast (RRID:Addgene 109334)^71^ and verified by sequencing.

HPDE cells were cultivated in Keratinocyte-SFM medium (ThermoFisher). To induce expression of the respective target genes, cells were treated for the indicated time points with 100 ng ml^−1^ doxycycline.

### Promoter reporter assays

The promoter reporter constructs for E2F2 (#MPRM38445-LvPG04-GC), E2F3 (MPRM40957-LvPG04-GC), CCNB1 (MPRM49947-LvPG04-GC) and CCNB2 (MPRM39222-LvPG04-GC) were purchased from BioCat GmbH (Heidelberg, Germany) and transduced into *PKras*^*G12D/+*^ *and PKras*^*G12D/+*^*;Snail*^*KI/KI*^ PDAC cell lines.

The Secrete-Pair Dual Luminescence Assay Kit (#LF032-GC, BioCat GmbH, Heidelberg, Germany) was used according to the manufacturer’s instructions to analyse reporter activity blinded to the genotype. In brief, cell lines transduced with the reporter constructs were seeded in 6-well plates and medium was collected after 24 h. For measurement of Gaussia Luciferase (GLuc), 10 µL of the collected culture medium were pipetted in duplicates into a white opaque 96-well plate. GLuc Assay Working Solution was prepared with Buffer GL-S according to the manufacturer’s instructions and 100 µL was added per well. After incubation for 1 min at room temperature, luminescence was measured using a CLARIOstar microplate reader (BMG Labtech GmbH). For transduction normalization, Secreted Alkaline Phosphatase (SEAP) was measured. Therefore, medium was heated at 65 °C for 15 min and SEAP Assay Working Solution prepared according to the manufacturer’s protocol. 100 µL SEAP Assay Working Solution were added to 10 µL medium per sample in duplicates in a white opaque 96-well plate. Luminescence was measured after 5 min incubation at room temperature in a CLARIOstar microplate reader (BMG Labtech GmbH). Ratios of the mean Gaussia Luciferase (GLuc) to the mean Secreted Alkaline Phosphatase (SEAP) were calculated to determine reporter activity.

pGL3 Basic luciferase reporter plasmids containing *Cyclin D1* (*Ccnd1*) promoter fragments (RRID:Addgene_32727 and RRID:Addgene_32726)^72^ were used to determine Cyclin D1 promoter activity blinded to the genotype. *PKras*^*G12D/+*^ *and PKras*^*G12D/+*^*;Snail*^*KI/KI*^ PDAC cell cultures were transfected with the reporter constructs using Effectene Transfection Reagent (Qiagen, Hilden, Germany) according to the manufacturer’s recommendations. In each sample, 40 ng phRL-TK (Promega) Renilla Luciferase control reporter vector was co-transfected as an internal control for transfection efficiency. The medium was changed on the next day and the Dual-luciferase reporter assay system (Promega) was used according to the manufacturer’s instructions to determine luciferase activity 48 h post transfection.

### Genome-wide CRISPR/Cas9 negative selection screens

*PKras*^*G12D/+*^*;Snail*^*KI*^ and *PKras*^*G12D/+*^*;Snail*^*KO/KO*^ Cas9-expressing cell lines were used to perform the genome-wide CRISPR/Cas9 loss-of-function screens at 500x coverage, as in^48^. Briefly, cells were transduced with the Brie library (Addgene #73633) and the screens were performed in side-by-side duplicates. At the end of the experiment, cells were harvested, genomic DNA was isolated using the Blood & Cell Culture DNA Maxi Kit (Qiagen), and sgRNA libraries were generated. The pooled sgRNA libraries were sequenced using an Illumina NextSeq 500 (custom read and indexing primers spiked in) with a depth of 35 Mio reads^48^. MAGeCK v0.5.9.4^73^ was used for downstream analysis and β-scores calculated with the maximum likelihood estimation (mle) method by employing data of non-targeting control sgRNAs. The obtained β-score depicts enrichment (positive score) or depletion (negative score) of the sgRNAs compared to their initial abundance. To calculate selectively depleted genes in the *PKras*^*G12D/+*^*;Snail*^*KI*^ model, the difference in the β-score between *PKras*^*G12D/+*^*;Snail*^*KI*^ and *PKras*^*G12D/+*^*;Snail*^*KO/KO*^ PDAC cells was determined. Enrichments were performed on the genes annotated as non-essential, presenting an FDR ≤ 0.05 in both *PKras*^*G12D/+*^*;Snail*^*KI*^ and *PKras*^*G12D/+*^*;Snail*^*KO/KO*^ cells, and showing a difference in beta score ≤ −1 by using the MSigDB v7.1 gene sets provided by Broad Institute, Massachusetts Institute of Technology and Harvard University as in^48^.

### Gene expression profiling and gene set enrichment analysis (GSEA)

mRNA was extracted using the RNeasy Kit (Qiagen). Quality was checked using the Experion RNA StdSens analysis Kit (Bio-Rad). For mRNA analysis of PDAC cell lines, 250 ng of each sample were processed with the GeneChip 3’ IVT express Kit (Affymetrix). Fragmentation and hybridization to GeneChip mouse genome 430 2.0 array chips (Affymetrix) was performed by the Institute for Medical Microbiology, Immunology and Hygiene, Technische Universität München. For mRNA analysis of pancreatic and PDAC tissues, 500 ng isolated mRNA was processed with the Ambion WT expression Kit (Applied Biosystems). Fragmentation and labelling were performed using the GeneChip WT terminal labelling Kit (Affymetrix). Hybridization to GeneChip mouse gene 1.0 ST array chips (Affymetrix) was carried out by the Institute for Medical Microbiology, Immunology and Hygiene, Technische Universität München. Data were collected with an Affymetrix Scanner 3000 7 G and the Affymetrix GeneChip Command Console Software (AGCC). The expression intensity of each gene was determined by using the Affymetrix Microarray Analysis Suite (MAS) 5.0 software.

All analyses were carried out using R version 3.1.2^74^ and Bioconductor version 3.0^75^. Microarray data were processed with the RMA method^76^, following quantile normalization^77^. For initial correlation analysis, pairwise Pearson correlation was computed on the normalized intensity values. Differential gene expression between mesenchymal and epithelial cell lines was analysed with Limma version 3.22.0^78^. A probe set was considered to be differentially expressed with a Benjamini-Hochberg adjusted^79^ *p*-value of 0.05 and an absolute fold change >2. Annotations were downloaded from ENSEMBL (GRCm38.p3)^80^. The top 50 upregulated or downregulated genes were hierarchically clustered with Ward’s minimum variance method^81^. The dissimilarities between samples were squared before cluster updating as implemented in R.

We performed gene set enrichment analysis (GSEA)^82^ using GSEA v3.0 jar package and MSigDB v6.2 gene sets provided by Broad Institute of Massachusetts Institute of Technology and Harvard University. GSEA was conducted with RMA normalized microarray data. Parameters were set as follows: phenotype was defined as “*PKras*^*G12D/+*^*;Snail*^*KI/+*^“ versus “*PKras*^*G12D/+*^“; gene sets were permuted for 1000 times; enrichment statistic for scoring was set as “weighted” and genes were ranked based on “*t*-Test” metric; other parameters were set as default. The cut-off for a significant FDR *q*-value as well as NOM *p*-value was set at 0.05.

### Human primary PDAC cohort

RNAseq data from resected primary PDAC tumours are accessible via International Cancer Genome Consortium (ICGC), as reported in Connor et al.^49^ were analysed using R version 4.2.12. Adapters and bad quality reads were trimmed with Trimmomatic version 2.38^83^. Filtered reads were aligned to human genome (hg19) and quantified using STAR version 2.6.0^84^. Raw read count per gene was normalized to the library size using Counts Per Million (CPM). The resected cohort (*n* = 177) was divided into epithelial-like (*n* = 88) and mesenchymal-like samples (*n* = 89) based on CDH1 expression. Samples with high CDH1 expression, i.e., above CDH1 median expression, were classified as epithelial subtype, whereas low CDH1 expression samples were considered as mesenchymal subtype. Epithelial samples were further divided according to the expression of SNAI1 mRNA using quartile distribution were Q1 and Q4 contain the samples with the lowest and highest SNAI1 expression respectively. Differential expression of cell cycle-related genes across Q1 to Q4 subgroups was determined using an ANOVA test. Top 20 significantly altered cell-cycle genes across SNAI1 quartiles are depicted on the heatmap (see Fig. 8d).

Among the epithelial-like PDAC cohort, 32 patients received adjuvant chemotherapy with Gemcitabine. 24 were sensitive and 6 resistant. The response of 2 patients is unknown. The differential expression of SNAI1 mRNA between Gemcitabine resistant (*n* = 6) and sensitive (*n* = 24) samples was assessed using Wilcoxon rank test.

Survival analysis was performed on the complete resected dataset (111/177 samples with follow-up annotation). Samples with aberrant SNAI1 expression (Quantile 4, Q4) were compared to the other samples (Q1 to Q3). Difference of survival was determined with a Cox proportional hazards regression model. *P*-value below 0.05 was considered as significant.

To assess the expression of SNAIL in human PDAC sections of differentiated (G1/2) and undifferentiated tumours (G3/4), we used publicly available immunohistochemical stainings of the Human Protein Atlas version 20.1^85^, which are available from https://www.proteinatlas.org/ENSG00000124216-SNAI1/pathology/pancreatic+cancer#ihc, as well as a cohort of PDAC tissue samples purchased from Biomax.us (PA961a Pancreatic cancer tissue array with normal pancreatic tissue, https://www.biomax.us/PA961a) and stained for SNAIL and CDH1.

### Additional statistical methods and data analysis

No statistical method was used to determine sample size a priory. In Supplementary Fig. 1e, one outlier in the *PKras*^*G12D/+*^*;Snail*^*KI/+*^ cohort that differed significantly from the other observations, has been removed from the analysis (please see Source Data file, Supplementary Fig. 1e, for detailed information on outlier definition). No other data were excluded from other datasets. Randomization was not appropriate for experiments described in this study. The investigators were blinded to allocation during experiments and outcome assessment. Graphical depiction and statistical analysis were performed with GraphPad Prism v5 and v8. Unless otherwise indicated, all data were determined from at least 3 independent experiments and expressed as mean values ± SEM. For comparisons between data sets, log-rank test, Fisher’s exact test, one- or two-tailed *t*-test with or without Welch’s correction or Mann-Whitney test were employed and resulting p-values are indicated in the respective figures. The significance level was set to 0.05. If more than one statistical test was performed simultaneously on a single data set, a Bonferroni-adjusted significance level was calculated to account for the increased possibility of false-positive results. Percentage of mice with intestinal carcinoma, cell morphology and *p16*^*Ink4a*^ genomic sequence integrity were compared by Fisher’s exact test. Metastasis rates were compared by Fisher’s exact test followed by multiple testing correction with Benjamini Hochberg procedure. Survival analysis of the mouse models was carried out by the log-rank test.

### Reporting summary

Further information on research design is available in the Nature Portfolio Reporting Summary linked to this article.

## Supplementary information

Supplementary Information

Description of Additional Supplementary Files

Supplementary Data 1

Supplementary Data 2

Reporting Summary

## Data Availability

All microarray data generated in this study have been deposited in the ArrayExpress database (https://www.ebi.ac.uk/arrayexpress/) with accession # E-MTAB-8173 and # E-MTAB-8174. Protein data generated by mass spectrometry have been deposited in the PRIDE database under accession code # PXD038726 The ChIP-MS and genome wide CRISPR/Cas9 negative selection screen data generated in this study are provided in the Supplementary Data file 1 and 2, respectively. In addition, source data (raw and processed data) for all data presented in graphs are provided as source data with this paper. Mice and cell lines are available from the corresponding author on request. Source data are provided with this paper.

## Source data

Source Data
