# Supplementary material for: Non-canonical functions of SNAIL drive context-specific cancer progression
Source: Nat Commun. 2023 Mar 7;14:1201. doi: 10.1038/s41467-023-36505-0 (PMC9992512; doi:10.1038/s41467-023-36505-0)
Supplement: Supplementary file 1 — Supplementary Information [file 41467_2023_36505_MOESM1_ESM.pdf]

Non-canonical functions of SNAIL drive context-specific cancer progression

Mariel C. Paul[1,2#], Christian Schneeweis[1-4#], Chiara Falcomatà[1-3#], Chuan Shan[1,2#], Daniel Rossmeisl[1-3], Stella Koutsouli[5], Christine Klement[3,6,7], Magdalena Zukowska[1-3], Sebastian A. Widholz[3,6,7], Moritz Jesinghaus[1-3,8,9], Konstanze K. Heuermann[1-3], Thomas Engleitner[3,6,7], Barbara Seidler[1-3], Katia Sleiman[1-3], Katja Steiger[8], Markus Tschurtschenthaler[1-3], Benjamin Walter[4], Sören A. Weidemann[1-3], Regina Pietsch [1-3], Angelika Schnieke[10], Roland M. Schmid[4], Maria S. Robles[5], Geoffroy Andrieux[11,12], Melanie Boerries[11,12], Roland Rad[3,6,7], Günter Schneider[1-3,13] and Dieter Saur[1-4*]

Supplementary Figures S1-S6

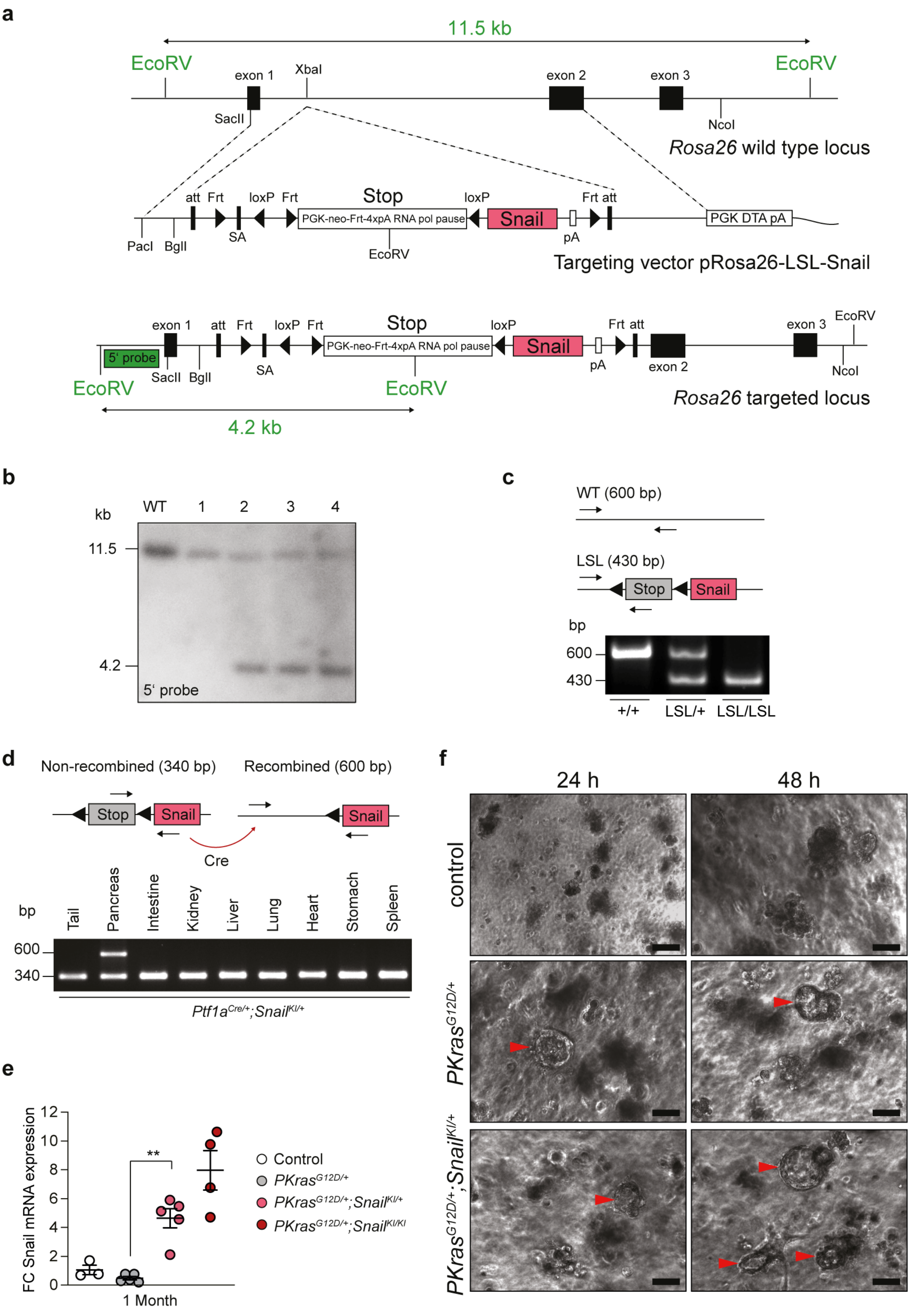

Supplementary Figure S1. Generation of a Cre-activatable Snail expression model.

(a) *Rosa26* targeting. From top to bottom, diagrams of: *Rosa26* wild-type locus; the *Rosa26* targeting vector with the lox-stop-lox (LSL) silenced *Snail* expression cassette; the targeted *Rosa26* locus. Restriction sites, location of the 5' probe, the exon structure of the *Rosa26* locus and sizes of DNA fragments are indicated. pA, polyadenylation sites; att, Gateway cloning sites; Frt, Frt recombination sites. **(b)** Southern blot analysis of DNA from wild type (WT, n=1) and targeted ES cells (1–4, n=16 in total) after EcoRV digestion. The expected band for the wild-type allele is 11.5 kb and for the targeted allele 4.2 kb. **(c)** Genotyping strategy. PCR analysis of DNA from wild-type (WT), heterozygous (LSL/+) and homozygous (LSL/LSL) *LSL-Rosa26*^{Snail} knock-in mice with retained stop cassette (n=3 per genotype). Sizes of WT and mutant PCR products are indicated. **(d)** Upper panel: Strategy to activate Snail expression by Cre-mediated excision of the LSL cassette and to detect the non-recombined and recombined LSL cassette by genotyping PCR. Lower panel: PCR analysis of DNA extracted from indicated tissues of *Ptf1a*$^{Cre/+}$;*LSL-R26*$^{Snail/+}$ knock-in mice (termed *Ptf1a*$^{Cre/+}$;*Snail*$^{KI/+}$) (n=3). Sizes of PCR products are indicated. **(e)** qRT-PCR analysis of *Snail* mRNA expression normalized to Cyclophilin A in pancreata of 1-month-old wild-type control (n=3), *Ptf1a*$^{Cre/+}$;*LSL-Kras*$^{G12D/+}$ (*PKras*$^{G12D/+}$, n=5), *Ptf1a*$^{Cre/+}$;*LSL-Kras*$^{G12D/+}$*;LSL-R26*$^{Snail/+}$ (*PKras*$^{G12D/+}$*;Snail*$^{KI/+}$, n=6) *and Ptf1a*$^{Cre/+}$;*LSL-Kras*$^{G12D/+}$*;lSL-R26*$^{Snail/Snail}$ (*PKras*$^{G12D/+}$*;Snail*$^{KI/KI}$, n=4) mice; mean ±SEM, **p=0.0029, unpaired two-tailed *t*-test with Welch's correction). Note: One outlier in the *PKras*$^{G12D/+}$*;Snail*$^{KI/+}$ cohort that differed significantly from the other observations, has been removed from the analysis (see panel S1e of Source Data file for outlier definition). FC, fold change. **(f)** Representative photomicrographs of ductal structures of the indicated genotypes formed in acinar explants cultured for 24h and 48h in a collagen layer (n=4 explants per genotype). Red arrowheads indicate ductal structures. Scale bars, 50µm. Source data of Supplementary Figure S1 are provided in the Source Data file.

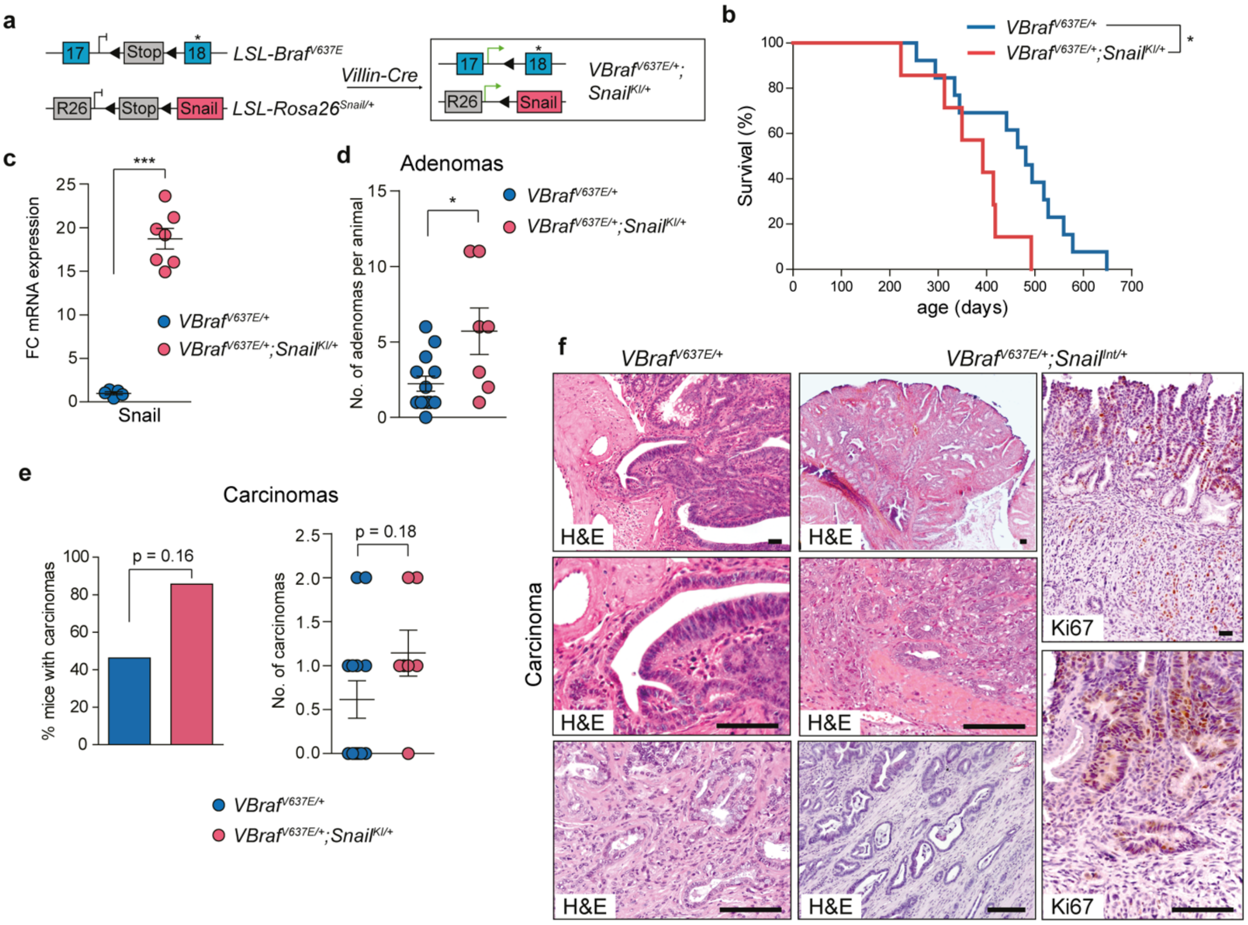

Supplementary Figure S2. Aberrant SNAIL expression promotes BRAF^{V637E}-driven intestinal cancer progression.

(a) Strategy to aberrantly express Snail in intestinal epithelium in the *Villin-Cre;LSL-Braf*$^{V637E/+}$ model (termed *VBraf*$^{V637E/+}$) of serrated intestinal cancer. **(b)** Kaplan-Meier survival curves of *VBraf*$^{V637E/+}$ (n=13, median survival 481 days) and *VBraf*$^{V637E/+}$*;Snail*$^{KI/+}$ (n=7, median survival 392 days) mice. *p=0.0321, log-rank test. **(c)** qRT-PCR analysis of Snail mRNA expression normalized to Cyclophilin A in the intestine of *VBraf*$^{V637E/+}$ (n=5) and *VBraf*$^{V637E/+}$*;Snail*$^{KI/+}$ endpoint mice (n=7). Mean ± SEM, **p<0.0001, unpaired two-tailed *t*-test with Welch's correction; FC, fold change. **(d)** Number of adenomas in the intestine of *VBraf*$^{V637E/+}$ (n=13) and *VBraf*$^{V637E/+}$*;Snail*$^{KI/+}$ (n=7) endpoint mice. Mean ±SEM, *p=0.0368, Mann-Whitney two-tailed test. **(e)** Percentage of carcinoma-bearing mice (left panel) and number of carcinomas (right panel) in the intestine of *VBraf*$^{V637E/+}$ (n=13) and *VBraf*$^{V637E/+}$*;Snail*$^{KI/+}$ (n=7) endpoint mice. Left panel by two-tailed Fisher's exact test; right panel by Mann-Whitney two-tailed test; p-values are indicated (not significant); mean ± SEM. **(f)** Representative H&E and Ki67 staining of intestinal carcinoma in *vBraf*$^{V637E/+}$ (n=6) and *VBraf*$^{V637E/+}$*;Snail*$^{KI/+}$ (n=6) mice. Scale bars, 50 µm. Source data of Supplementary Figure S2 are provided in the Source Data file.

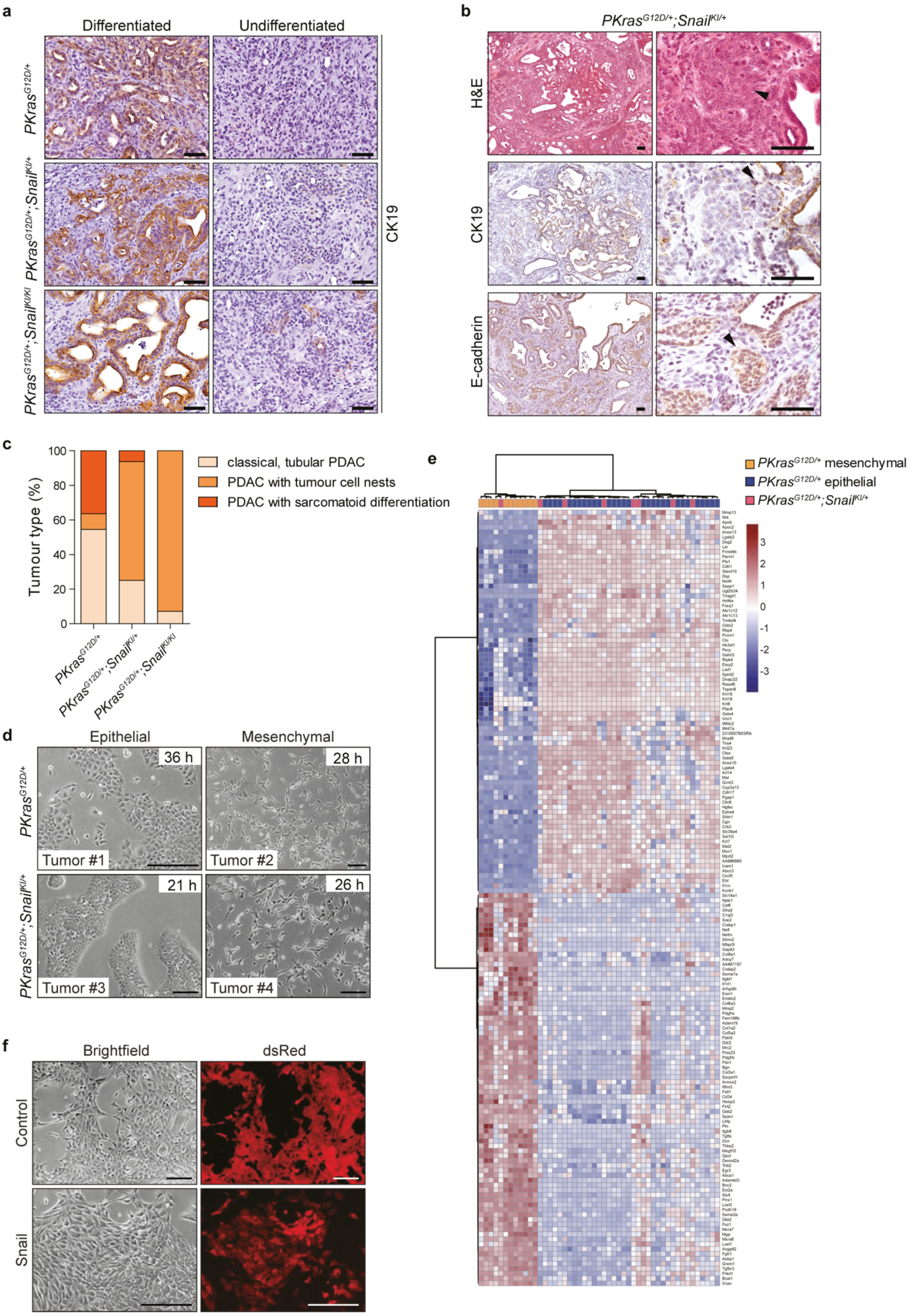

Supplementary Figure S3. Aberrant SNAIL expression induces PDAC with epithelial and mesenchymal differentiation.

(a) Representative images of CK19-stained PDAC sections of endpoint mice with indicated genotypes (n=6 per genotype). Note the presence of differentiated and undifferentiated tumours in all genotypes. Scale bars, 50 µm. **(b)** Representative H&E, CK19 and E-cadherin staining of PDAC sections of *PKras*$^{G12D/+}$*;Snail*$^{KI/+}$ mice (n=3). Black arrowheads indicate areas of tumour cells with solid nested growth. Scale bars, 50 µm. **(c)** Percentage of tumours with classical tubular PDAC, sarcomatoid differentiation and solid nested tumour cell growth in *PKras*$^{G12D/+}$ (n=11), *PKras*$^{G12D/+}$*;snail*$^{KI/+}$ (n=16) and *PKras*$^{G12D/+}$*;Snail*$^{KI/KI}$ mice (n=14). **(d)** Representative brightfield images of 2D cultured primary murine *PKras*$^{G12D/+}$*;Snail*$^{KI/+}$ (n=3) and *PKras*$^{G12D/+}$*;Snail*$^{KI/+}$ (n=3) PDAC cells with an epithelial (left) and mesenchymal (right) morphology. The cell doubling time in hours (h) is indicated in the upper right corner. Scale bars, 50 µm. **(e)** Heatmap showing the most significant >2-fold differentially regulated genes between mesenchymal and epithelial PDAC cell lines derived from primary tumours, circulating tumor cells in the blood and metastases from the *PKras*$^{G12D/+}$ model with and without *Trp53* mutation (n=41). Hierarchical clustering was performed including *PKras*$^{G12D/+}$*;Snail*^{KI} cell lines (n=8) with and without *Trp53* mutation, which are depicted in red. Mesenchymal *PKras*$^{G12D/+}$ cell lines with and without *Trp53* mutation are indicated in yellow, epithelial *PKras*$^{G12D/+}$ cell lines with and without *Trp53* mutation are depicted in blue. The colour code (top right) shows the standardized gene expression value (z-score). **(f)** Representative brightfield and fluorescent images of epithelial PDAC cells transduced with a retroviral Snail/dsRed expression vector (RCAS-TVA system) show epithelial morphology (n=3 per condition). Scale bars, 50 µm. dsRed fluorescence indicates successful retroviral transduction. Source data of Supplementary Figure S3 are provided in the Source Data file.

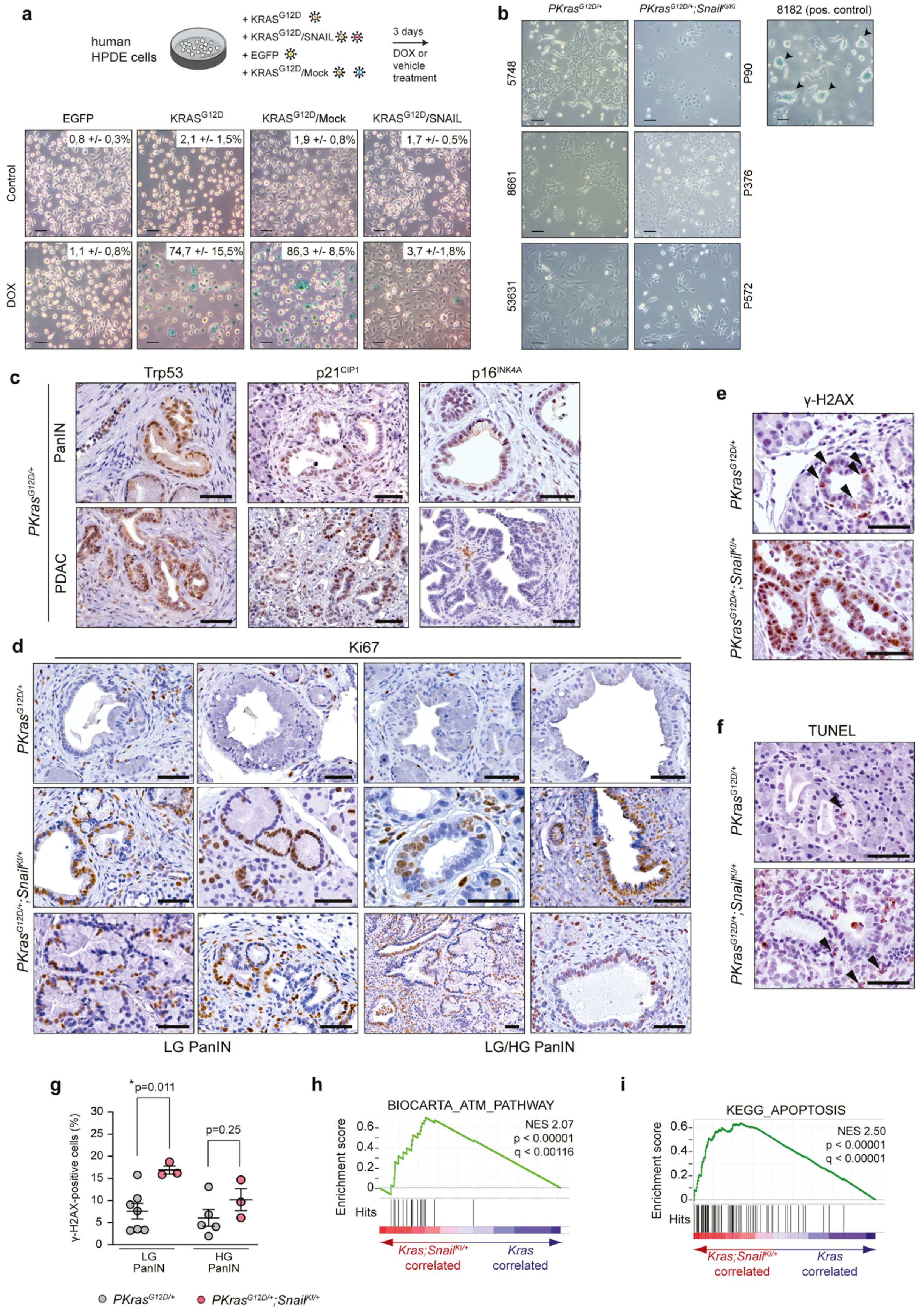

Supplementary Figure S4. SNAIL bypasses oncogenic Kras-induced senescence and increases DNA damage and apoptosis in PanIN lesions.

(**a**) Scheme of the strategy utilized for the doxycycline-regulated activation of EGFP, *$KRAS^{G12D}$* or *$KRAS^{G12D}$+SNAIL* in Human Pancreatic Duct Epithelial (HPDE) cells (upper panel). HPDE cells were transduced with lentiviral constructs and treated with doxycycline for 3 days. Lower panel: Representative images of SA-β-Gal stained HPDE cells after doxycycline-induced (100 ng ml^{-1}) activation of GFP, *$KRAS^{G12D}$* (+mock). n=3 independent experiments. The percentage of SA-β-gal^{+} cells is indicated in the upper right corner. Scale bars, 10μm. (**b**) Representative images of SA-β-Gal staining of primary low-passaged PDAC cell lines from *$PKras^{G12D/+}$* (n=3) and *$PKras^{G12D/+};Snail^{KI/KI}$* (n=3) mice. $LacZ^{+}$ PDAC cells were used as positive control (n=1). Scale bars, 20μm. **(c)** Immunohistochemical Trp53, $p21^{CIP1}$ and $p16^{INK4A}$ stainings of PanINs and PDAC of *$PKras^{G12D/+}$* animals (n=3 each). Please note that depending on the route of tumour evolution the tumour suppressor genes Trp53, $p21^{CIP1}$ and $p16^{INK4A}$ are lost or stay intact[32]. Therefore, the presented images are not representative for all *$PKras^{G12D/+}$* tumours, which show diverse routes of tumour evolution[32]. (**d**) Representative images of Ki67 staining of PanIN lesions of *$PKras^{G12D}$* (n=6) and *$PKras^{G12D/+};Snail^{KI/+}$* (n=8) mice. Scale bars, 50μm. (**e**) Immunohistochemical γ-H2AX staining of PanIN lesions of *$PKras^{G12D}$* (n=7) and *$PKras^{G12D/+};Snail^{KI/+}$* (n=3) mice. Scale bars, 50μm. (**f**) TUNEL staining of PanIN lesions of *$PKras^{G12D}$* (n=7) and *$PKras^{G12D/+};Snail^{KI/+}$* (n=3) mice. Scale bars, 50μm. **(g)** Percentage of γ-H2AX positive cells in PanIN lesions of *$PKras^{G12D/+};Snail^{KI/+}$* (n=3) and *$PKras^{G12D/+}$* (n=7) mice. Mean ±SD, *p=0.0113, unpaired two-tailed *t*-test. **(h, i)** Gene-set enrichment analysis (GSEA) using mRNA expression profiles of *$PKras^{G12D/+};Snail^{KI/+}$* (red) and *$PKras^{G12D/+}$* (blue) pancreata of 1-month-old mice (n=2 per genotype) computed and corrected for multiple testing using the Benjamini–Hochberg procedure (for statistical details, see methods section) showing significant enrichment of Biocarta ATM pathway (Normalized Enrichment Score: 2.07; Nominal p-value:<0.00001; False Discovery Rate (FDR) q-value:<0.00116) (**h**) and KEGG Apoptosis (Normalized Enrichment Score: 2.5; Nominal p-value:<0.00001; False Discovery Rate (FDR) q-value:<0.00001) genes (**i**).

LG, low grade; HG, high grade. Source data of Supplementary Figure S4 are provided in the Source Data file.

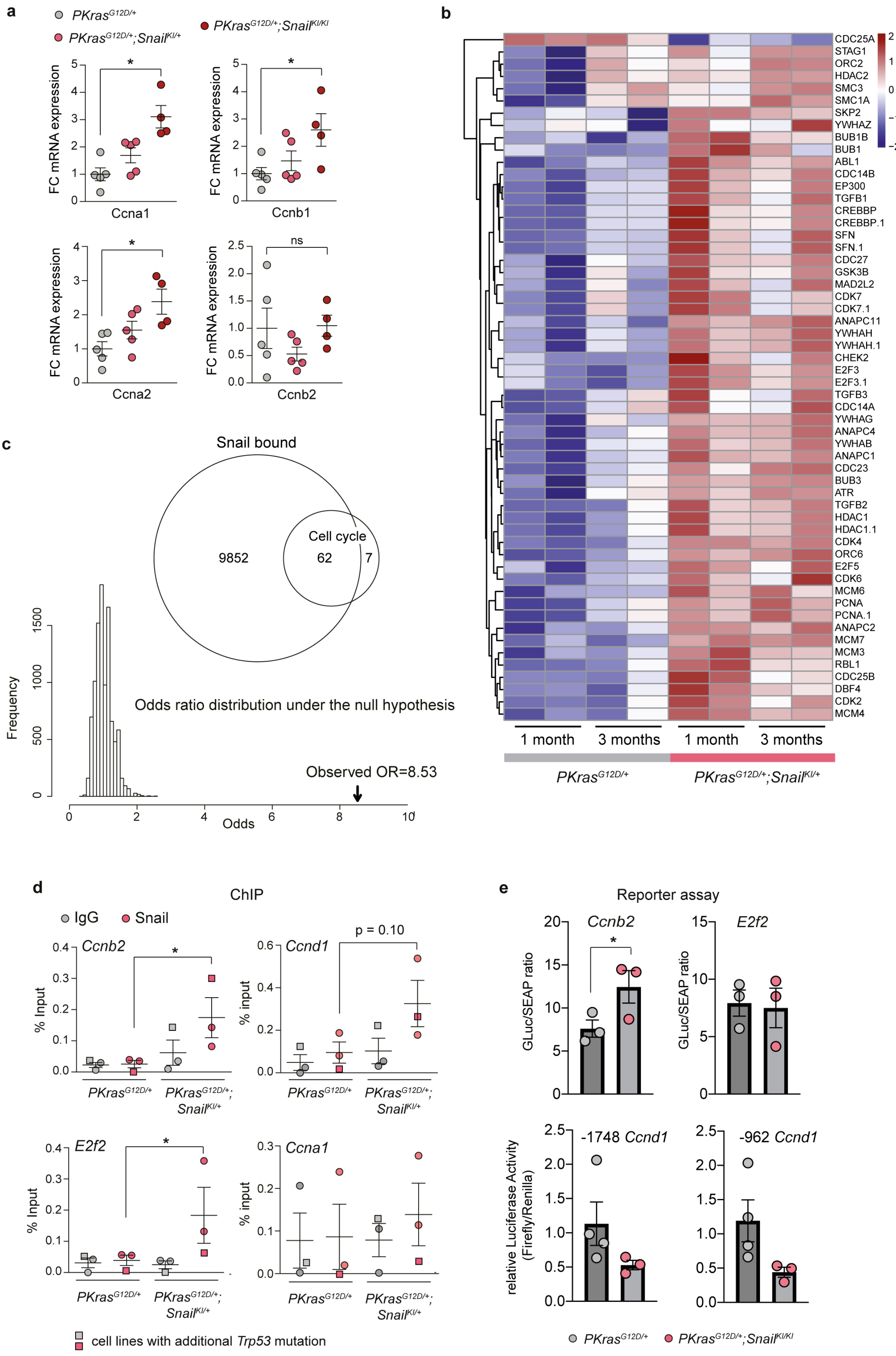
a
PKras$^{G12D/+}$
PKras$^{G12D/+}$;Snail$^{KI/+}$
PKras$^{G12D/+}$;Snail$^{KI/KI}$
FC mRNA expression
Ccna1
Ccnb1
Ccna2
Ccnb2
*
ns
b
CDC25A
STAG1
ORC2
HDAC2
SMC3
SMC1A
SKP2
YWHAZ
BUB1B
BUB1
ABL1
CDC14B
EP300
TGFB1
CREBBP
CREBBP.1
SFN
SFN.1
CDC27
GSK3B
MAD2L2
CDK7
CDK7.1
ANAPC11
YWHAH
YWHAH.1
CHEK2
E2F3
E2F3.1
TGFB3
CDC14A
YWHAG
ANAPC4
YWHAB
ANAPC1
CDC23
BUB3
ATR
TGFB2
HDAC1
HDAC1.1
CDK4
ORC6
E2F5
CDK6
MCM6
PCNA
PCNA.1
ANAPC2
MCM7
MCM3
RBL1
CDC25B
DBF4
CDK2
MCM4
1 month
3 months
1 month
3 months
PKras$^{G12D/+}$
PKras$^{G12D/+}$;Snail$^{KI/+}$
c
Snail bound
Cell cycle
9852
62
7
Frequency
Odds ratio distribution under the null hypothesis
Observed OR=8.53
Odds
d
ChIP
IgG
Snail
Ccnb2
Ccnd1
E2f2
Ccna1
% Input
% input
p = 0.10
*
PKras$^{G12D/+}$
PKras$^{G12D/+}$; Snail$^{KI/+}$
cell lines with additional Trp53 mutation
e
Reporter assay
Ccnb2
E2f2
GLuc/SEAP ratio
-1748 Ccnd1
-962 Ccnd1
relative Luciferase Activity (Firefly/Renilla)
PKras$^{G12D/+}$
PKras$^{G12D/+}$;Snail$^{KI/KI}$

Supplementary Figure S5. SNAIL directly binds to E-boxes of cell cycle regulators to increase their expression.

(a) qRT-PCR of mRNA expression of the indicated cyclins normalized to Cyclophilin A from pancreatic tissue of 1-month-old *PKras*$^{G12D/+}$ (n=5), *PKras*$^{G12D/+}$*;Snail*$^{KI/+}$ (n=5) and *PKras*$^{G12D/+}$*;Snail*$^{KI/KI}$ mice (n=4). Mean ±SEM, ns, not significant, *p=0.0159 (Ccna1 and Ccna2), *p=0.0317 (Ccnb1), p=0.7778 (Ccnb2), Mann-Whitney two-tailed test. FC, fold change. **(b)** Heatmap showing significantly regulated genes computed and corrected for multiple testing using the Benjamini–Hochberg procedure (for statistical details, please see methods section) involved in cell cycle regulation of *PKras*$^{G12D/+}$ (n=4) versus *Pkras*$^{G12D/+}$*;Snail*$^{KI/+}$ (n=4) pancreata (p-value <0.05 and q-value <0.05) from KEGG_CELL_CYCLE gene set. Colour represents standardized gene expression value (z-score). **(c)** Upper panel: Venn diagram showing overlap of (1) significantly enriched KEGG cell cycle genes in pancreas of 1-month-old *PKras*$^{G12D/+}$*;Snail*$^{KI/+}$ mice (n=2) computed and corrected for multiple testing using the Benjamini–Hochberg procedure (for statistical details, please see methods section) and (2) SNAIL-bound genes discovered by genome wide SNAIL-binding assay (ChIP-seq) in a previous study[11]. SNAIL-bound genes are defined as genes bearing a sequence bound by SNAIL within ± 1kb from the transcription start site. Lower panel: odds ratio (OR) of annotating the significantly enriched KEGG cell cycle genes to SNAIL-bound genes is significantly greater compared to that was calculated by replacing SNAIL-bound genes with randomly selected 9914 genes, non-parametric two-tailed Permutation test, p<0.001. **(d)** Chromatin immunoprecipitation (ChIP) of SNAIL binding to E-boxes of indicated promoters in *PKras*$^{g12D/+}$ (n=3) and *PKras*$^{G12D/+}$*;Snail*$^{KI/+}$ (n=3) PDAC cell lines ±*Trp53* mutation as indicated. %input calculation; IgG, negative control. Mean ±SEM. *p=0.05, Mann-Whitney one-tailed test. Note: Depicted genes including those shown in Fig. 7g (*Ccnb1, Ccnb2, Ccnd1, E2f2* and *E2f3*) are present in the SNAIL-bound fraction of cell cycle genes of the ChIP-seq data of panel (c). (**e**) *Ccnb2, Ccnd1* (-1748 and -962 site) and *E2f2* promoter activity in PDAC cells from *PKras*$^{g12D/+}$ (n=3) and *PKras*$^{G12D/+}$*;Snail*$^{KI/KI}$ (n=3) mice (three independent experiments). Mean ±SEM, *p=0.0423, unpaired one-tailed Student's *t*-test. Source data of Supplementary Figure S5 are provided in the Source Data file.

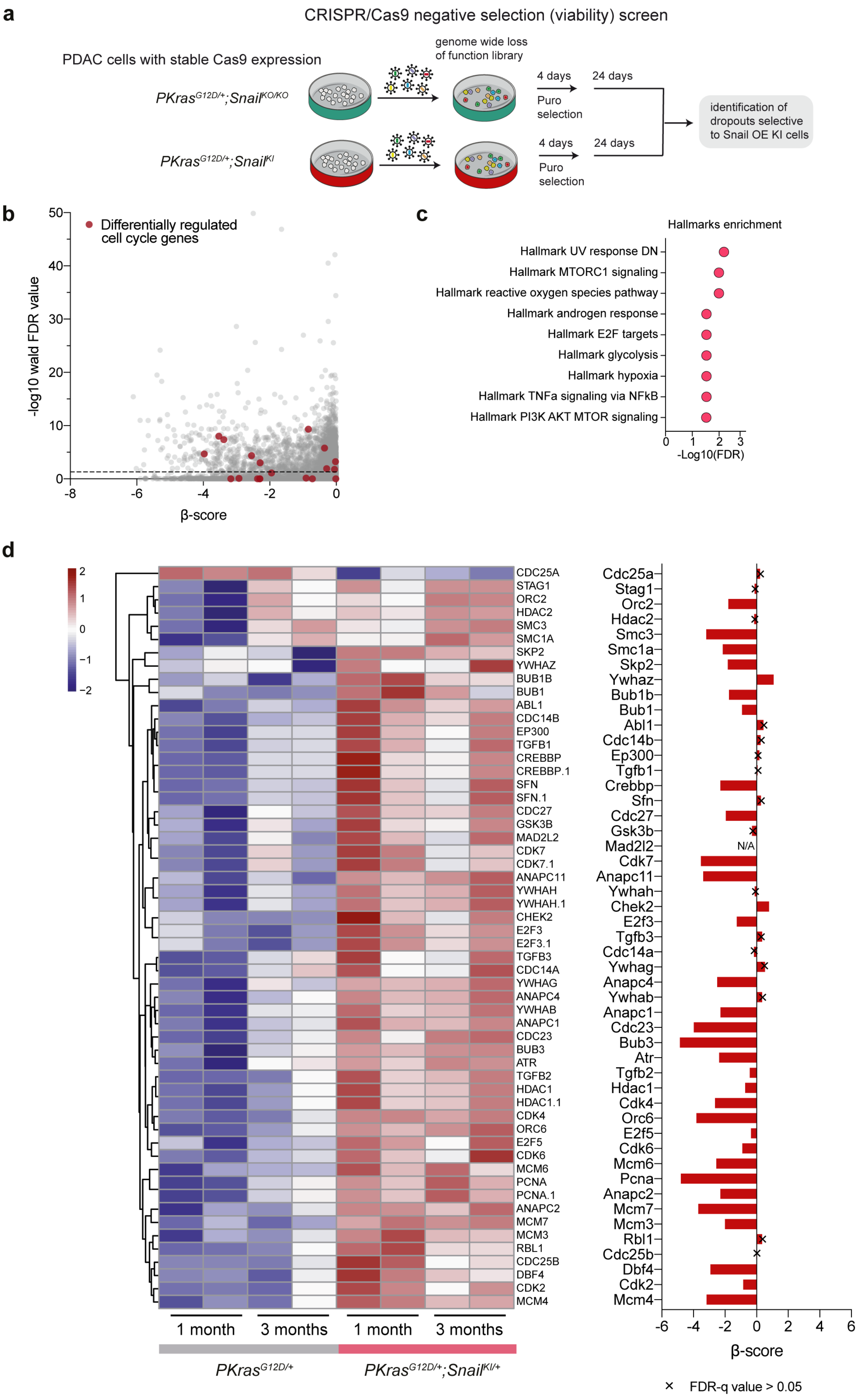
a
CRISPR/Cas9 negative selection (viability) screen
PDAC cells with stable Cas9 expression
genome wide loss of function library
PKras^G12D/+;Snail^KO/KO
PKras^G12D/+;Snail^KI
4 days
24 days
Puro selection
identification of dropouts selective to Snail OE KI cells
b
Differentially regulated cell cycle genes
-log10 wald FDR value
β-score
c
Hallmarks enrichment
Hallmark UV response DN
Hallmark MTORC1 signaling
Hallmark reactive oxygen species pathway
Hallmark androgen response
Hallmark E2F targets
Hallmark glycolysis
Hallmark hypoxia
Hallmark TNFa signaling via NFkB
Hallmark PI3K AKT MTOR signaling
-Log10(FDR)
d
CDC25A STAG1 ORC2 HDAC2 SMC3 SMC1A SKP2 YWHAZ BUB1B BUB1 ABL1 CDC14B EP300 TGFB1 CREBBP CREBBP.1 SFN SFN.1 CDC27 GSK3B MAD2L2 CDK7 CDK7.1 ANAPC11 YWHAH YWHAH.1 CHEK2 E2F3 E2F3.1 TGFB3 CDC14A YWHAG ANAPC4 YWHAB ANAPC1 CDC23 BUB3 ATR TGFB2 HDAC1 HDAC1.1 CDK4 ORC6 E2F5 CDK6 MCM6 PCNA PCNA.1 ANAPC2 MCM7 MCM3 RBL1 CDC25B DBF4 CDK2 MCM4
1 month
3 months
PKras^G12D/+
PKras^G12D/+;Snail^KI/+
Cdc25a Stag1 Orc2 Hdac2 Smc3 Smc1a Skp2 Ywhaz Bub1b Bub1 Abl1 Cdc14b Ep300 Tgfb1 Crebbp Sfn Cdc27 Gsk3b Mad2l2 Cdk7 Anapc11 Ywhah Chek2 E2f3 Tgfb3 Cdc14a Ywhag Anapc4 Ywhab Anapc1 Cdc23 Bub3 Atr Tgfb2 Hdac1 Cdk4 Orc6 E2f5 Cdk6 Mcm6 Pcna Anapc2 Mcm7 Mcm3 Rbl1 Cdc25b Dbf4 Cdk2 Mcm4
N/A
β-score
× FDR-q value > 0.05

Supplementary Figure S6. Genome-wide CRISPR negative selection screens identify selective dependencies of Snail-driven PDAC cells on specific cell cycle regulators.

(a) Schematic representation of the genome-wide CRISPR/Cas9 negative selection screen as shown in Fig. 7j. **(b)** Volcano plot representing the negative β-scores of the CRISPR screen performed in PDAC cells from $PKras^{G12D/+};Snail^{KO/KO}$, $PKras^{G12D/+};Snail^{KI/+}$, $PKras^{G12D/+};Snail^{KI/KI}$ mice (n=4). Red dots denote differentially hit genes involved in cell cycle. **(c)** Enrichment analysis using the Hallmarks gene-set on the MSigDB portal. Only genes with an FDR ≤ 0.05 and a difference in β score ($Snail^{Ki}$ overexpression (OE) - $Snail^{KO}$ knock-out (KO) cells) < -1 were used for the analysis. **(d)** Integration of gene expression heatmap of Supplementary Fig. S5b showing significantly regulated cell cycle related genes of $PKras^{G12D/+}$ and $PKras^{G12D/+};Snail^{KI/+}$ mice (p-value < 0.05 and q-value < 0.05) from KEGG_CELL_CYCLE gene set (left panel; for statistical details, please see methods section) and β-scores of PDAC cell lines from the genome-wide CRISPR/Cas9 negative selection screen, calculated with the MAGeCK pipeline (right panel; for statistical details, please see methods section). Colour in the heatmap (left panel) represents standardized gene expression value (z-score). Genes of the right panel showing an FDR-q value >0.05 are marked with a X on the bar.
