# Supplementary material for: Non-canonical functions of SNAIL drive context-specific cancer progression
Source: Nat Commun. 2023 Mar 7;14:1201. doi: 10.1038/s41467-023-36505-0 (PMC9992512; doi:10.1038/s41467-023-36505-0)
Supplement: Supplementary file 2 — Description of Additional Supplementary Files [file 41467_2023_36505_MOESM2_ESM.pdf]

Description of Additional Supplementary Files

File Name: Supplementary Data 1
Description: List of proteins identified by ChIP-MS

File Name: Supplementary Data 2
Description: List of genes of genome wide CRISPR/Cas9 negative selection screen
