# Supplementary material for: Non-canonical functions of SNAIL drive context-specific cancer progression
Source: Nat Commun. 2023 Mar 7;14:1201. doi: 10.1038/s41467-023-36505-0 (PMC9992512; doi:10.1038/s41467-023-36505-0)
Supplement: Supplementary file 5 — Reporting Summary [file 41467_2023_36505_MOESM5_ESM.pdf]

nature portfolio

Corresponding author(s): Dieter Saur

Last updated by author(s): Dec 11, 2022

Reporting Summary

Nature Portfolio wishes to improve the reproducibility of the work that we publish. This form provides structure for consistency and transparency in reporting. For further information on Nature Portfolio policies, see our Editorial Policies and the Editorial Policy Checklist.

Statistics

For all statistical analyses, confirm that the following items are present in the figure legend, table legend, main text, or Methods section.

n/a	Confirmed	
☐	☒	The exact sample size (*n*) for each experimental group/condition, given as a discrete number and unit of measurement
☐	☒	A statement on whether measurements were taken from distinct samples or whether the same sample was measured repeatedly
☐	☒	The statistical test(s) used AND whether they are one- or two-sided<br>*Only common tests should be described solely by name; describe more complex techniques in the Methods section.*
☒	☐	A description of all covariates tested
☐	☒	A description of any assumptions or corrections, such as tests of normality and adjustment for multiple comparisons
☐	☒	A full description of the statistical parameters including central tendency (e.g. means) or other basic estimates (e.g. regression coefficient) AND variation (e.g. standard deviation) or associated estimates of uncertainty (e.g. confidence intervals)
☐	☒	For null hypothesis testing, the test statistic (e.g. *F*, *t*, *r*) with confidence intervals, effect sizes, degrees of freedom and *P* value noted<br>*Give P values as exact values whenever suitable.*
☒	☐	For Bayesian analysis, information on the choice of priors and Markov chain Monte Carlo settings
☐	☒	For hierarchical and complex designs, identification of the appropriate level for tests and full reporting of outcomes
☐	☒	Estimates of effect sizes (e.g. Cohen's *d*, Pearson's *r*), indicating how they were calculated

Our web collection on statistics for biologists contains articles on many of the points above.

Software and code

Policy information about availability of computer code

Data collection

Microarray data were acquired using an Affymetrix Scanner 3000 7G and the Affymetrix GeneChip Command Console Software (AGCC). For mRNA analysis of PDAC cell lines data were collected using the GeneChip mouse genome 430 2.0 array chip (Affymetrix). For mRNA analysis of pancreatic and PDAC tissues the Affymetrix GeneChip Mouse Gene 1.0 ST Array was used. Expression intensity of each gene was determined by using the Affymetrix Microarray Analysis Suite (MAS) 5.0 software.

Pictures of macroscopic view of organs, cell lines and acinar explant were acquired with AxioVision Rel 4.8.

Histology pictures were acquired by AxioVision Rel 4.8 and Aperio ImageScope v12.3.3.

qPCR data was collected with the StepOnePlus real time PCR system (Applied Biosystems), by using the StepOne Software v2.3.

The sgRNA libraries of the CRISPR/Cas9 screens were sequenced using a Illumina NextSeq 500.

DNA gel data was collected by the Gel Doc™ XR+ system.

Western blot data was collected by the Odyssey infrared imaging system using the Odyssey Software V1.2 (Li-Cor Biosciences).

Southern blot data was collected by the Amersham Hyperprocessor automatic film processor (Amersham Biosciences).

LC-MS/MS data were acquired via a Q-Exactive HF-X hybrid quadrupole-orbitrap mass spectrometer (Thermo Fisher Scientific) after peptide separation by high-performance liquid chromatography (nanoLC 1200, Thermo Fisher Scientific).

Promoter reporter assays data were collected with CLARIOstar microplate reader (BMG Labtech GmbH).

Additional information are reported in the methods section of the manuscript.

Data analysis

Statistical analysis were conducted with the following softwares:

For gene expression analysis R (v3.1.2), Bioconductor (v3.0), Limma (v3.22.0), GSEA (v3.0), MSigDB (v6.2) were used.

For mass spectrometry data analysis MaxQuant (v1.6.1.0), Perseus (v1.6.7.0), R (v3.6.2) and MSigDB (v7.1) were employed.

For analysis of the CRISPR/Cas9 screen MAGeCK v0.5.9.4 and MSigDB (v7.1) were used.

For analysis of the human primary PDAC cohort R version 4.2.12, Trimmomatic (v2.38), STAR (v2.6.0) were used.

For all other analyses Graphpad Prism v5 and v8, Microsoft excel (v16.65).

Additional information are reported in the methods section of the manuscript.

For manuscripts utilizing custom algorithms or software that are central to the research but not yet described in published literature, software must be made available to editors and reviewers. We strongly encourage code deposition in a community repository (e.g. GitHub). See the Nature Portfolio guidelines for submitting code & software for further information.

Data

Policy information about availability of data

All manuscripts must include a data availability statement. This statement should provide the following information, where applicable:
- Accession codes, unique identifiers, or web links for publicly available datasets
- A description of any restrictions on data availability
- For clinical datasets or third party data, please ensure that the statement adheres to our policy

All microarray data generated in this study have been deposited in the ArrayExpress database (https://www.ebi.ac.uk/arrayexpress/) with accession # E-MTAB-8173 (https://www.ebi.ac.uk/biostudies/studies/E-MTAB-8173) and # E-MTAB-8174 (https://www.ebi.ac.uk/biostudies/studies/E-MTAB-8174).

Protein data generated by mass spectrometry have been deposited in the PRIDE database under accession code # PXD038726 (https://www.ebi.ac.uk/pride/archive/projects/PXD038726).

The ChIP-MS and genome wide CRISPR/Cas9 negative selection screen data generated in this study are provided in the Supplementary Data file 1 and 2, respectively.

Source data (raw and processed data) for all data presented in graphs are provided as source data with the paper.
Mice and cell lines are available from the corresponding author on request.

The database MSigDB (v6.2 and 7.1) was used in the manuscript. The gene sets utilized for analysis were Hallmarks and Reactome.

Field-specific reporting

Please select the one below that is the best fit for your research. If you are not sure, read the appropriate sections before making your selection.

☒ Life sciences ☐ Behavioural & social sciences ☐ Ecological, evolutionary & environmental sciences

For a reference copy of the document with all sections, see nature.com/documents/nr-reporting-summary-flat.pdf

Life sciences study design

All studies must disclose on these points even when the disclosure is negative.

Sample size	No statistical methods were used to predetermine sample size. The biologically relevant effects and sample sizes were chosen based on our previous published and unpublished data that have been statistically evaluated. Thus, the sample size of our current study is based on our experience in this area of research, as well as the advice from bio-statisticians.
Data exclusions	In Suppl. Fig 1e, one outlier in the PKrasG12D/+;SnailKI/+ cohort that differed significantly from the other observations, has been removed from the analysis (please see Source Data file, Supplementary Figure 1e, for detailed information on outlier definition). No other data were excluded from other datasets.
Replication	Our findings are reproduced by independent biological replicates as fully reported in the figure legends and the source data.
Randomization	For the experiments described in this manuscript randomization was not possible/appropriate. Genetically engineered animals developed the tumors spontaneously and we have not performed interventions. All models (tumors or tumor cells) have been characterized so that we did not randomly pick selected ones for further analysis.
Blinding	The processing of the samples and their analysis was performed in a blinded fashion (e.g., blinded to the genotype and the outcome).

Blinding | Moreover, experiments were analyzed by independent investigators.

Reporting for specific materials, systems and methods

We require information from authors about some types of materials, experimental systems and methods used in many studies. Here, indicate whether each material, system or method listed is relevant to your study. If you are not sure if a list item applies to your research, read the appropriate section before selecting a response.

Materials & experimental systems

n/a	Involved in the study
☐	☒ Antibodies
☐	☒ Eukaryotic cell lines
☒	☐ Palaeontology and archaeology
☐	☒ Animals and other organisms
☒	☐ Human research participants
☒	☐ Clinical data
☒	☐ Dual use research of concern

Methods

n/a	Involved in the study
☒	☐ ChIP-seq
☒	☐ Flow cytometry
☒	☐ MRI-based neuroimaging

Antibodies

Antibodies used

The following primary antibodies were used:
- For western blot: SNAIL (#3895, Cell Signaling Technology), E-cadherin (#610181, BD Biosciences), HSP90 (#sc-13119, Santa Cruz Biotechnology), TRP53 (#NCL-p53-CM5p, Novocastra/Leica Microsystems), p21CIP1 (#sc-397, Santa Cruz Biotechnology), β-Actin (#A5316, Sigma-Aldrich) and α-Tubulin (#T9026, Sigma-Aldrich).
- For histology and immunohistochemistry: Muc5a (#MS-145-P1, Neomarkers), Cytokeratin 19 (TROMA 3 Developmental Studies Hybridoma Bank), E-cadherin (#610181, BD Biosciences), Rabbit anti-Ki67 (#MA5-14520, SP6, ThermoFischer), γ-H2AX (#05-636, Millipore), Cleaved Caspase 3 (#9664, Cell Signaling Technology), BrdU (#MCA2060, AbD Serotec), pRB (#8516, Cell Signaling Technology), p16INK4A (#sc-1661, Santa Cruz Biotechnology), TRP53 (#NCL-p53-CM5p, Novocastra/Leica Microsystems), p21CIP1 (#sc-397, Santa Cruz Biotechnology), SNAIL (#3879, Cell Signaling Technology).
- For Immunocytochemistry: E-cadherin antibody (#AF748, R&D Systems) and donkey anti-goat Alexa Fluor® 488 (# A-11055, Invitrogen).
- For immunoprecipitation: SNAIL (#3879, Cell Signaling Technology), Normal Rabbit IgG (#2729, Cell Signaling Technology), Histone H3 (#2650, Cell Signaling Technology).

Validation

All antibodies used in this study were validated for the use in mouse samples by the manufacturers and adequate controls were included (positive and negative controls).

E-cadherin, Trp53, Snail, p16INK4A antibodies were validated with samples from the respective knock-out mice. All other antibodies were validated by cell type specific stainings and signal localization; antibodies for western blot were validated by band size.

Antibodies were validated in addition by the manufacturer for specific applications. Detailed information for the respective antibodies can be found on the manufacturer's website:

Muc5a (#MS-145-P1, Neomarkers): https://tools.thermofisher.com/content/sfs/brochures/D11647~.pdf

Cytokeratin 19 (TROMA 3 Developmental Studies Hybridoma Bank):
https://dshb.biology.uiowa.edu/core/media/media.nl?
id=1523553&c=571578&h=O22gYwGyD6-6QmGzY5i_W20gahwIwy1BpZJrZUXkQ2mjAtd1&_xt=.pdf

E-cadherin (#610181, BD Biosciences)
https://www.bdbiosciences.com/content/bdb/paths/generate-tds-document.de.610181.pdf

Rabbit anti-Ki67 (SP6) (# MA5-14520, ThermoFisher)
https://www.thermofisher.com/order/genome-database/dataSheetPdf?
producttype=antibody&productsubtype=antibody_primary&productId=MA5-14520&version=260

γ-H2AX (#05-636, Millipore)
https://www.merckmillipore.com/DE/de/product/Anti-phospho-Histone-H2A.X-Ser139-Antibody-clone-JBW301,MM_NF-05-636#

Cleaved Caspase 3 (#9664, Cell Signaling Technology)
https://www.cellsignal.de/datasheet.jsp?productId=9664&images=1&size=A4

BrdU (#MCA2060, AbD Serotec)
https://www.bio-rad-antibodies.com/static/datasheets/mca20/brdu-antibody-bu1-75-icr1-mca2060t.pdf

pRB (#8516, Cell Signaling Technology)
https://www.cellsignal.de/datasheet.jsp?productId=8516&images=1&size=A4

p16INK4A (#sc-1661, Santa Cruz Biotechnology)
https://datasheets.scbt.com/sc-1661.pdf

TRP53 (#NCL-p53-CM5p, Novocastra/Leica Microsystems)
https://shop.leicabiosystems.com/en-de/actions/ViewProductAttachment-OpenFile?LocaleId=en_US&DirectoryPath=SDSs&FileName=p53-cm5p-l.pdf&UnitName=LBS

p21CIP1 (#sc-397, Santa Cruz Biotechnology)
https://datasheets.scbt.com/sc-397.pdf

SNAIL (#3879, Cell Signaling Technology)
https://www.cellsignal.com/datasheet.jsp?productId=3879&images=1

E-cadherin antibody (1:80, #AF748, R&D Systems)
https://resources.rndsystems.com/pdfs/datasheets/af748.pdf?v=20221212&_ga=2.188075624.1180255560.1670872759-1618182263.1664975067

Donkey anti-Goat IgG Alexa Fluor™ 488 (Catalog # A-11055, Invitrogen)
https://www.thermofisher.com/order/genome-database/dataSheetPdf?producttype=antibody&productsubtype=antibody_secondary&productId=A-11055&version=260

Immunoprecipitation:
SNAIL (#3879, Cell Signaling Technology)
https://www.cellsignal.com/datasheet.jsp?productId=3879&images=1

Normal Rabbit IgG (#2729, Cell Signaling Technology)
https://www.cellsignal.de/datasheet.jsp?productId=2729&images=1&size=A4

Histone H3 (#2650, Cell Signaling Technology)
https://www.cellsignal.de/products/primary-antibodies/histone-h3-antibody-chip-formulated/2650

Eukaryotic cell lines

Policy information about cell lines

Cell line source(s)

Human PDAC cell lines were purchased from ATCC, DSMZ and Cell bank. All murine cell lines were self-generated from murine autochthonous primary pancreatic cancers, metastases or ascites in the lab.
Human PDAC cell lines:
Official Name (RRID Identifier) ATTC/DSMZ/Cell bank number
AsPC-1 (CVCL_0152) ATCC: CRL-1682
Capan-2 (CVCL_0026) ATCC: HTB-80
CFPAC-1 (CVCL_1119) ATCC: CRL-1918
DAN-G (CVCL_0243) DSMZ: ACC 249
HPAC (CVCL_3517) ATCC: CRL-2119
HPAF-II (CVCL_0313) ATCC: CRL-1997
Hs 766T (CVCL_0334) ATCC: HTB-134
HuP-T4 (CVCL_1300) DSMZ: ACC 223
IMIM-PC1 (CVCL_4061) https://www.cellosaurus.org/CVCL_4061
KP-4 (CVCL_1338) Cell bank: RCB1005 (https://cellbank.brc.riken.jp/cell_bank/CellInfo/?cellNo=RCB1005)
MIA PaCa-2 (CVCL_0428) ATCC: CRL-1420
Panc 02.03 (CVCL_1633) ATCC: CRL-2553
Panc 03.27 (CVCL_1635) ATCC: CRL-2549
Panc 04.03 (CVCL_1636) ATCC: CRL-2555
Panc 05.04 (CVCL_1637) ATCC: CRL-2557
Panc 08.13 (CVCL_1638) ATCC: CRL-2551
PANC-1 (CVCL_0480) ATCC: CRL-1469
Panc 10.05 (CVCL_1639) ATCC: CRL-2547
PaTu 8902 (CVCL_1845) DSMZ: ACC 179
PaTu 8988s (CVCL_1846) DSMZ: ACC 204
PL45 (CVCL_3567) ATCC: CRL-2558
PSN1 (CVCL_1644) ATCC: CRL-3211
SU.86.86 (CVCL_3881) ATCC: CRL-1837
SW1990 (CVCL_1723) ATCC: CRL-2172
YAPC (CVCL_1794) DSMZ: ACC 382

Human Pancreatic Duct Epithelial Cell Line (H6c7) (CVCL_0P38) Kerafast: ECA001-FP (https://www.kerafast.com/productgroup/531/human-pancreatic-duct-epithelial-cell-line-h6c7)

The avian TVA receptor positive chicken embryonic fibroblast cell line DF-1 (RRID:CVCL_0570) was obtained from ATCC (CRL-12203).

Authentication

All human cell lines were authenticated through STR or SNP profiling. All murine cell lines were re-genotyped and tested for recombination of the respective alleles. The chicken fibroblast cell line DF-1 was authenticated by genotyping PCR for presence of the avian TVA receptor.

Mycoplasma contamination

All cell lines were routinely checked for mycoplasma contamination and tested negative.

Commonly misidentified lines
(See ICLAC register)

No misidentified cell lines according to the ICLAC register were used in this study.

Animals and other organisms

Policy information about studies involving animals; ARRIVE guidelines recommended for reporting animal research

Laboratory animals

The ARRIVE guidelines were strictly followed. All genetically engineered mouse models were on a mixed C57Bl/6J;129S6/SvEv genetic background and interbred to obtain compound mutant mice of both sexes (male and female as indicated in the source data file). The animals were analyzed at an age between 18 and 1096 days as indicated in the figures and source data file. The animals, which were used for the analysis, were generated by crossing the following alleles: LSL-KrasG12D/+, Pdx1-Cre, Ptf1aCre/+, LSL-Trp53R172H/+, LSL-Rosa26TvailacZ/+, Cdkn2alox/+, p16Ink4a*/+, Villin-Cre, LSL-BrafV637E/+, Apclox/+, Cdh1lox/+, Snail fl/fl. These mice have been described previously and the LSL-Rosa26Snail allele was generated in this study.
Animals were housed under specific pathogen free conditions (SPF) in a dedicated facility, with a light-dark cycle of 12:12 hours, a relative air humidity between 45 and 65%, and a temperature between 20 and 24°C.

Wild animals

No wild animals were used.

Field-collected samples

No field-collected samples were used.

Ethics oversight

All animal studies were conducted in compliance with European guidelines for the care and use of laboratory animals and were approved by the Institutional Animal Care and Use Committees (IACUC) of the local authorities of Technische Universität München and the Regierung von Oberbayern.

Note that full information on the approval of the study protocol must also be provided in the manuscript.
