# Supplementary material for: Non-canonical functions of SNAIL drive context-specific cancer progression
Source: Nat Commun. 2023 Mar 7;14:1201. doi: 10.1038/s41467-023-36505-0 (PMC9992512; doi:10.1038/s41467-023-36505-0)

Figure 1

Panel 1b

SNAIL Western blot

PKras$^{G12D/+}$ PKras$^{G12D/+}$

MW ; Snail

SNAIL

30 kDa -

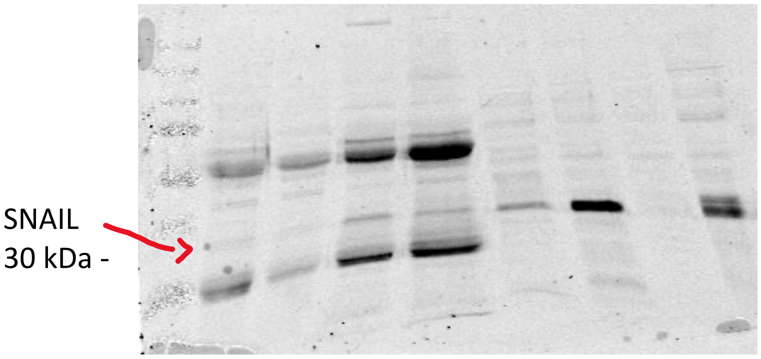

β actin Western blot

β-actin

40 kDa_

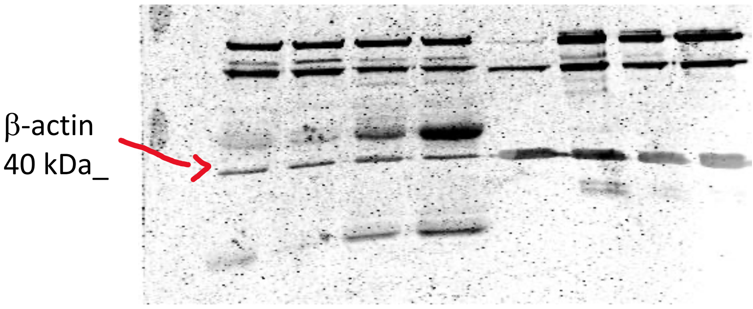

Figure 3

Panel 3c

E-cadherin Western blot

PKras$^{G12D/+}$

MW WT WT Snail$^{KI/+}$ Snail KI/KI MW

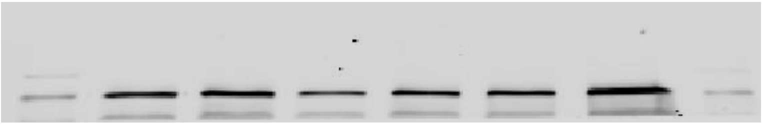

- 120 kDa

HSP 90 Western blot

PKras$^{G12D/+}$

MW WT WT Snail$^{KI/+}$ Snail KI/KI MW

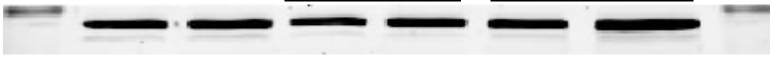

- 90 kDa

Figure 3

Panel 3h (right panel)

E-cadherin and β-actin Western blot

RCAS Snail

MW transduced

- +

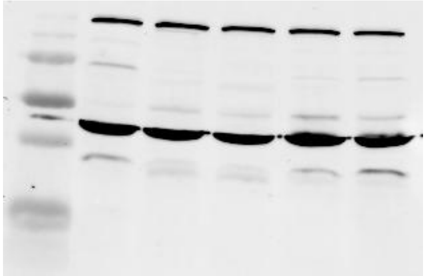

- 120 kDa (E-cadherin)

- 42 kDa (β-actin)

SNAIL Western blot

RCAS Snail

MW transduced

- +

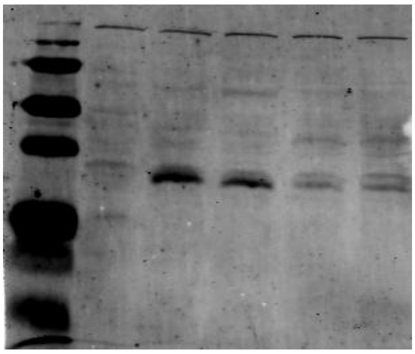

- 30 kDa (SNAIL)

Panel 3h (left panel)

E-cadherin and β-actin Western blot

Rosa26 Snail knock-in

MW KI/KI KI/+ KI/+

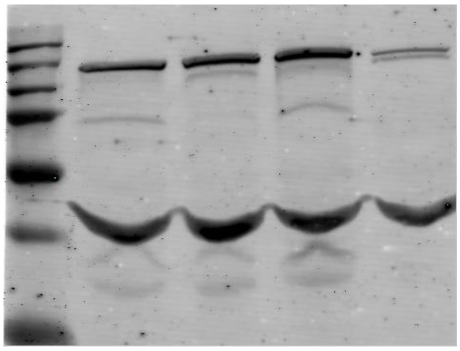

- 120 kDa (E-cadherin)

- 42 kDa (β-actin)

SNAIL Western blot

Rosa26 Snail knock-in

MW KI/KI KI/+ KI/+

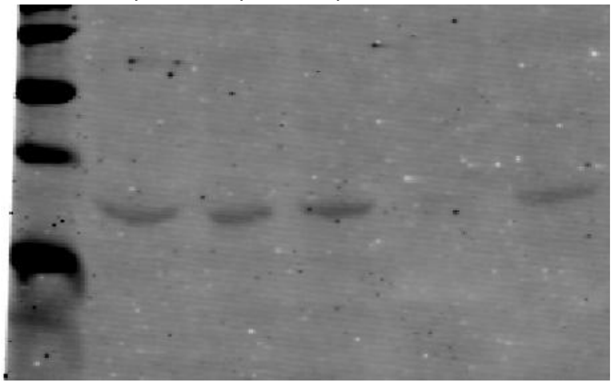

- 30 kDa (SNAIL)

Fig. 4

Panel 4a

Genotyping PCR of SNAIL knock-out alleles

MW cells tumor cells cells tumor cells

Kras Kras;Snail$^{KO/KO.}$ Kras Kras;Snail $^{KO/KO}$

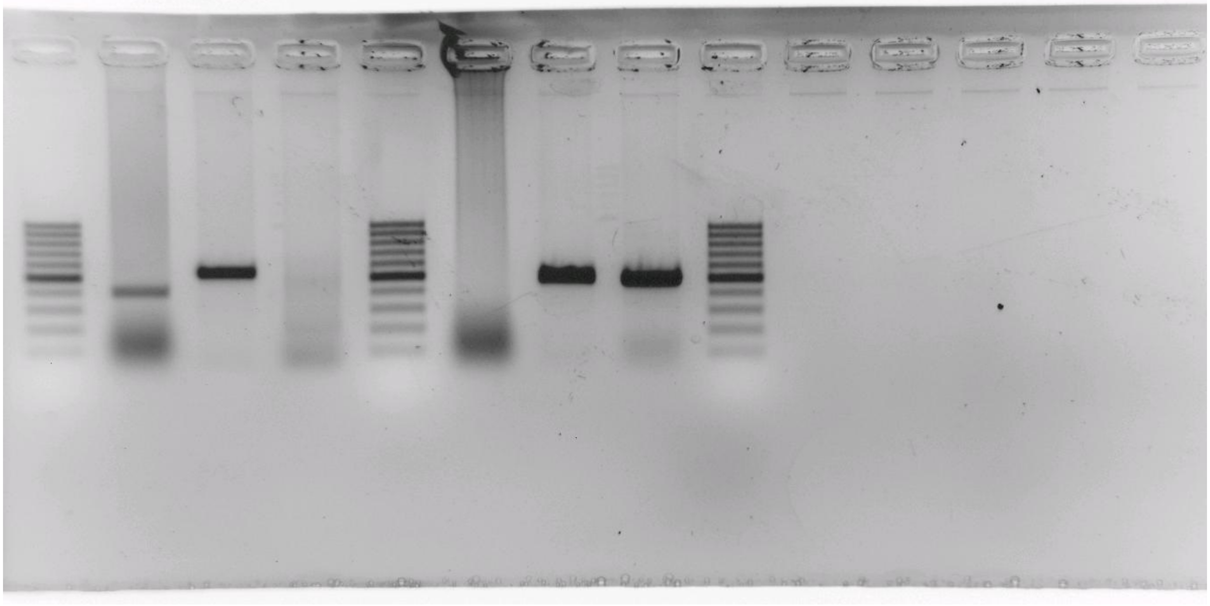

Figure 6

Panel 6c
TRP53 Western blot

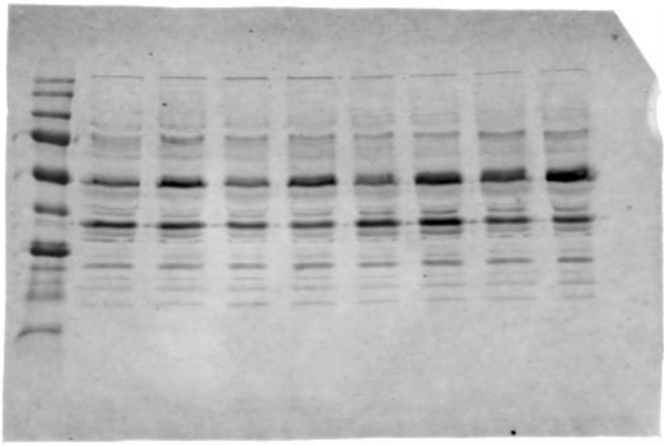

\- 53 kDa (TRP53

P21CIP1 Western blot

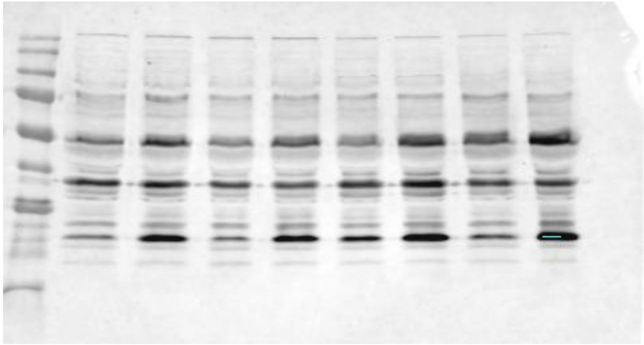

\- 21 kDa (p21)

α-Tubulin Western blot

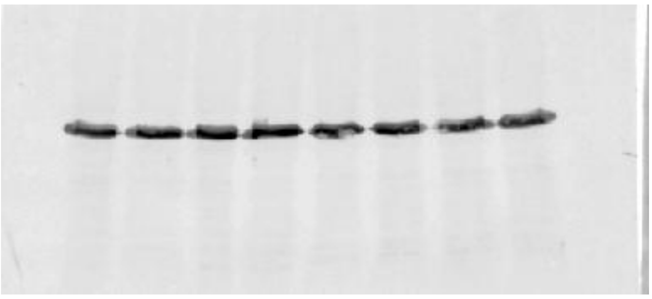

\- 55 kDa (α-Tubulin)

Figure 6

Panel 6j

P16^{Ink4a} genotyping PCR

PKras MW

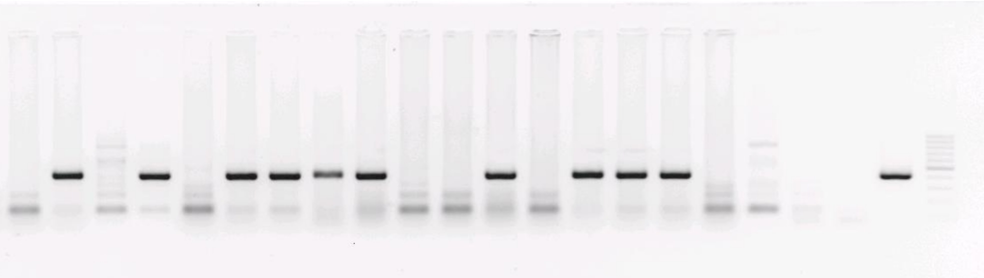

MW PKras PKras;Snail $^{KI/+}$ MW

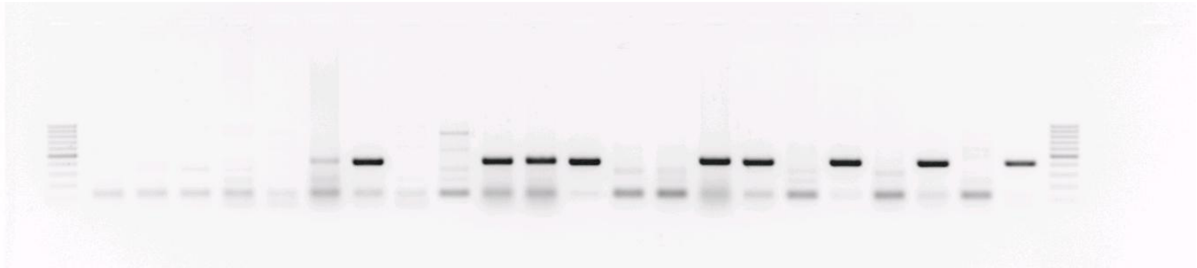

MW PKras;Snail $^{KI/KI}$ MW

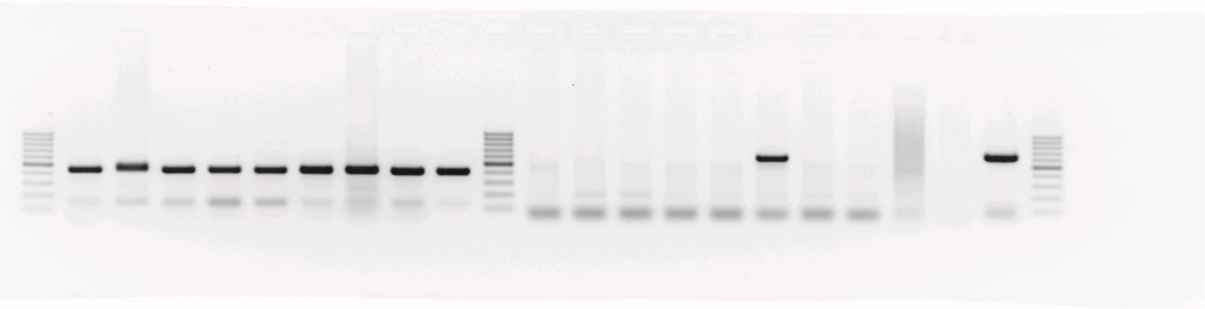

Gabra genotyping PCR

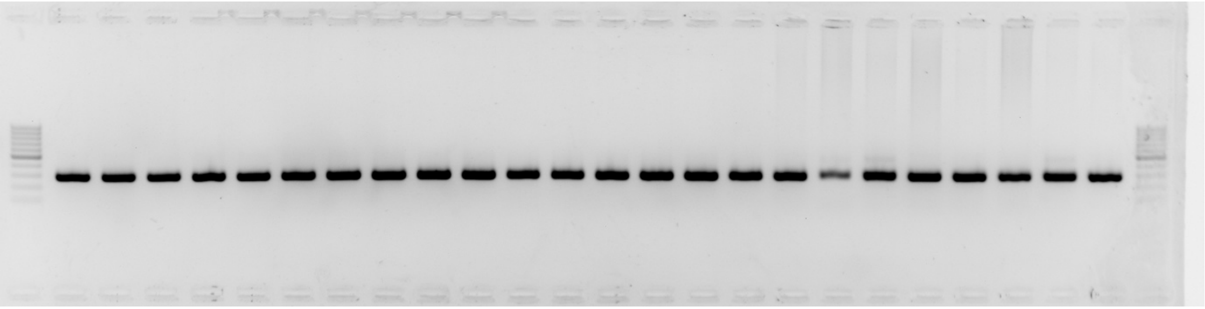

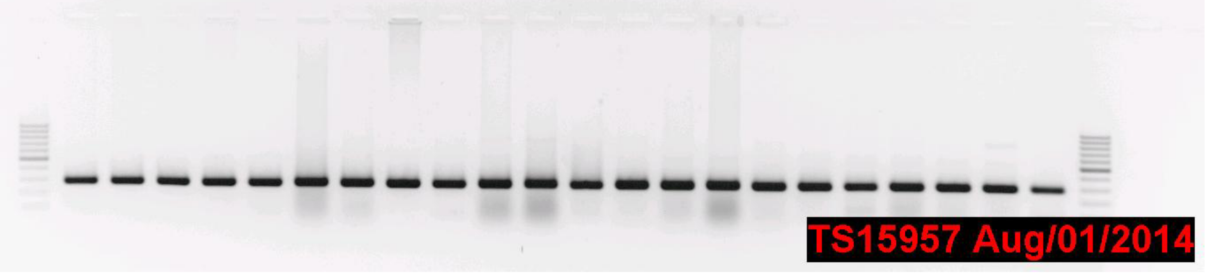

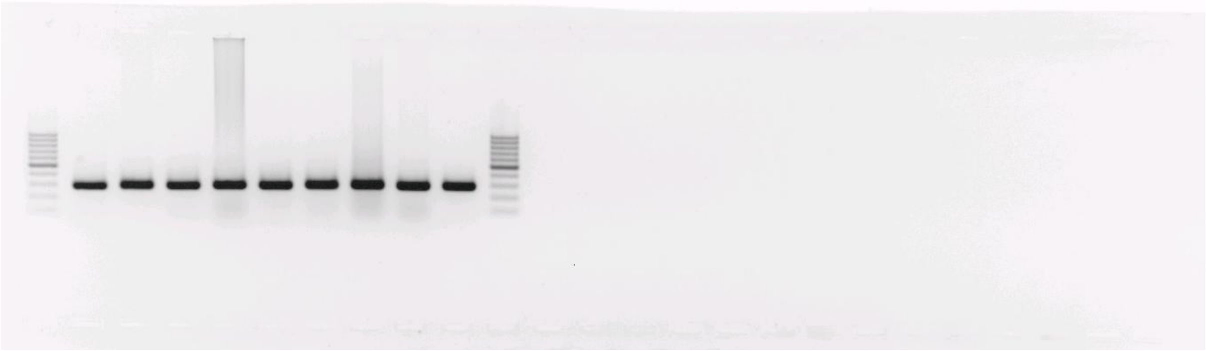

Source Data: Uncropped Gels of Supplementary Figures

Supplementary Figure S1

Panel S1b
Southern blot

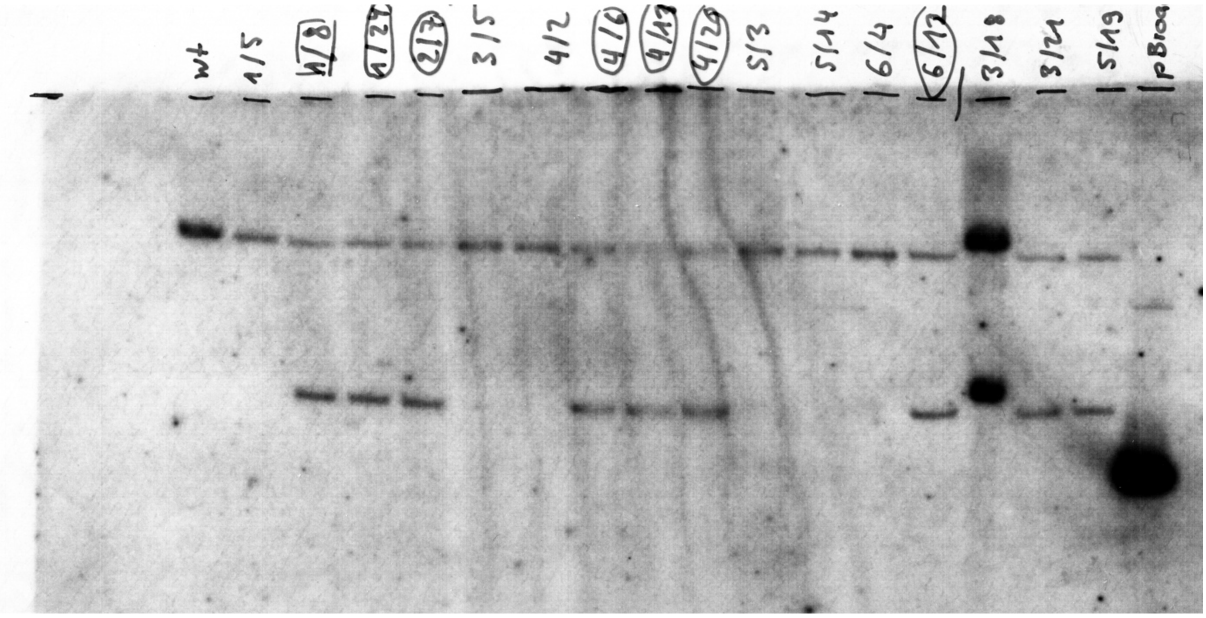

Panel S1c
Genotyping PCR Snail^{KI} model

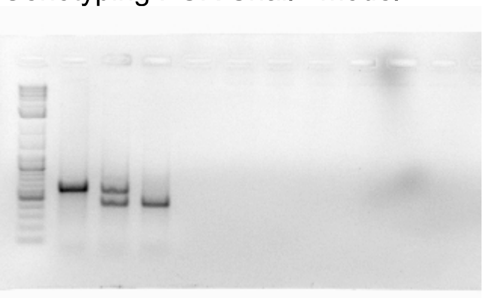

Panel S1d
Recombination PCR Ptf1a-Cre;Snail^{KI} model

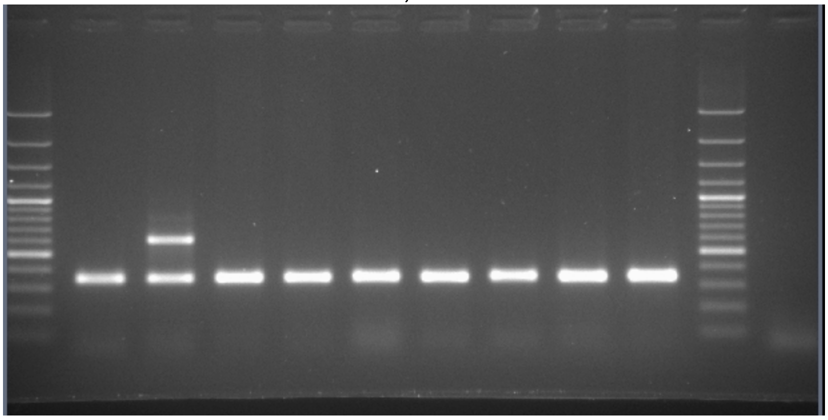

Supplement: Supplementary file 6 — Source Data [file 41467_2023_36505_MOESM6_ESM.zip › Source_data_unprocessed_gels-FINAL-all.pdf]
